# Large-scale solar magnetic field mapping: I

**DOI:** 10.1186/2193-1801-2-21

**Published:** 2013-01-23

**Authors:** Kenneth H Schatten

**Affiliations:** Ai-solutions, Inc, Suite 215 10001 Derekwood Lane, 20706 Lanham, MD USA

**Keywords:** Sun, Solar dynamo, Magnetic fields, Sunspots, Solar activity, Solar-terrestrial, Heliosphere

## Abstract

This article focuses on mapping the Sun’s large-scale magnetic fields. In particular, the model considers how photospheric fields evolve in time. Our solar field mapping method uses Netlogo’s cellular automata software via algorithms to carry out the movement of magnetic field on the Sun via agents. This model's entities consist of two breeds: blue and red agents. The former carry a fixed amount of radially outward magnetic flux: 10^23^ Mx, and the latter, the identical amount of inward directed flux. The individual agents are distinguished, for clarity, by various shades of blue and red arrows whose orientation indicates the direction the agents are moving, relative to the near-steady bulk fluid motions. The fluid motions generally advect the field with the well known meridional circulation and differential rotation. Our model predominantly focuses on spatial and temporal variations from the bulk fluid motions owing to magnetic interactions. There are but a few effects that agents have on each other: i) while at the poles, field agents are connected via the Babcock - Leighton (B - L) subsurface field to other latitudes. This allows them to undertake two duties there: A) the B - L subsurface field spawns the next generation of new magnetic field via new agents, and B) the B - L subsurface field attracts lower-latitude fields via the “long-range” magnetic stress tension; ii) nearby agents affect each other’s motion by short-range interactions; and iii) through annihilation: when opposite field agents get too close to each other, they disappear in pairs. The behavior of the agents’ long- and short-range magnetic forces is discussed within this paper as well as the model's use of internal boundary conditions. The workings of the model may be seen in the accompanying movies and/or by using the software available via SpringerPlus’ website, or on the Netlogo^*TM*^ community website, where help is readable available, and should all these fail, some help from the author.

**Electronic supplementary material:**

The online version of this article (doi:10.1186/2193-1801-2-21) contains supplementary material, which is available to authorized users.

## Introduction

In this paper I examine the Sun’s large-scale magnetic fields and develop a new algorithmic model that describes how these fields may evolve from new field sources as they emerge onto the solar disk within active regions and how they move to their ultimate demise. I shall begin by making two basic assumptions: 1) that locality is obeyed: namely field entities respond only to conditions in their local environment; and 2) that the fields undergo the processes of birth, motion, and annihilation. These will be discussed more fully later. The actual model has detailed aspects involving solar cycle magnetic phenomena, but these two are the main bases of this model.

I next provide an introductory review of solar field models, and then describe observations of the large-scale solar field patterns. Following this overall introduction, I shall then discuss the modeling bases, more detailed modeling aspects, and parameters. Details of certain aspects of the modeling are brought forth throughout this article, so neither the Appendix nor the article resembles a cookbook. One may find a zipped file of the program and needed files that run the model discussed herein: solfieldmapping.zip. The Appendix, however, does discuss and gives references to aspects of the relationship between moving magnetic fields and time-dependent flows; that magnetic field lines form “streaklines” not streamlines, as generally thought. Additionally, it discusses why field lines move towards uniform field magnitude, thereby lowering the total external field energy.

### Observations and models of solar fields

Hale (1908) identified the magnetic fields of sunspots and bipolar magnetic regions (. (BMRs). Smaller-scale models of solar field motions were developed prior to the large-scale models. Of course one of the main differences between the two is that in the latter case, one required a spherical, rather than a planar geometry. Van Ballegooijen *et al*^1998^) developed a “cork model” wherein fields in the model moved around the surface, driven solely by flows, which advect the field, much as corks placed on the surface of an ocean may be driven by surface currents. Their model used observations from high-resolution G-band and continuum filtergrams obtained at the Swedish Vacuum Solar Telescope at La Palma. Using these observations, the authors identified bright point motions associated with solar magnetic field and calculated their behavior amongst small granular size photospheric elements. Models of the granulation flow were derived from the observed granulation intensity images using a simple two-dimensional model that included both inertia and horizontal temperature gradients; the magnetic field elements were assumed to be passively advected by the granulation flow. The results supported the view that passive advection could explain the observations, indicating that on short timescales the field tubes are not strongly affected by any significant anchoring at large depth. In the solar atmosphere above the photosphere, the authors used potential field models, which allowed a wide variety of reconnections, and differing behavior compared with sub-photospheric motions.

Further developments in modeling field motions were continued by a number of researchers. For example, Schrijver and DeRosa (2003) modeled the large-scale fields of the photosphere and their extension throughout the heliosphere. These authors made comparisons from MDI aboard SOHO, using potential field methods. They investigated a number of aspects related to the large-scale connections of the solar field using a variety of data sources, along with tracing back the interplanetary field to solar features. It was noted that the interplanetary magnetic fields (IMF) traced back to magnetic plage outside of active-regions. The authors also conclude, that “no subtle field-emergence patterns or field-dispersal properties are required of the solar dynamo beyond those that are included in the model () in order to understand the large-scale solar and heliospheric fields.” More recently Schrijver, et al. (*their model*^2008^), and references therein, discuss small scale field motions, wherein rapid changes occur and result in field connectivity changes. These affect reconnections of magnetic field in the solar atmosphere, sometimes with global field connection changes higher up in the solar atmosphere. Because of the general relationships required for magnetic fields to reverse polarity at the poles, observed most commonly near solar activity minimum, there is a great interest in reconnections of magnetic fields. A secondary interest is that such reconnections near the Sun often spawn coronal mass ejections (CMEs) and these CMEs may be associated with geomagnetic storms, and other phenomena affecting space weather on Earth.

We consider large-scales on the Sun to indicate sizes of ~1/10 of a solar radius to a solar radius. Following Hale’s early observations of sunspots and bipolar regions, Babcock and Babcock (1955) discovered the “preceding - following” relationship between the bipolar magnetic region (BMR) field directions and the polarity of the Sun’s polar magnetic fields. The Babcocks also discovered the Sun had latent magnetism outside of activity centers. Babcock (1961) subsequently developed a model that illustrated how sunspot fields could be related to the large-scale fields found in the Sun’s polar regions. Leighton (1964, 1969) subsequently modeled the motion of fields using diffusion to develop his dynamo model, essentially based on Babcock’s viewpoint, but incorporating equations and calculations via a computer algorithm to generate a self-oscillating dynamo. The sunspot fields were placed in his model using “pseudo-random” inputs for the location of sunspots in photospheric low latitudes. The theory advanced solar dynamo understandings from near infancy towards a full-blown theory with one or two giant steps.

The largest field patterns on the Sun were found and called "unipolar magnetic regions" (UMRs) by Bumba and Howard (1965) who found patterns of field oriented as backwards “C-shaped” structures. The patterns of magnetic field surface structures had a nearly uniform field direction (radially outwards or inwards), known as UMRs. This is discussed further in this article’s Appendix 1. These UMR patterns were called “sectors” by Wilcox and Ness (1965), who assumed a strict North - South geometry; thus they first found the UMR patterns connected the solar fields with those observed in interplanetary space. On the modeling side, Newkirk et al. (1968) developed a large-scale model with internal spherical harmonics.

We now drift a little into observations of the interplanetary magnetic fields (IMF) and its relationships to solar fields. For many years observations of the Sun’s global, and particularly polar fields was non-existent. Hence, we had to deduce these fields from the IMF. The extended coronal magnetic field was addressed by Schatten et al. (1969) using fictitious monopoles to meet the lower boundary condition and the source surface method for the outer boundary condition. Advances in coronal field modeling continued with the work of Altschuler and Newkirk (1969). Cowling (1969) reviewed a number of aspects of the solar wind’s field geometry during this time period.

In the next decade, Pneuman and Kopp (1971) ascertained an MHD solution with volumetric currents for a strict solar dipole; a great advance in understanding much of the physics behind coronal field geometry. Schatten (1971a, b) developed a current-sheet model, noticing the similarities between solar helmet streamers and the terrestrial magnetosphere. He developed a method to calculate force-free magnetic field geometry for the corona using current sheets. This was diametrically opposed to the Pneuman and Kopp’s (1971) model. Currents were allowed only on infinitely thin current-sheets where it was assumed |*B*| = 0, so that *J* = 0 everywhere, as there were no volumetric currents. Additionally, current sheets may not be totally field free, as a shear zone may develop therein. There is now recognition that coronal fields have current sheets as well as volumetric currents, with Mikic’, Linker and colleagues see (Mikic’ and Linker, 1994). Hence, a “middle ground” has been found on the subject of currents in the corona. Additionally, the potential field source surface (PSFF) lives on, remaining a good workhorse to calculate heliospheric fields generally. Hoeksema (2009) has made improvements to the PFSS model and provided the data to many in the solar-terrestrial communities so that serves as a synergy that improves general knowledge about space-weather throughout the whole heliosphere.

The Stanford group popularized the geometry of the current-sheet view (e.g. Hoeksema et al.,^2012^) with an improved graphics picture. It was renamed for awhile as the “ballerina skirt” model. However, as time advanced, more politically correct naming issues followed and the name finally settled down to the “heliospheric current sheet model,” surprisingly close to its original name. This 3D graphics picture perhaps made its greatest contribution, not in understanding the large-scale solar magnetic fields, but rather how this magnetic field affects the transport of cosmic rays throughout the heliosphere.

Returning to the Sun’s global magnetic field and related field motions, one tries to understand what is occurring beneath the photosphere’s roiling motions. More recent authors have incorporated field transport via flow-driven motions (*e*. meridional circulation) to regenerate the dynamo. Many deep-seated dynamo models (see Choudhuri 2002; Brandenburg 2009Charbonneau ;2010, and references therein) have improved upon Leighton’s early dynamo model. They often use the observed meridional-flow motions to advect the magnetic field across the solar disk because the Sun's high conductivity, associated with Alfvén’s frozen-field approximation, generally has been used as a requirement that field and flow move together.

Questioning the overall view of the meridional flow structure having deep slow moving cells that circulated throughout the Sun, Hathaway (2012) is using supergranules as probes of the fluid motion below the photosphere. He reports that a return equatorward flow from the poleward meridional circulation occurs “just below the base of the surface shear layer.” This picture is attractive in a number of ways, particularly when one considers the large number of scale heights between the thin surface layers and the deep convection zone. This picture, together with the view that near the Sun’s surface, active regions may serve to transport low enthalpy gas inward as cold fingers of gas descend from the Sun’s surface deep into the solar interior (see Schatten and Mayr, 1985; Schatten 2009, 2012a and references therein). Hence, it may be that a shallow circulation pattern consists of a surface double-layer ribbon of flow. The main circulation patterns that numerous 2D and 3D models show (see Toomre et al. 2000), are required to transport energy outwards throughout the convection zone. When active regions develop, the shallow flow patterns may be penetrated by cold fingers of gas below active regions that descend through the shallow flows towards the bottom of the Sun’s convective envelope. In mixing-length theory, warm bubbles rise and dissipate with distances defined to be a mixing-length. Typically, this is taken to be on the order of an atmospheric scale height. Mixing-length theory says little concerning the fate of descending cold gases. It may be that such cold gas columns travel to near the radiative core. If any of the descending cold gas columns were to overshoot too deeply, the gas column heats up and is inhibited by the local temperature and pressure gradients being too steep. This steep gradient would cause a sharp rise in the temperature of a descending column, owing to adiabatic heating. Consequently such a column would rise due to buoyancy.

Sheeley et al. (1985) engaged in large amounts of vital work towards understanding the Sun’s global field; they review and develop Leighton’s work to model the real Sun, and these authors continue to advance numerical models of “real fields” and their relationship to interplanetary parameters. Zwaan (1985) showed that field elements in the photosphere would engage in a convective collapse leading to small radial field elements. This showed, as observed, the actual photospheric field is highly non-uniform. As our knowledge of the complex coronal field grew, Schrijver et al. (1997) discussed the solar/coronal field as a “magnetic carpet,” thereby illustrating a more complete picture of the magnetic field on a small scale size; namely it contains many tiny loops of magnetic field. This more accurately portrays the three-dimensional field aspects, as opposed to earlier, simplified 2-D geometry.

Examining the relationship of observed solar fields with calculated field models showed weak, but positive correlations, supporting Leighton’s model (see Schatten et al. 1972 and DeVore et al., 1984). However, as time passed, refined observations of field motions within the Sun’s surface found other field transport methods beyond diffusion were needed, for example, meridional flows. To this author, these seemed insufficient to understand the motion of solar fields, as this author’s view, as part of the current model, is that field-based flows are required. For example, if both signed fields were totally dominated by the same flow, then little separation between fields of opposite polarity might ensue. We define “field-based flows” as flows whose motions vary based upon the sense and/or magnitude of the embedded field. In any case, further achievements were made in understanding how magnetic fields are transported on the Sun's surface. The amount of magnetic flux that active-regions generate and transport to the Sun’s polar regions, during a single solar cycle, was found to be roughly one part in a thousand (see Wang et al. 2002; Wang et al. 2005). For example, during the 1970s approximately 1–3 ×10^25^ Mx were released per solar cycle, compared with a polar field, at the peak of the Sun’s polar magnetism, near 10^22^ Mx. This number, 1:1000 roughly, is a number that I will be using throughout, to compare our model to the Sun’s behavior. This is a number well known to Babcock (1961), who said a “field rope” could yield 1000 BMRs (bipolar magnetic regions).

Our understandings of both the interplanetary and solar components of field magnetism were greatly advanced by Sheeley et al. (1987), who found that the global field patterns are consistent with magnetic field emanating from field sources in active-regions. In addition to the consistency with potential field models, their model also showed that the rigid rotation of large-scale fields is consistent with the puzzling aspect that differential rotation of smaller-scale active-region magnetism occurs concomitantly with solid-body rotation of the large-scale field patterns in the coronal and interplanetary fields, namely sectors.

Their important findings related to magnetic field motions may be viewed simply as follows. Differing rotation rates can exist between the motions of a pattern (similar to “proper motion”) and that of the actual fluid’s material velocity. This is similar to the more familiar differences of velocities of ocean wave patterns that travel towards the shore at the wave speed (the celerity of the wave); however, the actual motion of a water molecule is approximately a stationary circular motion. The changing field patterns in the corona do require some reconnection events to continually occur, so as to allow these field patterns to remain stationary while the individual fields rotate underneath, as viewed in the pictorial model of Fisk et al. (1999). Wang et al. (1989) also furthered Leighton's general solar field model by studying the surges of field to the poles, which move seemingly more from flows, rather than from diffusion. For example, field surges often contain magnetic flux of both polarities. Sheeley (2005) and references therein discuss many aspects of magnetic fields, both on the Sun and in interplanetary space. Additionally, this paper adds to our understanding of solar field patterns, flows, and the solar field diffusivity.

One aspect of the present article may shed light on a problem raised concerning high latitude magnetic fields and converging flows. Magnetic fields below the photosphere have generally been associated with deep-seated solar dynamos. Recent work in this vein, involves studies by Ulrich and Boyden (2005) who were able to observe and describe the surface manifestations of the difficult to observe, toroidal magnetic fields. Polar fields are often described by the high-latitude formula: ∝ 11*G* sin ^8^*θ* is the heliographic latitude, as used by many in this field, however, little is known about the photospheric toroidal field. Ulrich and Boyden used a combination of extended observations together with creative analyses to advance our understanding of the interplay between poloidal and toroidal magnetic fields in the photosphere, particularly at high latitudes. Their innovative article displays variations in the toroidal fields that illustrate the manner in which these fields vary with latitude in a synoptic-type map, similar to the more familiar butterfly diagrams of active regions. The variation of these fields, as well as those of meridional flow, occurs predominantly at high latitudes, which these authors attribute to the convergence zone of the meridional flow. This makes the valuable point that the polar regions of the Sun may be a key to understanding the solar dynamo. Nevertheless, even though the flow does converge in the photosphere, we still do not know at what depth the “return flow” occurs. Is it near the shallow layers, or deeper near the tachocline? Hathaway (2012) supports the view that the equatorward return flow from the polar directed meridional circulation occurs near depths > 50 Mm - just below the base of the surface shear layer.

With *both* the magnetic field and the flow converging at high latitudes, it is difficult to distinguish which physical parameter predominates: the magnetic field or the flow? Ulrich and Boyden suggested that the converging flow at high latitudes predominates. Perhaps, another question is more important to answer: what are the relative roles of the flow vs. the polar field? One cannot answer this intriguing question positively without further observations of the Sun’s polar regions, which might help us understand the solar dynamo. Perhaps because the poles have, presumably the least amounts of solar activity, these high latitude regions may be called the “ignoro-latitudes.” It may be, however, that although not magnetically "active," they are the most important regions of the Sun to study if we wish to understand the interplay between poloidal and toroidal magnetic fields, since only the latter are well observed from Earth.

Turning now to the subject of this paper’s research, the current model does not deal with “true” solar fields, with its myriad of complexities (e.g. the magnetic carpet), but rather considers magnetic elements in a simplified fashion, using simplified field “agents,” whose properties we shall discuss in the next section. Let us pause to reflect upon the nature of solar magnetism, that nature invariably overwhelms us with its complexity, just as a mathematical fractal can never be completely portrayed. Sometimes with mathematics, as with nature, the best one can do is to gain a highly simplified understanding. The author recognizes that the current model may, at best, be doing this and at worst, failing miserably.

I use Alfvén’s frozen-field approximation to ensure that on large-scales (*e*.*g*. the co-rotation of the fluid around the axis of the Sun), the movements of the large-scale field (and flow), in a neighborhood, are directly linked together via large-scale motions in which the field moves concordantly with the fluid. This is done by moving each entity in longitude and latitude appropriately. As I discuss subsequently, when I discuss the motion of our field entities, I shall be primarily considering motions relative to the steady-state fluid background motions. The background motions are simply the observed differential rotation and the meridional circulation. They are the mean motions of the photospheric gas. Although these are incorporated into the model, the more interesting aspects relate to motions relative to the fluid. The model shall be considering field-induced differences from these mean motions. The field motions are the sum of *both* the mean advective motions, and the added motions, which are temporally and spatially variable, that the current model calculates. These motions involve field induced forces, nevertheless the motions of the fluid and the field follow the sum of *both* the mean advective motions and the transient motions, as Alfvén’s frozen-field approximation requires. I next discuss an overview of the model here, with the details left within the Additional file 1: (Solar Field Mapping Model Information and code.doc).

### Model overview

I provide a brief synopsis of the model’s workings; further details of the code development may be found in the supplementary material: Additional file 1, entitled “solar field mapping model development information and user code”. Rather than reading all the details of the code, however, I whole-heartedly endorse running it instead, from this site: http://ccl.northwestern.edu/netlogo/models/community/Solar%20Field%20Mapping%201p07. The code may also be run in other ways: 1) one may find the software at: sourceforge.net at the site: https://sourceforge.net/projects/solfieldmapping; if one has difficulties with either of the previous methods, either contact me or obtain free help from the Netlogo user community website; or 2) one may choose to obtain the program within the zipped file, which uses the html applet: Solar Field Mapping 1p07.html with the Netlogolite java program, attached therein, or 3) using the attached NLOGO program (Solar Field Mapping 1p07.nlogo) with the full Netlogo4.1.2 version, which allows a faster running of the program. Additionally, for details of code design, along with the bare code from Netlogo’s software, one may find in Additional file 1 as a document file: Solar Field Mapping Model Information and code.doc.

The behavior of the model may also be viewed without the code by looking at the two movies (in Additional file 1: Movie1.mov and Additional file 1: Movie2.mov) showing some sample runs made with my program. These movies play with Apple's Quicktime movie player, and the movies may be slowed, stopped, placed in a loop, or controlled frame by frame. The movies allow one to see the field “flocking motions” where like-field entities move concordantly. It is this aspect that is a dominant feature of the field mapping model: it allows like-field entities to move within the photosphere en masse in a concordant fashion. This, seems different from diffusion models, where like-sign features dissipate. In the present model, these like-sign field entities form streams of magnetic field, wherein like-sign field agents move together in coherent patterns suggestive of Bumba and Howard’s UMR structures. To this author, the coherent field movements in the field mapping model differ from the diffusive behavior in Leighton's model and in the NRL group's simulations. Similarities and differences may be tested in the smelting furnace of mathematical calculations. Diffusive behavior has a number of hallmark properties: the timescale is proportional to the spatial distance squared and inversely proportional to the diffusion coefficient. In diffusion, initial differences slowly dissipate. Some of this may exist within our model, however, there are notable differences: namely, this model has field patterns that evolve leading to magnetic field changes in the Sun's large-scale magnetic field. Perhaps what is most notable is that the field mapping model allows initial purely bipolar field regions evolve into large-scale unipolar field, with wide field separations. It is our hope that these patterns are similar to the UMRs detected by Bumba and Howard. Regardless of the correctness of this work, one wonders how can solar physics we explain the evolution of the Sun's UMRs. This seems to be fundamental to our understanding of the solar dynamo.

### Model’s bases and formulation

Here we discuss aspects related to the model’s formulation and assumptions: 1) that locality is obeyed: namely entities respond only to conditions in their local environment; and 2) that the fields undergo the processes of birth, motion, and annihilation. Concerning 1) locality means that field entities may be subject to influences from their neighbors (a small region around which other entities are “seen,” and their properties – they may move towards or away from each other for example), as well as the fluid motions they are bathed in (the flow advects all field entities), and another “local quantity” is the subsurface Babcock-Leighton (B-L) field. Although this is calculated from the strength of the polar fields in the model, and this is quite a distance away and we often refer to it as a "long-range" force, we assume that the B-L field connects to field entities in the photosphere, so this "long-range" force is considered to be a “local effect.” The azimuthal Maxwell stress force is subsumed by the observed differential rotation (Newton and Nunn, 1951), thus we have ignored it for expediency. Item 2 will be discussed later. Additionally, the present model does not concern itself with small scale motions, for example the granular and even the super-granular motions. Instead, to simplify the field motions, I take the Sun to be a continuous smooth spherical surface. The previous researchers found narrow intergranular lanes, meaning that the field is arranged along these narrow lanes, as observed, via bright points, etc. with much of the photosphere being field-free. These granular aspects and networks are refinements that are unfortunately not included in the present model; however, other "granular" aspects will be included.

What is the basis that allows one to turn an inherently 3D problem of fluid motions containing magnetic field into a 2D problem? It is known that this thin layer, the photosphere, is greatly affected by the Sun’s deeper layers, as well as having many complex structures (e.g. granules, sunspots, faculae, etc.) within its volume and outer boundary. Thus the photospheric layer is anything but a simple spherical shell. Photospheric complications abound, including those involving radiative transfer, spatial inhomogeneities, temporal dependencies on many timescales, the possibility of vertical flows that seem to exist below sunspots (downward) and faculae (we presume upward) that may transport energy vertically, as well as the great turbulence associated with the convective flows, other complications such as the interactions of flows with each other. The turbulence in the photosphere is so large, that photospheric elements are driven to near sonic speeds of ~2 km/sec! How can one simply slip a spherical shell around the photosphere and then turn a blind eye to the realities of the physics within the surrounding layers? To understand the current method, we need to step back from the *complexities abounding within the Sun’s* surface to consider, in general, how one may restrict any model to any particular volume of one’s choosing, and virtually ignore volumes containing important phenomena that lie outside the chosen volume?

The author answers this last question with one, undoubtedly overly simplifying assumption. We choose our model to be like justice: blind. The model's workings are blind to the complexities of the problem, and follow tenets of basic physics. The laws of physics are obeyed *locally* as well as *globally*. Hence one may *always* choose *any* volume to be the volume of interest. This is what allows various symmetries to be employed when one takes advantage of a preferred geometry. For example, there are various problems that are simplified by choosing volume elements within spherical shells, cylindrical shells, rectangular coordinates, and other geometries for best advantage. By such a volumetric choice, however, one must adapt the “boundary values” to the surface elements surrounding the various volume or volumes of interest. A careless choice may result in boundary value problems and lead to wrong results. Nevertheless, one is always free to choose any volume for consideration that one wishes. Our use of the Sun's polar fields to be both: 1) the causal agent giving rise to the next cycle's field agents, and 2) the magnetic tension that redirects the next cycle's field agents, illustrates that we are not skimping on the importance of the Sun's internal workings to its surface behavior It is through our introducing these two properties that the interior dynamics of the Sun are manifested in the field mapping model. It may be that we err in choosing the lower region's boundary conditions as we have; however, it is not with a wanton disregard of the importance of the internal workings of the Sun. After all, we also recognize that the photosphere owes its luminosity, its gravity, etc. to the physics of the Sun's interior.

Before discussing our choice volume, and the field elements that lie therein, let me discuss how we shall treat turbulence within the photosphere. Although photospheric turbulence is immense, let us consider the photosphere in its absence. If fields were to arrive in the photosphere, and the surface were as placid as a lake, then opposite-sign fields would arrive there, be undisturbed, and could reconnect without being separated by the turbulence. Hence this model takes the view that it is the interplay between turbulence (tending to create disorder) and the Maxwell stress tensor (tending towards order) that controls the motion of the magnetic field agents. This allows us to utilize two scale sizes, and these boundaries divide space into three spatial regions. We set the smallest size as a parameter, *kill*-*dist*, within which (L *<kill-dist*) the field entities are assumed to annihilate each other if they are of opposite polarity, because we have chosen each flux element to have a constant magnitude. The largest size, we simply choose to be on the order of a solar radius, so that within these two distances, an external intermediate scale-size region exists, (*kill-dist* < L <≈solar radius). Within this region flows and field interact such that the flows separate magnetic field into unipolar regions. Within such regions flows and field interact such that the two sign field structures (inward and outward directed field) behave like immiscible liquids, such as oil and water. Hence, in the current model, flows and field form separated like-sign magnetic structures. It is the author's hope that these field structures behave similar to Bumba and Howard's unipolar magnetic regions (UMRs).

The author envisions that the field mapping model will transform magnetic entities in the modeled photosphere as follows. New sources of magnetic flux first develop within new bipolar magnetic regions (BMRs), while older magnetic elements are motivated to lower the total field energy by being transported towards regions of lower field magnitude (hence towards a more uniform magnetic pressure). It may be that the "magnetic network" plays some role in this process, which we do not consider. Lastly on the largest scales (L >≈solar radius), the magnetic forces supersede the smaller-scale motions and the attractive Maxwell stress tensor supersedes the shorter-range forces; this allows large-scale Maxwell stresses to again play a role. Within this third region the field mapping model allows *the subsurface B – L magnetic field* to draw opposite sign field elements towards the largest scale fields attracting them, which I choose to be the opposite sign magnetic pole. Thus a red field agent is attracted towards the blue polar field, and vice-versa. This is carried out in a straightforward fashion by use of the tension from the Maxwell stress tensor. The strength of the Maxwell stress tensor is chosen to be proportional to the quantity *B*_*N*_ − *B*_*S*_, obtained from the polar fields. Although we are discussing these regions from the viewpoint of these stresses/forces, the model additionally employs the other common forces as well: Coriolis forces, fluid motions, etc. to calculate how the field elements move, so as to obey the previous prescription. Nevertheless, the author finds it helpful to consider the nature of these primary forces which play great roles in the field motions. The author recognizes that although the large-scale B-L magnetic field is pervasive on a global scale (likely from being twisted up azimuthally by internal differential rotation), to exert a force on a small-scale entity, it must act locally, namely by its magnetic field making contact with a surface field element. The detailed actual code for how these "forces" are implemented in the model is available for anyone to improve on the author's methodology.

There are a couple of other aspects worth noting about the present model. I recognize that the photosphere is a weakly ionized region of the Sun's outer atmosphere. It transports vast amounts of convective energy, which flows from inside the sun to this thin surface. Within this surface, convective energy is transformed with high efficiency into radiant energy, which emanates from the thin photospheric gases out into space. As such, the methodology about how to model such a region is uncertain. The author has thus relied upon the knowledge gained from numerous observers who have studied the Sun. I owe them a debt of gratitude, and hope that the present model, which may be called "an algorithmic approach,” mimics some of the complex flows that the solar material engages in.

That being said, let us describe the model's method to calculate the flows of photospheric material. There can be forces from inside the photosphere, *i*, and outside the photosphere, *o*, upon elements within the photosphere. In the previous paragraphs, we discussed the forces within and from below the photosphere. Now I consider the force from fields outside the photosphere. I simply assume they are negligible, essentially considering that the outer magnetic fields are force-free. I shall explain the reasoning. Within the photosphere, the magnetic field occupies only a tiny fraction of the solar surface, from regions such as sunspots, pores, and ephemeral regions. These regions represent a tiny fraction of the Sun’s surface area, <1%. One can roughly estimate this by the variations of the solar irradiance. Above the photospheric surface, the magnetic field is predominantly thought to splay outwards, so that it fills the corona with magnetic field, essentially everywhere. Hence in the inner corona, the magnetic field changes from filling a tiny fraction of the area, <1%, to playing a predominant role, ≈100%. This changeover happens in the small distance between the photosphere and the base of the corona; hence the magnetic field of the inner corona essentially “fills space,” so that the corona is predominantly governed by the magnetic field and magnetic forces. This occurs, of course, through the changeover in the plasma β, from a high to a low value between the photosphere and corona. This changeover is what has allowed “potential field models” to be used to calculate coronal and interplanetary magnetic fields solely from knowledge of fields within the photosphere. A potential field is essentially a force-free field as the magnetic stress tensor is “balanced” in this field, with the field tension being offset by the field pressure, in potential field geometries. For example, the field of a bar magnet only exerts a non-zero force within the iron of the magnet.

Let us consider how the choice of a photospheric volume may affect our model. By choosing a small (≈spherical) shell surrounding the photosphere as the volume to examine, the model contains elements of strengths and weaknesses. One of the strengths is that the model stays well away from the areas of solar physics which have the least understanding, namely the deeper regions of the Sun. Our model instead employs a number of the mainstays of solar physics (Hale’s laws of sunspot polarities, and other active-region field attributes – Joy’s law, etc.) that are better understood, or at least that we are familiar or comfortable with. One of the weaknesses then, is that the field mapping model really has little to say about what goes on outside its domain - a small volume just enclosing the photosphere. Hence our model should remain silent about what goes on well beneath the photosphere. Nevertheless the author expresses hope that a model such as this may serve as a test as to whether one may sweep the physics of the deeper solar regions under the “photospheric rug,” allowing one to calculate within the confines of a fixed volume, the behavior of the Sun for limited periods of time. Other aspects of the model's active regions are highly, simplified: the regions have all the same + and – amounts of magnetic flux. Variations in the dipole moment of bipolar magnetic regions (BMRs) may be established by the distance between the preceding and following poles, as well as the number of field entities per active region and frequency of active region births. Thus some of the known solar physics has been simplified. The question is whether our methods and algorithms are able to capture the vital aspects of the Sun's large-scale magnetism, or whether the current model or even a revised version is simply inadequate to mimic solar magnetic field behavior. Overall, the model may be understood simply as follows: field entities move towards regions of lowered field magnitude, as well as moving concordantly with like-sign field neighbors. This is the magnetic equivalent of heat moving towards uniform temperatures. The model has some simplifying assumptions (e.g. granularity obtained by quantizing field entities, etc.). Details of the model are found in Additional file 1.

The focus of the current model is the magnetic field of the photosphere. We have given reasons why we have chosen to simplify the surrounding plasmas. Let us consider how the plasma and magnetic field interact in these regions. It is helpful to keep in mind that some of the physical behaviors of the Sun’s magnetic field relate to the plasma β, the plasma pressure to magnetic pressure ratio. This is the most significant factor controlling the interactions between plasma and field. Other forces are at play as well. For example, the photosphere is a weakly ionized plasma, and thus some of the aspects of gas dynamics, familiar to meteorologists, also pertain to understanding the fluid motions, and thermodynamic properties of these gases. Within and below the photosphere (outside of sunspots), the value of β is high; consequently flow pressures, rather than magnetic forces, predominantly affect the fluid motions. Nevertheless, subtle fluid forces, involving the field do exist, such as Zwaan’s convective collapse of field into spatially large regions with near zero field, also containing small regions having large field magnitudes, balancing the plasma pressures, with either radially inward or radially outward field. It is because of these findings, that we have chosen the dichotomy of our field entities, having specific regions of large field and regions having near zero field. Driving fields in this fashion requires, of course, non-linear aspects in the equations, similar to the workings of a transistor in a non-linear circuit, driving voltages to saturation levels. Hence we sought the need for non-linear mathematics to describe the interactions of solar magnetic fields with flows. This led us to seek ideas from the methods and mathematics of cellular automata, which allow for the entities within their systems to behave non-linearly very easily.

Although our interest in solar physics began with the corona and solar wind, the present model predominantly disavows interest in the corona and solar wind, just as it disavows interest in the physics of the deeper solar layers. Although the deeper layers contain material that has higher densities, temperatures and pressures, and thus are in a better position to affect photospheric phenomena, we are also more ignorant of what goes on there. The effects of the Sun's deeper layers are virtually subsumed in this model by two boundary conditions that the model uses: namely that new magnetic regions arise in a proportionate manner to the previous cycle's polar magnetic flux; and it is also this same field that pulls opposite sign photospheric magnetic entities towards the Sun's polar regions. Hence, for pragmatic reasons, this paper shall be concentrating on photospheric phenomena, with the exceptions being that we shall allow the deeper layers to exert changing boundary conditions upon the photosphere, by 1) having new activity well upwards into the photosphere in the form of bipolar magnetic regions (BMRs) and 2) allowing the magnetic tension of this magnetic field to pull on magnetic entities of the opposite sense field. This is how the present model shall be working. The deeper layers of the Sun also play a role in the differential rotation of the photosphere, and the meridional circulation, both of which we assume are constant, despite the knowledge of the torsional oscillations seen by LaBonte and Howard (1981). These are, however, distractions to the present model, which might just add a complexity that inhibits progress that can be made in understanding photospheric magnetic field evolution, by casting aside layers outside the photospheric domain. One can make some progress, sometimes when one is overwhelmed by a problem’s complexity, by simply casting aside the difficult parts and allowing a solution to the simplified problem emerge.

To summarize the workings of this model then, one can think of the present model, as allowing the magnetic fields to move towards their lowest external energy states, by having newly situated magnetic fields move freely on the Sun’s surface towards those areas of weakest magnetic field magnitude. In the Additional file 1, I discuss the simplifying basis through which I turn the inherently 3D problem of fluid motions carrying magnetic fields to the Sun’s photosphere into a 2D problem. Basically, I am assuming that magnetic fields are brought to the photosphere, and then can move on the Sun’s surface towards regions of lowered potential energy. This is one aspect of the Solar magnetic field mapping model. Additional file 1 discusses detailed aspects of the model; running the model, along with the code.

Rather than differential equations I use cellular automata agents. Put succinctly, our model can be explained thusly: agents are born in active-regions through our random number generator, move about on the solar surface by a number of flows and forces I describe, and are annihilated. There are but a few effects agents have on each other: i) while at the poles, magnetic field agents are connected via the Babcock - Leighton (B - L) subsurface magnetic field to other latitudes. This allows them to undertake two duties there: A) the B - L subsurface magnetic field spawns the next generation of new magnetic field, and B) the B - L subsurface field attracts lower-latitude fields via its long-range magnetic tension; ii) nearby agents affect each other’s motion through short-range interactions; and iii) through annihilation: when opposite magnetic field agents get too close to each other, they disappear in pairs. Although we identify the magnetic tension as a “long-range” force, we recognize that this force is transmitted through a local interaction, when the sub-surface field pulls on an opposite sign field agent in the photosphere.

Why do I use cellular automata rather than differential equations to model the large-scale solar magnetic fields? Briefly, it is my view that magnetic field generation is highly non-linear. With such systems, it turns out, that cellular automata may be a better way to handle the solutions than differential equations, owing to their ability to handle non-linear behaviors which differential equations often cannot handle, particularly those worst case scenarios where the function is non-differentiable! Aspects of the cellular automata (CA) uses can be found in books and literature by Wolfram, and other authors, or by viewing their uses in non-linear phenomena, such as wildfires, Ising spin-states (domains) in solid state matter, *etc*. where particles interact with their neighbors in a non-linear fashion. In these cases, and in many others, the computations cannot be done well with differential equations, because things go abruptly from one state to another, without a smooth transition. The non-linearities just dominate. In such cases, one may call such behaviors by any of a variety of names: one may say there is hysteresis (for temporal phenomena), or “symmetry breaking” for aspects involving spatial non-uniformities, or with changes in the properties of elementary particles, etc. Nevertheless, mathematically, what they all amount to are sharp changes in properties that simple differential equations cannot handle.

With a number of solar phenomena generated suddenly, e.g. flares, sunspots, divisions of the Sun into coronal holes and unipolar magnetic regions (UMRs), which seem to contain “boundaries,” it may be that gradual equations (amenable to differential equations) are more appropriate for some phenomena and cellular automata for others (generally where conditions, either spatially or temporally, change dramatically). In any case, the use of cellular automata is the path I have chosen to model solar magnetic fields, in the hope that this usage may lead to new understandings of the non-linear behavior of solar magnetism in the future. I point to the formation of active-regions from magnetic fields inside the Sun as but one example of a rather abrupt (and likely non-linear) phenomenon that is germane to the subject under investigation.

For this model, I simplify the Sun’s magnetism into just two “kinds,” inward and outward magnetic field orientations for a number of reasons: i) the Sun's magnetic fields seem to be approximately quantized into having either ~0 magnetic field or near kilogauss magnetic field owing to the convective collapse of photospheric material (Zwaan, 1985), with the field oriented either radially-inwards or -outwards; ii) scientists in the field of non-linear phenomena find that increasing the “granularity” of the agents actually helps one obtain solutions to various types of numerical problems, and iii) this usage (as opposed to giving various agents different amounts of magnetic-flux) allows the magnetic fields that our agents are imbued with, to completely cancel when two opposite breed members interact. This methodology saves untold difficulties which might arise from partial interactions, such as growing numbers of smaller field sources. In the model, we incorporate the fact that real fields on the Sun do have differing amounts of magnetic flux, by assigning different numbers of field agents; each agent though carries an identical amount of magnetic flux, either inward or outward pointing. Thus the model simplifies the various complexities which arise when magnetism is not so constrained.

I shall use an environment specifically designed for agent-based models, Netlogo. The genesis of the present program began from Craig Reynolds’s flocking program, in the Netlogo^*TM*^ language (Wilensky 1999a). Utilizing two identical but opposite sense fields as “breeds” in the Netlogo language helped the model along its path to fruition. In this language, two different types of agents are called “breeds.” Here, the two breeds are basically of identical form, but can, with this breed distinction, recognize any other member as being a same breed member, or an opposite breed member. In the same way that opposite magnetic poles of bar magnets show diametrically opposed attractions, the behavior of different fields in the photosphere behave quite contrary to traditional magnetic fields in a vacuum. For example, in the early days of an active region (during its growth phase), we see same sign magnetic field in the photosphere attracting like-field, and opposite field being repelled from each other (contrary to vacuum electromagnetic theory). On the other hand, on large-scales, and/or when an active region's growth phase has subsided, one observes “normal” behavior of magnetic entities: e.g. on large-scales opposite fields attract each other (e.g. the polar fields attract opposite sign entities via the subsurface B-L field), and same-sign fields spread apart into a more homogeneous field (a UMR). Our breeds will consist of two generic entities. The two breeds have different colors, blue and red, representing outward and inward pointing magnetic fields respectively. The direction any particular agent is moving, relative to the fluid, will be shown by the direction of the field entity's arrow. I shall be discussing their properties, in particular their birth, disappearance, and movements. Let us first be pragmatic and discuss the Netlogo domain that is their home.

### Netlogo agent-based model interface

**Figure 1:**
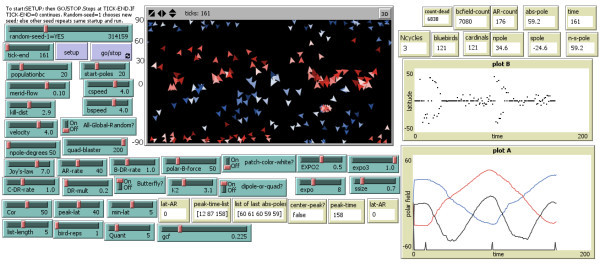
**Netlogo Interface for nominal “Solar Field Mapping 1.07” model at 161 ticks time, with random seed 314159.** The Display in the top middle is this model’s synoptic map of the photosphere, with the field entities shown. The display uses a Mercator projection, rather than the more common, solar equal area projection; consequently we have foreshortened the arrow sizes with latitude, to help illustrate this. Each "arrow" represents a "unit" of photospheric magnetic flux, 10^23^ Mx per blue or red arrow, and is pointed in the direction the flux is moving, relative to the fluid. The blue arrows are outward directed flux and the red, inward flux. On the left are the gray Setup and Go/Stop buttons to start the program. Blue slider bars and on-off switches allow the user to vary the model parameters without reprogramming. The white output monitors show numerous model outputs. The speed bar above the interface allows the model to run faster or slower. On the lower right side, plots A and B provide a synopsis of the run. Plot A shows the North polar field (Blue line) and South Polar field (Red line), and total Polar field (Black line) offset by 60 units, to improve the appearance. The tick marks at the bottom of this plot show polar field maxima, equivalent to sunspot minima. Plot B shows the latitude of new active regions. There is a graphing quirk; one cannot show two values at the same time, nor skip a value, so the graph plots a zero then. Hence the graph does not appear like a traditional butterfly diagram.

Figure1 shows our “Solar field mapping 1.07” interface, having run with the Netlogo 4.1.2 program (Wilensky 1999a), at the end of 161 *tick*s. The program may be found by clicking on: http://ccl.northwestern.edu/netlogo/models/community/Solar%20Field%20Mapping%201p07 or by downloading “Additional file 1,” which contains the program and instructions. Examining the interface of Figure1, one sees the following attributes: the Display map is in the top center, and its geometry is discussed later. Plots A and B are off to the right, slider bars, on-off switches, as well as monitors, are all discussed subsequently. To start any run, one must click SETUP, which initiates a few random polar fields; this prevents division by zero, since averages over the interface are performed. Next, the GO/STOP button starts evolving the model in time. The display, situated in the top-middle of the interface shows the blue/red fields as they migrate across the solar disk. The two graphs on the right display average properties of polar fields (Plot A) and the latitudes of new regions (Plot B) per time step, as the model evolves. Above the Display are a number of generic added features of Netlogo, e.g. a slider bar to speed up the entire processing, however, at high speed the display itself does not keep up with the timesteps, owing to the huge volume of features required to redraw. Let us now go through the features of our particular solar field mapping model and its usage in Netlogo. The rectangular field of the Display upon which agents move may be connected from top to bottom and/or from the left to right. Most naturally, for the Sun, one is most likely to emulate the photosphere: a spherical surface, with rotational poles defined to be at the top and bottom in our model, since the Sun’s axis may be defined to run North - South. Hence I connect the left and right meridians, so the right edge connects with the left edge, allowing field entities to cross there. There is however a disconnection from top to bottom, so the poles are separated. I also prohibit the agents or creatures from going any further in the y-direction than 90 degrees. The model’s display is Netlogo rectangular format; this most easily converts to a sphere by considering the display to be a Mercator projection. A spherical surface has an Equatorial circumference of 2πR and a pole to pole distance, on its surface of πR. To prevent fields from going “over the poles,” I chose our world to be a vertical cylindrical geometry (isolated at both poles). The coordinates are zero axes with grid-units of ±42 unit xcoordinate frame, and ±21 unit y -coordinate frame, keeping the 2:1 ratio in the spacing (the distances above), keeping the 2:1 ratio of a sphere. Counting the zero axes, as well as the non-zero gridlines yields 85 horizontal gridlines together with 43 vertical gridlines. These gridlines do not limit the fineness of field entity motions; they move with high precision motions. The important aspects are the distances between the grid lines, the 2:1 ratio. Nevertheless, the Mercator projection does expand areas at high latitudes, as one learns in public school. Modern solar maps are usually equal area synoptic charts. To offset this deficiency, the size of field arrows is reduced (inversely proportional to the square root of sine latitude) with latitude. It has no effect upon the field motions, but offsets the Mercator size distortion. Thus, although all arrows in this model possess equal magnetic flux, the arrows are drawn smaller at high latitudes to offset the expanding geometry. Figure1 shows our “solar field mapping 1.07” interface, having run with the Netlogo 4.1.3 program (see Wilensky 1999b), at the end of 161 ticks. The 1.07 refers to the 7th revision of the basic mapping model. At 12, 87, and 158 ticks there are peaks found in the total polar field. From 12 to 161 ticks the solar cycle undergoes a complete reversal, the equivalent to ≈22 years, with the same sign polar field, positive in the North Pole returning. I note the following numbers. At 161 ticks, there are 6838 count-dead; 7,080 bcfield-count; AR-count 176, and abs-pole is 59.2. The number of live blue agents and cardinals on the Sun remain at 121 each, and the n pole and s pole fields are 34.6 and −24.6, as the 2nd cycle ends. I call this our Nominal Model. To run beyond any preset time, one may set tick-end to zero, and then stop the run at the desired point, or move the slider to a particular choice (in multiples of 100), or with Netlogo, one may modify the setting slider to adjust the slider to a different coarseness of the slider. The current model removes much of the gratuitous generation of magnetic field that destroys itself in short order, as "noise." By noise the author simply means active-region magnetic field generation followed by magnetic field destruction that the 1.07 in the model name refers to the 7th revision of the basic mapping model. I call this our nominal model, and typically have used 314 159 as our *random-seed*. If this variable is set to 1, then the model will start at different random-seeds with each run. At 12, 87, and 158 ticks there are peaks found in the total polar field. From 12 to 158 *tick*s the solar cycle undergoes a complete reversal, the equivalent to ≈22 years, with the same sign polar field, positive in the North Pole returning. In the following sentences, I use terms that denote specific parameters defined as special variables in the program, which are reported by the monitors in the Netlogo display as it runs. I note the following numbers (quantities specific to the program, referring to the number of agents of various types). At 161 *tick*s, there are 6838 *count-dead*; 7,080 *bcfield-count*; *AR-count* 176, and *abs-pole* is 59.2. The number of live blue agents and red agents on the Sun remain at 121 each, and the *npole* and *spole* fields are 34.6 and −24.6, as the 2nd cycle ends. The quantities *npole* and *spole* refer to the mean smoothed polar fields averaged, so that there is no sharp cutoff at any arbitrary latitude. To run beyond any preset time, one may set *tick-end* to zero, and then stop the run at the desired point by clicking GO/STOP, or move the *tick-end* slider to a particular choice (in multiples of 100), or with Netlogo, one may modify the setting slider to adjust the slider to a different coarseness, should one choose. Our model has removed much of the gratuitous generation of magnetic field that occurs near solar active regions, which then destroys itself in short order. The Sun yields, on average, one polar field line per 1000 active region field lines in a solar cycle, thus a 0.1% signal. In the program, three polar field lines per 100 active region lines make it to the poles in a cycle: a 3% signal. The reason I have done this is as follows. When I attempted to faithfully mimic the solar magnetic field generation and the self-destruction amounts of magnetic flux, it was difficult to see the large-scale magnetic field structures that the model yielded. It is my belief that much of the active-region field generation and destruction has little relevance to the global motions of the Sun’s large-scale fields. Thus I adjusted the parameters so as to reduce the gratuitous noise.

**Figure 2:**
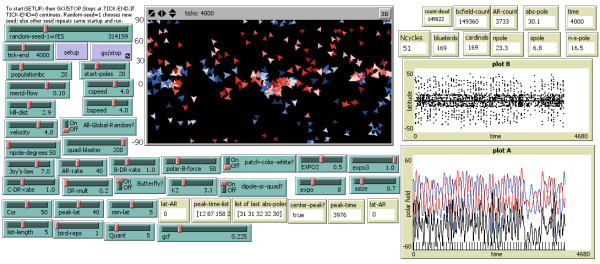
**Same as Figure**1**but extended to a long run of 4,000 tick timesteps.** A quiet period after ~3000 ticks can be seen, reminiscent of a Maunder minimum. One can also see the typical chaotic nature of the solar cycles calculated with this model. One may also discern the Waldmeier anti-correlation of cycle amplitude with cycle duration in Plot A.

**Figure 3:**
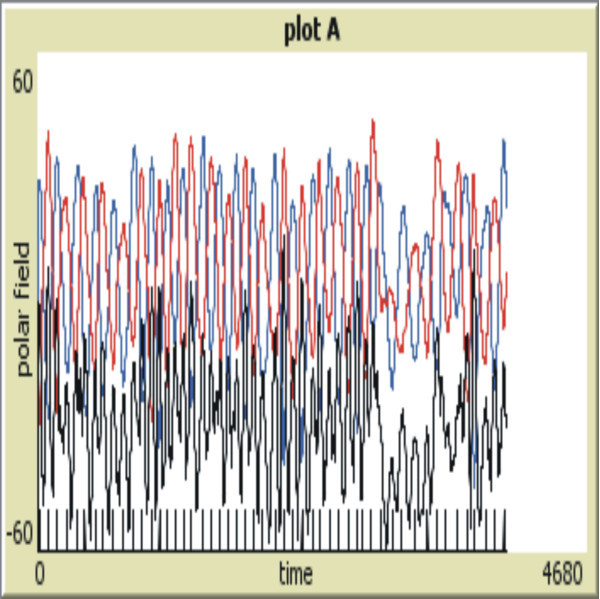
**Plot A from the long run of 4 000*tick*timesteps.** The Blue line shows the north polar field, the south polar field (Red line), and total polar field (Black line) offset by 60 (lowered), to minimize interference with the individual polar fields. This creates the axis at the bottom to be the abscissa of this plot, so that the lowest field magnitudes near 3000 ticks reach near zero value. The individual *tick* marks at the bottom of this plot display the computer calculated polar field maxima. A quiet period at ≈3000 *tick*s can be seen, reminiscent of a Maunder type minimum. One can also see the typical chaotic nature of the solar cycles calculated with this model.

Figure2 shows the model run for an extended period of time of 4 000 *tick*s. Now the model goes through some added idiosyncratic behaviors typical of the Sun. Namely, in addition to the chaotic pattern of irregular solar activity, I see an added feature: the model undertaking an extensive period of weak magnetic activity wherein the polar field almost disappears, three fourths towards the end of the run, located near 3000 *tick*s. Subsequently model activity oscillations recover. This may be considered a foray into a Maunder Minimum-like event. The Plots A and B on the right are difficult to read, owing to their small size in Figure1 The most significant of these plots is plot A in the longer run, hence this plot is shown individually in Figure3. The anti-correlation of amplitude with cycle duration is more readily seen there.

As mentioned, the *tick* unit is an arbitrary Netlogo time unit; however, I associate it with a rough measure in our time units as follows. For the first two cycles, I equate a time period of ≈22 years with 146 *tick*s (from 12 to 158). Hence, I associate a *tick* interval as approximately 55 days, or ≈two Carrington solar rotations (low latitude rotations as seen from Earth). Equivalently, this is ≈ 6.6 *tick*s per terrestrial year. The compression is not an “exact” time unit, for as we shall see, the modeled cycles have varying periodicities (owing to non-linear effects), just as our Sun does, depending upon cycle amplitude. The amplitude - timescale variation is inherent in Babcock’s, Spoerer’s, and Waldmeier’s work. For example, Babcock said it took ≈three years for the field to wind up sufficiently to yield the cycle’s early sunspots, with a latitude variation given by: sin *λ* = ± 1.5/(*n* + 3) where *λ* is the latitude and n is the number of rotations for the solar cycle to reach sufficient amplitude for the magnetic fields to “erupt.” This essentially illustrates the Waldmeier effect wherein larger cycles peak sooner. I have used the term percolation rather than eruption, but, for this model, they are equivalent – some process below active-regions that allows magnetic field to be amplified into sunspots which appear in the photosphere – the existence of magnetic field on the Sun’s surface and its motions and disappearance are all this article cares about. How or why magnetic field appears on the Sun’s disk is not a question this model considers. The entire physics of field generation within the Sun is subsumed by simply generating bipolar magnetic regions proportional the previous minimum's dipole moment. Thus if we simply define a process, called "interior field mechanism" and that this mechanism does two things: 1) it creates random bipoles in the photosphere proportional to the solar dipole moment at the last solar minimum, and 2) it attracts magnetic entities towards opposite signed polar regions, then this seems sufficient to simulate solar cycles, in accordance with this model.

Time units may also be deduced, relative to the differential rotation in the following manner. The Sun’s Equator rotates, in a terrestrial reference frame (synodic) in a period of ≈ 27 days, but our display shows the Equatorial view from the Earth, hence it is a synodic reference frame (rotating in a prograde motion) holding low latitude regions at a fixed, Carrington, longitude. As a consequence, at high latitudes, features on the model rotate towards the left in the display. At about 45 degrees, this amounts to ≈1.6 degrees per day. For two solar rotations, this equates to 90 degrees, and for 8 solar rotations, it is a full 360 degrees. This results in a full 360 degrees (at mid latitudes), thus the field circles once around the poles relative to the Equator, during a period of 225 days. In running the model, one may observe the rotation of features by about this amount, either as individual agents or structures such as opposite polarities near a sector boundary.

The model undertakes different processes sequentially. Nevertheless by following features, one can see large-scale fields forming out of the remnants of old active-regions, namely just as the flotsam and jetsam in an ocean wreckage mark the previous havoc and yield clues to the destruction of a sea going vessel devastated by the ocean. In the case of the fields, they often spread from the active-regions and head towards the opposite poles. These field lines are thought to create the backwards C-shaped structures identified by Bumba and Howard, and studied by the NRL group (e.g. Wang and Sheeley 1994, 2003, and references therein). By running the model, one may observe the model's backwards C shaped structures being created and destroyed. Since each tick mark represents a period of ~55 days, these structures appear and are destroyed rather quickly in the model. One may see the structures best by slowing the speed of the program, by using the slider above the Display, which controls the speed in which the Display is updated. One may note that the full backwards C-shape, often relies upon a degree of N-S symmetry of activity above and below the equator. When there is little N-S symmetry, then one may find only parts of a backwards C-structure exists in one hemisphere: commonly that hemisphere that had most recent levels of higher activity.

The set of parameters chosen for this run of the model may be seen in the bluish slider bars of Figure1. As mentioned, the random-seed slider shows a pseudo random number of 314 159; the number chosen has no significance, except ease of memory. The models run identically for each run with identical starting numbers. However, rather than starting with any other number, if one chooses to start the random-seed with 1, it tells the program to choose a completely different starting random-seed each time. This allows any user to obtain totally different runs each time. If one wants to know the seed-number one must write it out via a line “show random-seed”, and the starting seed will be written in a special line for outputting information, but I haven’t written that feature into the code. At present, the results begin with a different unknown seed each time, unless one specifies the seed.

**Figure 4:**
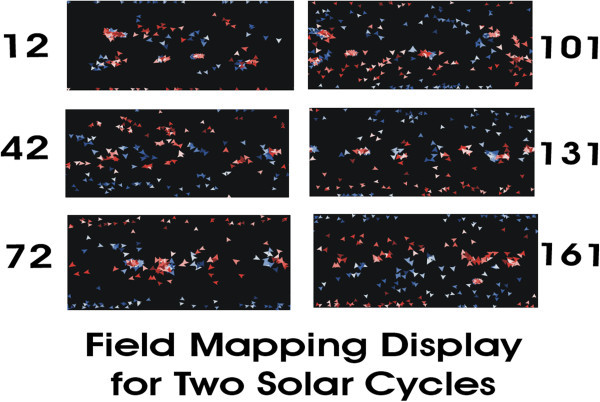
**Shown here are six displays, similar to that shown at the top in Figure**1**, for a number of different timesteps during the first two solar cycles.** Each field entity contains the same amount of magnetic flux; their orientation shows the direction the field is moving relative to the fluid. The arrows are reduced in size at higher latitudes to illustrate the expansion of coordinates in Mercator projections. Each display is the equivalent of a synoptic longitude vs. latitude map on the Sun. The times shown are in program time units, called *tick*s. The displays are Mercator projections taken from ≈two solar cycles (≈22 years), the time period used in Figure1. The model starts with an outward field in the north (blue arrows) and inward field in the south (red arrows). New activity centers first appear at high latitudes in the top left panel. Proceeding downwards, as time passes, activity centers form at lower latitudes, as in the Butterfly diagram. One can see large-scale patterns, similar to UMRs, form as same-sign fields gather together. One can also observe two polar field reversals, also shown in Plot A of Figure 1.

Figure4 shows the field evolution at particular timesteps indicated on the side of each panel, during the first complete solar cycle (equivalent to ≈22 years), the fields evolve to regenerate the same sense field (positive in the North , blue arrows). In the first (top left) panel at a timestep of 12 *tick*s, we see the polar fields, which are initiated at the start of every model run, as well as four new active-region field bursts in each hemisphere, at relatively high active region latitudes. The following fields (those to the left of each emerging bipolar region) have the opposite sign as the polar fields of that hemisphere until the polar fields reverse, in accord with Hale’s laws of sunspot polarity. One is able to see two new bipolar field bursts having a small size in the southern hemisphere. As the bipoles age, the fields spread out; some examples of these are in the northern hemisphere. In the right side of the first panel, one sees the fields separating from the bipoles into the early stages of the fields evolving from BMRs into UMRs.

In the timesteps of 42 and 72, we are able to see a number of aspects of the model's behaviors. For example, we see the following field moving towards the poles at high latitudes. We also see the fields tending to fill the photosphere with a more uniform field magnitude (being less concentrated from their births within high field BMRs). One also sees opposite sign fields moving toward reversing the polar fields. That is, as regions grow, the individual fields are increasingly affected by interactions with the North - South component of the Babcock - Leighton magnetic field. Additionally, one can observe the fields spreading out across the Sun’s surface, as the magnetic elements move towards a more uniform mixture. These are the beginnings of the Bumba and Howard UMR (backwards C-shaped) structures. In running the model, one can watch the model's field lines spread outwards in the directions given by the arrows associated with each field entity. By ≈72 time steps the polar fields have mostly reversed. We also see the low latitude sector-like field patterns below ≈30 degrees (greatly compressed near the Equator). At 101 and 131 timesteps, we see new active-regions and opposite sign polar field with following fields heading towards the poles. This continues until another reversal shown at the bottom right at 161 timesteps, shortly after the second polar field maximum. The displays indicate a higher number of “sectors” per rotation, than the traditional two to four seen during the IMP-1 era in the 1960s. Nevertheless our field maps are only showing calculated photospheric magnetic fields, and such fields DO differ from interplanetary fields. So differences between the number of photospheric UMRs and the number of interplanetary sectors is expected. The potential field source surface model leavens the photospheric fields into the inteplanetary sectors and reduces the number of sectors per rotation.

For the Sun, the poles are the most prominent open-field regions. They serve as special locations in this and other dynamo models where polar field gathers for magnification into toroidal field. This may have simplified our model since these two areas also serves as loci for opposite sign fields to aim towards. The polar regions are the only open-field region this model utilizes with a special or unique preference, to orient field entities, using the Babcock - Leighton field. This, however, was a simplifying modeling choice, not a necessary requirement of this model. The model might be better generalized to utilize any open-field region, rather than the Sun’s poles to serve as the direction which field entities move towards. With this simplifying assumption, field entities are continuously reorienting themselves towards the opposite-sign polar-field region by the B - L force. Some field lines never make it to the opposite signed polar region, owing to their collision with a field entity of opposite sign (on average, for each solar cycle, 97% are destroyed in this way). The field lines may either travel to the opposite polar region, or else be forced to ultimately change directions, when the polar field lines reverse sign. So, aside from reversing direction endlessly, the only real way for a field agent to terminate its existence in this model is for it to run into an opposite sign field entity and then vanish. Note that the field entities we keep track of begin their lives as the leftover field remnants emanating from active-regions; whether such a field has sufficient strength to influence energy transport and thereby affect the Sun’s surface irradiance (e.g. be sunspot-like or facular-like), is an aspect I do not address here. Nevertheless, the interaction of magnetic fields with the convective motions is a much-debated topic of interest to solar physicists.

Figure2 shows the model run for an extended period of time of 4 000 *tick*s. Now the model goes through some added idiosyncratic behaviors typical of the Sun. Namely, in addition to the chaotic pattern of irregular solar activity, we see an added feature: the model undertaking an extensive period of weak magnetic activity wherein the polar magnetic field almost disappears, three fourths towards the end of the run, located near 3000 *tick*s. Subsequently model activity oscillations recover. This may be considered a foray into a Maunder Minimum-like event.

In terms of time units, the *tick* unit relates to a computational step; it is just an arbitrary time interval for our purposes; however, one may associate it with a rough measure of actual time as follows. For the first two cycles, one may equate a time period of ≈22 years, with ~146 *tick*s. Hence, one can associate a *tick* interval as approximately 55 days, or ≈two Carrington solar rotations (low latitudes as seen from Earth). Equivalently, this is ≈ 6.6 *tick*s per terrestrial year. The compression is not an “exact” time unit, for as we shall see, the model’s cycles have varying periodicities, like our Sun does, depending upon cycle amplitude, making a mockery of our terrestrial day, month or year. The amplitude-timescale variation is inherent in Babcock’s, Spoerer’s, and Waldmeier’s work. For example, Babcock said it took ≈ 3 years for the magnetic field to wind up sufficiently to yield the cycle’s early sunspots, with a latitude curve given by: sin *λ* = ± 1.5/(*n* + 3) where *λ* is the latitude and n is the number of rotations for the solar cycle to reach sufficient amplitude for the magnetic fields to “erupt. ” This essentially illustrates the Waldmeier effect wherein larger cycles peak sooner. I sometimes use the term percolation rather than eruption, but they may be considered to be equivalent – some process below active-regions which allows subsurface magnetic field to be amplified into sunspots which appear in the photosphere. The existence of magnetic field in the Sun’s surface, its motions and its disappearance are all this model cares about. How or why magnetic field appears on the Sun’s disk is not a question this model utilizes.

In this model, the Sun’s Equator rotates, in a terrestrial reference frame (synodic), having prograde motion with a period near 27.3 days. The display moves into this synodic rotating reference frame. This holds low latitude features fixed, unless they move relative to this Carrington reference frame. As a consequence, at high North and South latitudes, features on the model rotate towards the left in the display. At about 45 degrees, this amounts to ≈1.6 degrees per day. For two solar rotations, this equates to 90 degrees, and for 8 solar rotations, it is a full 360 degrees. This results in a full 360 degrees (at mid latitudes), thus the magnetic field circles once around the poles relative to the Equator, during a period of 225 Earth-days. In running the model, one may observe the rotation of features by about this amount, either as individual agents or structures such as opposite color agents near a sector boundary.

The set of parameters chosen for this run of the model may be seen in the bluish slider bars of Figure1. The random-seed slider shows a pseudo random # of 314 159; the number chosen has no significance, except ease of memory. The model runs identically for each run with identical starting numbers. Rather than starting with any other number, a starting random-seed of 1 tells the program to choose a different unknown random-seed each time.

In the case of the magnetic field entities emanating from active-regions in our model, they first spread outwards from small bipolar magnetic regions (BMRs) with parameters based upon random number generators. Consequently many field entities are annihilated locally in the early days of an active-region. The field entities slowly change bearings, and are redirected towards the North and South poles, by the magnetic tension associated with the Babcock - Leighton magnetic field.

The magnetic field entities then head away from the locus of the active-region to join larger formations of like-sign field entities into general flows of field magnetism.

The poles are the most common and largest open-field regions. The Polar regions also are unique in their relationship to the Coriolis force. This may have simplified our model since it tends to choose these two areas for opposite sign magnetic fields to aim towards. The Polar regions are the only open-field region this model utilizes, to orient magnetic field entities, using the Babcock - Leighton magnetic field. This, however, is a choice, not a necessary requirement of the model. The model might be better generalized to utilize any open-field region, rather than the Sun’s poles to serve as the direction which magnetic field entities may aim towards. This was one of our simplifying assumptions. Field entities are thus continuously reorienting themselves towards the nearest attractive polar magnetic field region, by the B - L force. Some magnetic field lines never make it to the opposite signed polar region, owing to their collision with a magnetic field entity of opposite sign. The magnetic field lines can either make it to the opposite polar region, or else be forced to ultimately change directions, when the polar magnetic field lines reverse sign. So, aside from reversing direction endlessly, the only real end for a field agent is to run into an opposite sign field agent, in which case, both are annihilated.

I now discuss more detailed aspects of running the model. Examining the interface of Figure1, one sees the following attributes: the Display map is in the top center, and its geometry is discussed later. Plots A and B are off to the right, slider bars, on-off switches, as well as monitors, are all discussed subsequently. To start any run, one must click SETUP, which initiates a few random polar magnetic fields; this prevents division by zero, since averages over the interface are performed. Next, the GO/STOP button starts evolving the model in time. The display situated in the top-middle of the interface show the blue/red magnetic fields as they migrate across the solar disk. The two graphs on the right display average properties of polar magnetic fields (Plot A) and the latitudes of new regions (Plot B) per time step, as the model evolves. Above the Display are a number of generic added features of Netlogo, e.g. a slider bar to speed up the entire processing, however, at high speed the display itself does not keep up with the timesteps, owing to the huge volume of features required to redraw. The field motion calculations are not affected by the speed of the display; all motions are calculated with very high accuracy. Let us now go through the features of our particular solar magnetic field mapping model and its usage in Netlogo.

The coordinate system of the display has the *x* -coordinate as longitude, and the *y*-coordinate as latitude, from −90 to +90 degrees. Each "arrow" represents a "unit" of photospheric magnetic field, 10^23^ Mx per blue or red arrow, and is oriented in the direction the field is moving, relative to the fluid. The blue arrows are outward-directed field and the red, inward directed field, most common in solar displays of magnetic field. A distribution of shades of color, *e.g.* pink to deep red, help to distinguish the individual magnetic fields and their motions. The arrows show the magnetic field motions relative to the fluid. If not directed azimuthally, through local interactions with neighbors, this model does not direct magnetic fields to drift longitudinally beyond the differential rotation flow. The differential rotation flow is most noticeable at higher latitudes due to the slower rotation rates, where magnetic fields of both sign drift to the left. The two graphs on the right of the interface are of magnetic field quantities. Time summaries of the polar fields are shown in plots A and B. Plot A shows the north polar magnetic field (Blue line) and south polar magnetic field (Red line), and total polar magnetic field (Black line) offset by 60 (lower), so as not to interfere with the individual polar fields, and the *tick* marks at the bottom display the computer calculated polar field maxima, defined not to be too small an amplitude, nor too soon from the previous polar field maximum. Polar magnetic field maxima are closely associated with sunspot minima, and these times are chosen, in this model, as the starting point of a new solar cycle. More information on the Interface is available in the Additional file 1. I also discuss the manner in which field entities are born, their annihilation and the forces that guide their motions.

One aspect required an alteration from the movement of agents in Netlogo’s rectangular geometry, as opposed to the Sun’s spherical geometry is the following. Netlogo uses a “turtle geometry,” which means that the agents follow a constant angle in their rectangular space, unless redirected. What this amounts to, is that the agents follow a “rhumb line,” a navigational term that began as sea navigators or captains could draw a straight line on a Mercator map, using a constant “heading” with a straight edge. The rhumb line is also called a loxodrome and has an Archimedean shape; it crosses all meridians of longitude at the same angle, *i. e.* a path derived from a constant bearing determined by its initial value. Although our agents interact with numerous other agents; in their absence, they should ideally follow a great circle, rather than the rhumb line. As a consequence, I have redirected the agents so that, unless acted upon by other forces, they follow a great circle path rather than the loxodrome. This is done with algorithmic routines in the program, red-Great-Circle-Force and blue-Great-Circle-Force that may puzzle anyone who wonders what that routine does. Most others are fairly straightforward.

As discussed earlier, Alfvén’s frozen-field approximation applies to regions of high conductivity; it ensures that the movements of the magnetic field and flow of the conducting fluid are linked together. This concordance can occur in different ways, depending upon the relative strength of the field and fluid: either the field is sufficiently strong to enforce that the fluid travels along it, or the fluid motions are sufficiently strong so that the field is distended by the flow. In this latter case, the flow can strengthen the magnetic field by stretching or extending it. In such cases, the flow pulls against the field, thereby lengthening it, and the field therein gains energy.

For the current model, I take the steady-state motions to consist of the meridional flow and the differential rotation. The present model predominantly deals with spatial and temporal differences from these steady-state solar motions. The field motions the program calculates are not inconsistent with Alfvén’s frozen field approximation; the field motions are calculated with small temporal and spatial flows, relative to the predominant large-scale background flows. Differences from the background flows may occur when magnetic field enters the picture, and it is only through the mutual interaction of field and flow that non-zero field and non-zero flow differences are thought to arise. The model calculates that the field and flow have a tendency to return to “normal,” that is back to their undisturbed states (e.g. zero flow relative to the local flow). In this model, the field and flow tend towards being equalized or “homogenized,” namely having uniform values, so fields tend to move from high field magnitude regions to low field magnitude regions. Further details of how this is done and the rationale are available in Additional file 1.

As mentioned, the disappearance of magnetic field has an important role in this program’s ability to limit the quantity of magnetic field. A corresponding, but real, process may similarly help the actual Sun, by preventing the magnetic field on the Sun’s surface from growing too large. In this model, if the global field grows larger, then more and more agents of opposite signs annihilate and the overall field is reduced. Thus an upper limit on the model’s global field occurs. Perhaps for the Sun too, this may be one stabilizing factor that limits the overall level of the Sun’s surface fields. Naturally, physical aspects such as plasma pressure and conductivities play important limiting roles too.

### Model utilization overview

Here I describe two model runs, and general aspects of the solar magnetic field mapping model’s behavior. I begin by discussing some aspects of our nominal model shown in Figures 1, 2, and 3. Still figures do not do justice to the model; it is desirable to view the model running so that one can see the field motions; two movies: movie1 and movie2 allow the program’s display to be seen running. They require Quicktime to show the movie: http://www.apple.com/quicktime/. Additionally, “Additional file 1,” before the appendix allows the model to be downloaded and run, or by using the Netlogo linkage here: http://ccl.northwestern.edu/netlogo/models/community/Solar%20Field%20Mapping%201p07.

In Alfvén’s frozen-field approximation, for plasmas of high conductivity, flows and field lines move together. In the photosphere, one readily observes the motion of small field regions moving at close to the surface differential rotation rate in which the fields are embedded. Our model utilizes this behavior by advecting the field along with the two prominent fluid motions: the differential rotation, and meridional circulation. Our model, however, allows for temporal and spatial variations of the field and flow to move relative to these globally averaged motions. Because of the conducting nature of the photospheric gases, blocks of photospheric plasma may engage in motions so that currents flow, thereby allowing the outward magnetic field to preferentially lower its global energy.

The main display coordinates are in a frame of reference such that the Equator appears stationary. Hence high latitude fluid and field drift to the left owing to the Sun’s differential rotation. Meridional flows advect field towards the poles very slowly. There are sliders which control many parameters. In addition to the surface field advection, this model emphasizes particularly, the motions of field relative to the fluid. It is this unique aspect that has been considered particularly, because the relative motion is important to field transport. If all magnetic fields, regardless of strength and orientation, were to move identically, one could not generate field magnification. I make the following assumptions to control the direction of field motions. Each field line is an “agent” born with an initial location and velocity chosen from a distribution controlled by random number generators in accordance with the general behavior of Hale’s and Joy’s laws, as well as Spoerer’s Butterfly diagram. These aspects do not introduce any inherent field separation (the agents are initially directed isotropically on the surface of the Sun). of sunspot polarities, On the small scale, one may view the motions of individual fields relative to their neighbors behaving as individual entities, governed by local properties. On the large-scale, large range forces and fluid motions predominate.

Field agents reorient their directions based upon these two overarching aspects: long-range and short-range forces. In this model magnetic fields operate by reorientation of the agents, but not their speed, relative to the fluid. The long-range forces are actually local forces, but with “knowledge” gained by more distant properties. For example, one major effect is from the Babcock-Leighton field, hypothesized to pull on photospheric field entities towards the opposite signed pole. This force is ascertained by the polar field values at the previous solar minimum. A secondary long-range field force is basically another local one: that each agent changes its direction towards regions of lowered magnetic field strength. This is based upon the thermodynamic viewpoint. The magnetic field in a fluid is an “intensive” thermodynamic variable, as are temperature, pressure, and chemical potential. Fluids tend to move toward equalization of intensive variables (*e.g.* fluids with disparate temperatures transport energy from regions of high temperature to regions of low temperature), which is why these variables tend to smooth with time. This provides a guide for this model. From this viewpoint, if one imagines a photosphere filled with various sources of magnetic field, the field would tend to equalize the magnetic field magnitude everywhere.

The short-range forces are controlled by the agent’s nearest neighbors. Each agent examines SAME and OPPOSITE color neighbors. Agents of the SAME color tend to flock together, as in the Boids Starlogo Bird flocking model (see http://education.mit.edu/starlogo/samples/boids.htm), or similar fish-schooling models. Group behaviors are accomplished by adjusting individual directional adjustments towards the orientation that their nearest neighbors are directed towards. The amount of short-range adjustments are set and controlled by parameters which have been tuned and adjusted in the software. One aspect becomes apparent when we see the model run. The long-range magnetic force attracts or draws "following field" to the poles and "preceding field" to the opposite pole. The strength of this force is controlled by the polar-B-force slider. A value of 0 will drive this force to zero. Despite our use of Joy’s law in this model, it has relatively little influence, compared with the polar-B-force term. It is unclear which dynamo models actually use the powerful Babcock-Leighton tension force that seems to dominate our model, but it is unfathomable to this author why it seems to play a tiny role in other magnetic dynamo models.

The current model, discussed fully here, has also been able to mimic a number of solar phenomena successfully (Schatten, 2012b): the Solar Cycle (11 year) Oscillations, the Waldmeier effect, Unipolar Magnetic Regions/Sectors/Coronal Holes, Maunder Minima, and the March/Rush to the Poles involving the geometry of magnetic field reversals.

### Summary

I develop a 2D cellular automata algorithmic model using Netlogo as the agent-based programming language. The agents come in two distinct breeds: red and blue agents, each member of the former set contains 10^23^ Mx of inward directed magnetic field, and the latter, an equal amount of outward directed magnetic field. The agents are displayed as arrows marking the direction they move relative to the bulk fluid motions, the well known meridional circulation and differential rotation. Our model deals with spatial and temporal differences from these steady fluid motions. Put simply, our algorithmic model calculates agent motions as follows. Agents are born in low-latitude active-regions through a random number generator, proportional to the polar field strength at the previous solar maximum, consistent with Hale’s and other statistical laws governing sunspot appearances. The magnetic field entities in this model move about on the solar surface by a number of flows and forces I describe, and eventually are annihilated when two opposite color agents come together in close contact. The model displays field patterns similar to the UMR patterns found by Bumba and Howard. The reasons for, and the model’s usage of, these patterns is more fully discussed in Appendix 1.

There are but a few effects agents have on other agents: i) while at the poles, field agents are connected via the Babcock - Leighton (B - L) subsurface field to lower latitudes which allows them to undertake two duties there: A) the B - L subsurface field spawns the next generation of new magnetic field, and B) the B - L subsurface field attracts lower-latitude fields via its magnetic tension, acting locally; ii) nearby agents affect each other’s motion through short-range interactions; and iii) through annihilation: when opposite field agents get too close to each other, they disappear in pairs.

Our algorithmic model considers all effects as local. We simply place some in the long-range category and others in the short-range category. Forces placed into the long-range category are chosen based upon entities in the model obtaining information gleaned from distant entities. The predominant one is that associated with the magnetic tension from the “Babcock - Leighton” subsurface magnetic field, often pictured to wind its way from one pole to the opposite pole, and occasionally popping up through the photosphere. The strength of this force is simply ascertained by the distant polar field magnitude. It is assumed to pull on all field entities in our model. The force results from the differences in magnetic tension from the subsurface field and the field which emanates from the entity through the photosphere. We consider that once fields exit the Sun, the field weakens so drastically, as the field splays outward into the corona, that the outer magnetic tension can be neglected. Hence magnetic entities are pulled along the B-L fieldline toward the poles with the opposite sense field direction.

The short-range forces are defined as those between close neighbors: they guide field entities in the following manner: they encourage like-field entities ( members of the blue breed) to travel together, and unlike entities (opposite color breed members) to repel each other. Such forces are in direct opposition to the vacuum Maxwell stress tensor! Many physicists would take umbrage at this suggestion with such ferocity that it would not be considered at all. Nevertheless, we would parry that it is not the basic laws of Maxwell that we are tampering with, but rather the motions of field entities in a convective environment. It is this convective environment that allows the surface of the Sun to be mottled with dark sunspots and bright faculae, rather than contain a uniform field and shine with a uniform glow all over its surface. It is these variations that allow the Sun to shed its luminosity more efficiently through the creation of enhanced differences. These differences can grow in a convective environment, when magnetic fields inhibit the efficient mixing of surface materials, allowing structural differences to grow. One of the assumptions of thermodynamics is thermal equilibrium. Clearly this is not applicable to the photosphere of the Sun, particularly when magnetic fields are present, allowing thermal inhomogeneities to be expressed! Much of this comes from Zwaan’s (1985) ideas of convective collapse.

This model appears to display a dynamo type behavior for two reasons: 1) The transport of sunspots into the photosphere is invoked through a process outside the domain of this model, by having new field regions injected into the photosphere thereby allowing a positive feedback loop to be created and 2) the motion of magnetic fields of opposite sign are directed towards opposite poles, owing to the long-range Babcock - Leighton (B - L) subsurface field force, which may not have been utilized in the same manner that this model does. This second influence allows enormous amounts of solar magnetic flux to travel from one pole to the other, allowing a reversal of the Sun’s polar fields most every cycle, through opposing motions of opposite sign field agents. Longitudinal asymmetry, for example, allowing oppositely directed magnetic flux motions to exist at the same latitude on different longitudes clearly can aid a north-south dipole to flip over.

## Appendix 1 methodology of magnetic field mapping model

Here I predominantly discuss this model’s methodology for directing field line motions in the photosphere. I break this up this into a number of modeling aspects:

1. The model’s use of locality. What is it, what does it mean, and how is it used?
  We discuss this after this listing;
2. The application of the Alfvén frozen field approximation, with a time-dependent flow in the photosphere. This relates to applying a consistency of fluid motions together with magnetic flux transport; and
3. Reasons for utilizing the methods we do to guide magnetic field entities in various directions, in particular towards regions of reduced field magnitude. This also concerns aspects related to the field motions and the context within the solar photosphere we predominantly observe, and the relationship with inner and outer boundary conditions.

Let us consider the above items in order, proceeding as we go. Concerning locality, this means that entities are affected only by entities in their local neighborhood or environment. That is, there is no spooky “action at a distance,” as exists in quantum mechanics, and even existed in Newtonian mechanics, with gravity of the Sun affecting planets, with no clear reason why. As mentioned, it exists in quantum mechanics, but this is not easily understood, which is where the “spooky” part comes from, with all the conundrums in that subject. In the current model, although we state we have locality, nevertheless, the model does use data from distant regions. For example, the model uses polar field data to calculate the Babcock-Leighton force, relying on the amount of magnetic field in the polar regions. How is this considered local? Well, it is considered local, because the polar magnetic field winds its way within the Sun, either in a deep or in a shallow pattern, but it pulls on surface field entities with its magnetic tension in what amounts to an “inner boundary condition” and thus does act locally. Virtually all the model’s forces are local in this regard. They sometimes get their information from distant sources, as the B-L field is a subsurface field connecting the poles with lower latitude fields. Many of the forces about nearest neighbors, and field entity death are clearly local.

Concerning item ii, this shall require much more discussion than i. Following this, we shall then go onto item iii. We recognize that the photosphere has uniqueness in being the boundary between the optically thick regions below, and the optically thin regions above. What flow structure would a field line obey, if moved about in a highly conducting fluid such as the photosphere with its high plasma β? In such an environment, Alfvén’s frozen field approximation is highly regarded. Yet sometimes it is thought to imply that the fields and flows are parallel. This has sometimes been considered to be the implication of the frozen field approximation. There is no question that below the photosphere, even with its low ionization fraction, the conductivity is sufficiently high, that on large-scale sizes, Alfvén’s frozen field approximation remains dominant. We shall try to understand its implications.

One problem is that sometimes there appears to be a disconnection involving the utilization of Alfvén’s frozen field approximation. The approximation is often taken to mean that field and flow are everywhere parallel, namely that both are directed together, with one being a scalar multiple of the other. This could not be less true. It is this conclusion that goes beyond what the actual approximation asserts. If both fields were everywhere parallel, then both vector fields would be identical, except for a common factor, relating the two. This is an area of mathematics that is well understood within the field of linear algebra. When such a situation arises, namely having two vector fields with a linear factor relating the two, the fields are called “isomorphic,” meaning that the two fields are “essentially the same.” Yet one of the first uses of Alfvén’s frozen field approximation by Parker (1958) illustrated the total lack of isomorphic behavior. His early theory of the interplanetary magnetic field (IMF) strongly suggested that the IMF lies on an Archimedean spiral, while the fluid flow of the solar wind is approximately radially outwards. Within a decade, the Archimedes field geometry and the radially flowing solar wind were observed. The two fields were disparate, and did not simply bear an isomorphic relationship to each other. Parker’s use of the simple pattern of a rotating water sprinkler served to illustrate the “garden hose geometry.” Hence there is no isomorphic behavior between the vector magnetic field and flow velocity. Hence this illustrates that one has to be very careful not to misuse Alfvén’s approximation. There may be other mistakes involved, however, to this author, the common mistake people make of relating fluid motions, is lack of understanding is the difference between streamlines and streaklines.

It is best to first understand the more basic fluid concepts. The simplest is that of fluid pathlines; which are the paths that individual elements would follow, as they move through space. Streamlines are the family of curves that are instantaneously tangent to the flow velocity at any point in time. Lastly, streaklines are the locus of points of all the fluid particles that pass through a particular special point in the past. This last aspect has the most relevance to the Alfvén’s approximation, since it is the only one that would render an Archimedes spiral pattern in the solar wind. Let us see how. Tracers, such as the magnetic field, use streaklines. The field simply follows the flow including its temporal variations. Hence the frozen field approximation (in the region below the photosphere) seems to relate to field configurations in space generated flows. What is the nature of streaklines? These streaklines are defined to be the locus of points passing through a given point in space-time. Thus, they serve to form as the basis for the magnetic geometry in a high conductivity medium, like the Sun, where a time-dependant flow occurs. Another example will help clarify the difference between the streakline and the streamline. The most obvious example is the pattern of smoke issuing forth from a smokestack carried by a time-varying wind. Another example allowing a time dependent motion, such as the rotation of the Sun as plasma (e.g. the solar wind) issued forth, would be the pattern of smoke from the old sky-writing planes.

What flow structure would a field line obey, if moved about in a highly conducting fluid such as the solar atmosphere, particularly those regions of high plasma β, such as the photosphere and below, where aside from sunspots the convective fluid is thought to push the magnetic field around with impunity. In such a case, Alfvén’s frozen field approximation is highly regarded. The only question that is, to this author, misunderstood, is that people often take this to indicate the field follows a streamline. I hope to convince the reader that a more accurate term is the fluid dynamicist’s streakline that the field should be following. It is a subtle point, but an important one, when a time-dependant flow is involved. In particular, streaklines differ from streamlines, as they allow any time-dependency in the source, either associated with a magnetic field, or other aspect connecting moving points in space-time.

For the interplanetary field, the corona issues out of the Sun’s upper atmosphere, above a source surface (Schatten et al. 1969) and acts as a tracer of the flow, hence the field moves in a streakline, remaining “attached to one point which issues the field forth into space. The particles, however, move in pathlines, more usually understood, if called by the non-fluid term the trajectory of the particles. Schatten (2001) outlines the differences of these patterns, and provides illustrations, with formulae and calculations showing the differences. The pathline is generally well understood, one just follows the path/trajectory of the particles moving forwards in time. What is not generally understood, except by fluid dynamicists, is the streakline; often this is misunderstood and taken to be the time independent streamline, however, this is only the case for time independent flow. The streakline is a more general concept applicable in time dependent flows.

Let us briefly outline the various definitions and differences between fluid trajectories and tracer trajectories (which is the category a magnetic field would fall under, given Alfvén’s approximation). The reader who wishes to calculate these, can read Schatten (2001). Lines which describe various aspects of the fluid flow, generically all these are called “ribbons” of the flow, may be described mathematically as follows: a) streamlines are ribbons with vectors parallel to the instantaneous flow field at a given time, *t*_0_: *dx*^1^ : *dx*^2^ : *dx*^3^ = *x*^1^ : *x*^2^ : *x*^2^; b) pathlines or trajectories are the lines in space traversed by a fluid particle at , representing an integral of the equation of motion of the fluid particle over time: , − *∞* < *t* < *∞* ; and iii) streaklines are obtained by an “inversion” of the equation of motion: given the fluid motion:  of a point , this equation is first inverted to obtain the subsequent location  of any point through an earlier position in space, , resulting in:  hence the streakline through  at any time t, is given by:A.1

For example, I wish to find the magnetic field through a point on the solar surface, I shall call footpoint A, which may move as it rotates or otherwise moves on the solar surface. Given that the field follows a streakline, then this is the locus of points in space and time, which when integrated backwards along the velocity field yields the desired location on that moving footpoint A.

Now I return to item iii in the opening paragraph of this Appendix: reasons behind choosing field entities to move towards reduced field magnitude environs. Let us step back, first, and give a little flavor for the photospheric environment in which the fields are embedded. The photospheric gasses have densities similar to the Earth’s ionosphere, with temperatures near 6000 K, and with magnetic fields varying from near 0 to values near a kilogauss, and are generally oriented radially, although active region sunspots often do show some tilt. The photospheric material thus contains a wide variety of plasma β values. Nevertheless, considering the stiffness of the field as it opens into the solar corona, it often tends towards a force-free field configuration. Of course, the existence of flares illustrates that there exists some hysteresis in the system, namely for intervals of time, the solar field can, and does, form a degree of non-potentiality, until the structures get too “bent out of shape,” and then dramatically change their connectivity, so as to allow fields to move towards lowered energy states. Hence the coronal field can readjust itself towards new potential configurations, while at the same time allowing different solar latitudes, with different rotation rates, to stay connected with each other. The work of Sheeley et al. (1987) and Fisk et al. (1999) show how the differential rotation can exist on the Sun yet have specific periodicities (rigidly rotating patterns in the corona). An interesting similarity, involving the presence of rigidly rotating structures within smoothly varying motions, exists in motions within the rings of Saturn. While having the majority of dust circling this marvelously decorated planet in Keplerian orbits, Voyager I saw rigidly rotating, spoke-like dark lanes. These were mysterious when first viewed by the Voyager spacecraft, until it was recognized that just outside and inside the various ring bands, there were small satellites which served to shepherd the orbiting dust particles, and could provide a basis for the rigidly rotating pattern of structures. We now make the case for why we choose the modeled photospheric field motions to be directed towards regions of weaker field magnitude.

Let us first say that in short, the motions we choose amounts to moving the field entities towards a constant field magnitude, for a Sun filled with magnetic entities of both signs, as required by Maxwell’s divergence free equation. In terms of thermodynamics’ intensive variables (see Morse, 1969), such as temperature and pressure, creating equipartition, for magnetic fields, this equates to magnetic field values moving towards uniform field magnitude. An analogy may help to explain this heuristic approach. In the Sun’s corona, the potential behavior of magnetic fields allow the coronal fields to play a governance role in how the photospheric fields evolve, owing to the fact that it is predominantly through the release of an “over winding” of the fields when they are attached to different latitudes, with different rotation rates, that the excess energy needs to be released (predominantly through coronal mass ejections and flares). Just as rivers flow towards the lowest altitude, sea level, they can find, and this allows the oceans to serve as an equalizer of potential energy, the near vacuum of the Sun’s corona allows a relatively free unwinding of the photospheric fields. In any case, the potential field models are not perfect, but on average are able to model the quiet corona reasonably well, on average.

For the present case, the complexity of the coronal field is not needed, as we are simply trying to understand how fields might move from their source in newly born active regions to their demise in weaker field regions. Hence we consider the energy associated with a photospheric magnetic field a small distance, say h < R, above the photosphere. In this case, each element of field, of a given small area, Δ**A**, and height, h, has an energy approximated by: , where **B** is the local field strength. I have chosen this model to obey rules having field motions such that they will minimize the total magnetic energy, of all magnetized regions in the Sun’s surface. In the case of the solar fields, allowing the corona to move towards a lowered energy state, within the volumetric areas that the photospheric fields connect to, leads the surface field to having a ≈constant field magnitude over the Sun’ surface, as we discuss. If one is not convinced by the author’s reasoning, s/he may find comfort from the observations by (Bumba and Howard 1965), who found Unipolar Magnetic Regions on the Sun, essentially having the same field behavior and geometry as our field modeling yields, but of course Sheeley et al. (1987) also found their model gave results with a quite different model.

This unique aspect of our model is particularly striking. The model’s behavior is illustrated by examining the magnetic field becoming more uniform in the following “experiment.” One may run the model with its effective “diffusion coefficient” being decreased; this may be undertaken as follows. One varies the *kill-dist* parameter, set originally at 2.9. The product of *kill-dist* and *velocity*, has the units of, and acts like a diffusion constant, in turbulence, as in Leighton’s model. Although this model is not diffusive, the product of these parameters behaves somewhat similarly to diffusion with a diffusion coefficient, K. One may examine the model’s behavior with enhanced diffusion, say *kill-dist* = 6, and watch how weak the fields become by annihilating each other off more readily. Now try lowering *kill-dist* gradually towards ≈ 1; see how the fields behave more like a fluid, and how many entities the model can run simultaneously! The surface of the Sun becomes highly saturated with field entities. Then try lower values, say 0. 5; watch the Sun fill with a nearly uniform field magnitude and this will aid understanding the workings of this model, despite the fact that the flows will be totally swamped, in a seeming quagmire of field, one can readily see that the field density is tends to become more uniform than in the low-K cases. Namely the model is trying to equalize the magnetic pressure, by having a constant H and thus the model moves towards a constant field magnitude, 〈|*B*_*m*_|〉, over all elements of the surface, defined by:A2

Incidentally, there is no “gridding” in the Netlogo^*TM*^ program; the display has grid markers, but they do not have anything to do with the entity’s motions; they are simply a coordinate system, and the model can vary its accuracy as needed, so it handles cases of low-K quite well! This is one, amongst many of Netlogo^*TM*^’s remarkable abilities to undertake cellular automata calculations.

The energy is found by summing the product of the potential with the magnetic elements on the surface, much as the potential energy in a gravitational field or the electrostatic energy of a charged sphere. With the magnetic field obtained by the mean absolute value, the energy is then given by:A3

where *R*_*S*_ is the solar radius. How can one impart motions to the magnetic fields, so that they move towards regions that lower the total energy, **E**? This model simply reorients fields, in addition to the other motions discussed, towards weaker field regions. In examining runs of the model, one can readily observe fields moving towards weaker field regions.

This behavior, of magnetic entities going from high field magnitude locations to lower field magnitude locations is well known, perhaps so commonly, that it is not even realized. One sees it in the fact that active regions decay in a few days. It is seen in the Leighton dynamo model, where diffusion is taken as a major element of the model. This is a mechanism that transports field from high field regions to lower field regions. The present author is simply more in favor of directed flows rather than diffusion, since he is attempting to develop a physical model, rather than a statistical one. The example in the article, at the beginning of Section 2, discussed as Scenario 1, involving a single bipolar group moving towards a reduced energy state consisting of two hemispheric opposite field patterns illustrated how motions towards lowest energy states may be obtained in a simple fashion, namely moving field so as to attain constant field magnitude over a surface.

This unique aspect of our model is particularly striking. The model’s behavior is illustrated by examining the magnetic field becoming more uniform in the following “experiment.” One may run the model with its effective “diffusion coefficient” being decreased; this may be undertaken as follows. One varies the *kill-dist* parameter, set originally at 2.9 The product of *kill-dist* and *velocity*, has the units of, and acts like a diffusion constant, in turbulence, as in Leighton’s model. Although this model is not diffusive, the product of these parameters behaves somewhat similarly to diffusion with a diffusion coefficient, K. One may examine the model’s behavior with enhanced diffusion, say *kill-dist* = 6, and watch how weak the fields become by annihilating each other off more readily. Now try lowering *kill-dist*.

Now try lowering kill-dist gradually towards ≈ 1; see how the fields behave more like a fluid, and how many entities the model can run simultaneously! The surface of the Sun becomes highly saturated with field entities. Then try lower values, say 0. 5; watch the Sun fill with a nearly uniform field magnitude and this will aid understanding the workings of this model, despite the fact that the flows will be totally swamped, in a seeming quagmire of field, one can readily see that the field density is tends to become more uniform than in the low-K cases. Namely the model is trying to equalize the magnetic pressure, by having a constant H and thus the model moves towards constant 〈|*B*_*m*_|〉. Incidentally, there is no “gridding” in the Netlogo program; the display has grid markers, but they do not have anything to do with the distance calculations; they are simply a coordinate system. The field entities’ motions are calculated to very high accuracy, and the program can vary its accuracy as needed, so it shandles cases of low-K quite well! This is one, amongst many of Netlogo’s abilities to undertake cellular automata calculations.

## Electronic supplementary material

Additional file 1: Movie 1: Shown is the Display section of the Solar Field Mapping model’s Interface vs. time for about 2 solar cycles. The movie can be viewed with Apple’s Quicktime movie viewer. It was made with a model identical to the model described in this paper, but written so as to allow movies to be made into a mov file. The movie can be stopped, or played at a slower speed with the Quicktime movie viewer. **Movie 2.** Similar to movie 1, however, the time period of the movie is extended; this allows one to see many solar cycles in the Display mode. (ZIP 2 MB)
