# Supplementary material for: Large-scale solar magnetic field mapping: I
Source: Springerplus. 2013 Jan 23;2:21. doi: 10.1186/2193-1801-2-21 (PMC3601253; doi:10.1186/2193-1801-2-21)
Supplement: Supplementary file 1 — Additional file 1: Movie 1: Shown is the Display section of the Solar Field Mapping model’s Interface vs. time for about 2 solar cycles. The movie can be viewed with Apple’s Quicktime movie viewer. It was made with a model identical to the model described in this paper, but written so as to allow movies to be made into a mov file. The movie can be stopped, or played at a slower speed with the Quicktime movie viewer. Movie 2. Similar to movie 1, however, the time period of the movie is extended; this allows one to see many solar cycles in the Display mode. (ZIP 2 MB) [file 40064_2012_134_MOESM1_ESM.zip › Solar Field Mapping 1p07.html]

Solar Field Mapping 1p07


This page was automatically generated by NetLogo 4.1.2.

The applet requires Java 5 or higher.
Java must be enabled in your browser settings.
Mac users must have Mac OS X 10.4 or higher.
Windows and Linux users may obtain the latest Java from
Sun's Java site.

---

In order for this to work, this file, your model file
(Solar Field Mapping 1p07.nlogo), and the file NetLogoLite.jar
must all be in the same directory. (You can copy NetLogoLite.jar
from the directory where you installed NetLogo.)

On some systems, you can test the applet locally on your computer
before uploading it to a web server. It doesn't work on all systems,
though, so if it doesn't work from your hard drive, please try
uploading it to a web server.

You don't need to include everything in this file in your page.
If you want, you can just take the HTML code beginning with
<applet> and ending with </applet>, and paste it into any HTML
file you want. It's even OK to put multiple <applet> tags
on a single page.

If NetLogoLite.jar and your model are in different
directories, you must modify the archive= and value= lines
in the HTML code to point to their actual locations.
(For example, if you have multiple applets in different
directories on the same web server, you may want to put
a single copy of NetLogoLite.jar in one central place and
change the archive= lines of all the HTML files to point
to that one central copy. This will save disk space for
you and download time for your users.)

powered by
NetLogo

view/download model file:
Solar Field Mapping 1p07.nlogo

## HELP

To start the program, click SETUP, and then GO/STOP. One may vary the parameters of the run via the various slider bars. One of the most important aspects is next to the SETUP AND GO/STOP buttons, labeled tick-end. This helps control the length of the time the model can run. The value can be set from 0 to 2000; if run at 0, the model doesn't NORMALLY stop. One may stop the model nevertheless, by clicking the GO/STOP BUTTON.

There are various inputs, mostly with slider bars, and some with on-off switches. All can be left as is, or one can play with varying some. They are described in greater detail below. Output in monitors provides output numbers; the large display shows magnetic fields on the surface of the Sun, and in the right, a graph of polar field time variations (for the Sun, the normal convention is blue is positive=outward field, and red is negative=inward field). We also call them, based upon these colors, bluebirds and cardinals. The shadings of red and blue mean nothing, just allowing one to track the birds more readily. These field motions portray how the Sun's magnetic dynamo is "beating", like the Sun's heart beat; of course for the Sun, we see the surface (photosphere) best. Sunspots are very much like the low latitude bursts of field, seen in the display diagram. They occur predominantly at low latitudes. The time-scale for the Sun is very different from our usual time periods. The Sun rotates in about a month, at low latitudes, and each "tick" time unit then corresponds to just under 2 solar rotations.

A slider bar at the very top, allows the SPEED of the model to increase or slow down. To stop or start the model, press GO/STOP again.

  

## WHAT IS IT?

This solar field mapping model is the work of Ken Schatten, a solar physicist; he may be contacted about model at: KSchatten AT Alum Dot MIT Dot Edu or kennuth1 AT comcast Dot Net. The version can be identified as model# 1p03, as in 1.03, but with the p meaning . .

This is a model which attempts to mimic the motions of "large-scale" magnetic field lines on the surface of the Sun, also known as the photosphere. Solar Field Motions, poorly understood, nevertheless give rise to the solar dynamo, hence sunspots, flares, the Earth's Aurora, and some exotic terrestrial effects known as Space Weather, affecting satellites and power grids at times when specific events happen.

The motion of magnetic fields as they cross the solar disk is poorly understood, yet it seems to play a role in the solar dynamo, as polar fields are often used to predict the Sun's activity. Thus this represents a puzzle: how can the surface fields play a role in the solar dynamo, seemingly rooted in the deep interior? We note that when magnetic fields get to the Sun�s poles they serve as seeds for the next solar cycle. This model depicts what we know about these puzzling processes, yet our knowledge is incomplete.  
The photospheric field patterns seen oscillating in the display show a semi-regularly manner. They do so in accord with the so-called "Solar Cycle." This oscillatory behavior, sometimes is also called the "Solar Dynamo," since it generates fields in "active regions" very much like a power plant generates electricity. Active Regions (ARs) show new burst of fields lines most often at low latitudes (< 45 degrees latitude). A field line birth, like a baby's, occurs rather quickly. For the Sun, this is often less than a day to a few days, much less than a "tick unit" (the ~50 day timescale of each step in this model). In this model, we do not see sunspots per se, just the active regions (the areas where they are born) and the magnetic field regions they leave behind. These are so-called Bipolar Magnetic Regions (BMRs), which most often are sunspot groups in "pairs" or larger groupings of both sign sunspots (inward - shown in red, and outward shown in blue). We call the red ones cardinals, and the blue ones bluebirds, for continuity with other Netlogo models (see Reynolds' Boids model http://www.red3d.com/cwr/boids/). Sunspot fields often cancel or dissolve below the Sun's surface rather quickly (in a few days); this program concentrates on the longest lived fields; these are the remnants of active regions. Technically, they are called faculae (latin for torches) or plage (French for beach), since these regions appear bright against the Sun's visible disk, particularly near the Sun's limbs.

The remnants of these active regions gather towards the Sun's poles and this forms the solar cycle, we discussed earlier. They are the longest-lived semi-regular solar patterns, although they also group in some grander events (called Maunder-type Minima, lasting about 50-100 years or longer), but these are less well understood. In solar cycles, the polar fields reverse most often with a period near ~10-11 years. So periods   
have been seen as short as 8, and some as long as 17 years. Since it takes two reversals of field sign for the polar field to return to its original direction, a solar cycle sometimes is said to have a period of 20-22 years. Our model uses a Netlogo "tick" time unit; which for the current model and parameters corresponds to ~45-50 days. This is the length of time it takes the Sun's equator, as seen from the Earth, to rotate almost two times around on its axis. Thus it is a rather coarse unit, suited for long-term solar changes, as opposed to rapidly varying active regions. This is why we see the high latitude features in the model rotating rapidly to the left. They rotate more slowly than the Sun's equator, thus appear to move "backwards," (since the Sun is rotating relative to the fixed stars from Left to Right, as viewed from the Northern terrestrial hemisphere. This perspective is similar to the appearance of a station platform from a train appears to move "backwards" from the relative perspective of (a person on)a forward moving train.

  

## GENERAL INFORMATION ABOUT THE MAP DISPLAY

The model displays motions of fields in the Sun's photosphere (or visible surface), in a rectangular projection, ( e.g. the typical Mercator map of the Earth is one showing continents, which expands the high latitudes excessively; for the Sun, modern maps are called synoptic maps, which are equal area projections). Because of this, the size of the arrows is reduced in accordance with the changing area with latitude.

The starting state of the model consists of a number, start-poles, polar field lines. One may consider these as remnants from some preceding solar cycle. They emanated from low latitude structures, often called activity centers, or sunspot groups or bipolar magnetic regions (BMRs). The model DOES NOT show the very intense sunspot fields, but rather the weaker magnetic fields that emanate from these magnetic regions: called faculae, plage and network structures. Magnetic elements either point out of, or into, the Sun. We color these two elements Blue and Red, and refer to them as BLUEBIRDS and CARDINALS. We use these bird names, as our model is an offshoot of the bird flocking cellular automata model (Boyds) by Craig Reynolds. The large DISPLAY shows these field structures as blue and red arrows in a mercator-type plot of the Sun's surface fields (as calculated by this model with pseudo-random data, rather than the Sun's actually observed fields). These fields travel around the solar disk, in a rather chaotic manner, but somehow end their life globally predominantly at the Sun's poles, which this model shows. The fields also die in, and outside active regions in weak field regions called unipolar magnetic regions (UMRs).

The coordinate system of the display has the x-coordinate as longitude, and the y-coordinate as latitude, from -90 to +90 degrees. Each bird or arrow shown represents a "unit" of photospheric magnetic flux, and is oriented in the direction the flux is moving, relative to the fluid. The units of flux are ~10^23 Mx, per blue or red arrow, with one Maxwell equivalent to 1 Gauss cm^2. Sometimes in this model, we refer to each blue arrow, as a bluebird and a red arrow as a cardinal. The blue arrows are outward directed flux and the red, inward directed flux, most common in solar displays. The distribution of shades of color, e.g. pink to deep red, is both for artistry and to help one distinguish the individual fields and their motions. Aspects related to the sliders and the solar physics of this model are discussed in sections under FURTHER INFORMATION.

The Sun rotates with a period near 27 days as seen from the Earth, at the equator, and 33+ days in the polar regions Although the rotation of the Sun as it appears in the sky, with the North pole at the top, would be from left to right, because we show fields relative to the Sun's equator, the equatorial left to right rotation is removed. Differences from this motion is called surface diffential rotation. This Mercator map is made such that a longitude on the EQUATOR is fixed, by removing this "synodic rate," of one rotation per ~27 days. In such a reference frame, the Sun's polar regions "rotate backwards" (from right to left).

The model seen in this display evolves from newly arising remnants of active region fields shown by bursts of red and blue arrows at low latitudes within 30 degrees of the equator. The arrows show the direction of the field motions, relative to the fluid (the differential rotation and circulation do not affect the orientiation, but all the other forces do).

The current model partly follows algorithms developed by Craig Reynolds (the Boids model), and reconfigured into the agent-based Bird Flocking models (Netlogo Flocking and Starlogo Boids), and partly from knowledge of solar field motions, for example, the pioneering work of Sheeley, Nash, Wang, and Lean at NRL, who furthered Leighton's general solar field model. To accomplish these alternate approaches, algorithms invoke the following processes:

1) Large Scale Motions:  
 1.1) Coherency and Alignment:   
with like-fields tending to move together, but additionally spread from locations of high field strength to areas of low field strength;  
 1.2) Differential Rotation:   
wherein the field motions, in addition to the other aspects of their motion has a drift in accordance with the Newton and Nunn�s rotation rates on the surface of the Sun (the equator is chosen to show no rotation, which is done simply by setting the Differential Rotation A parameter, which controls the rigid body rotation of the Sun, to 0);  
 1.3) Meridional Circulation:   
wherein field lines move on the solar surface, carried by a surface flow from equator to poles. The exact value is controlled by a slider merid-flow.   
 1.4) Coriolis Acceleration:   
This is controlled by the angular and directed velocities, using the traditional formula; with a constant rotation rate ~27 days; however time in our model is variable, consequently a parameter, Cor(50), is used. As on the Earth, in the northern solar hemisphere, flow velocities accelerate such that, for example, in a high pressure region, an anticyclone forms rotating in a clockwise fashion, and vice-versa in the south. Thus, for the Earth, low pressure regions, like hurricanes rotate anti-clockwise in the northern hemisphere. For the Sun, the acceleration becomes significant; at v~5 m s-1, one finds for the duration of a solar rotation, the velocity vector rotates around a clockwise rotational radius of ~2 x 10^5 km; this is dependent upon latitude; when magnetic features are closer than this distance, typically the shorter range magnetic attractions dominate;  
 2) Flocking motions:  
These were originally controlled by parameters and slider bars, however, with growing numbers of solar parameters, the flocking parameters are now fixed in the program, but can be modified by changing the program:   
2.1) Coherency and Alignment: Patterned after bird flocking, but with like-fields tending to move together, but additionally spread outwards, by a feature that allows the fields to �look-ahead,� and thereby tend to move from regions of higher field strength to areas of lower field strength, thus spread out uniformly, as the field of single sign flux would do (e.g. numerous monopoles). VISION is the distance that each bird can see 360 degrees around it, to identify neighbors either of same or opposite color;  
2.2) Separation: wherein opposite fields move apart from each other, so as to evade death, however, if they were all to avoid death, numbers of birds would grow and then eventually be under increasingly closer confinement and deaths would inevitably rise until births and deaths became balanced on average. Three TURN-ANGLE parameters control the maximum angle the birds can turn, within a time step unit, as a result of each rule;  
 3) Birth:   
wherein new active regions are born in "low latitudes," proportional to the polar field strength, and in accord with "Hale's laws of sunspot polarities and active region tilts".   
 4) Death:   
if opposite color field lines get within a distance of "kill-dist" from each other, they both die;   
 5) Differential Rotation:   
wherein the field motions, in addition to other aspects has a rotation in accordance with the Newton and Nunn rotation rates of the surface of the Sun;   
 6) Meridional Circulation:  
wherein field lines move on the solar surface, carried by a surface flow from equator to poles. The fluid motions, 5 and 6, move the fluid in which the fields are embedded, thus the fields behave like birds flying in a wind tossed storm; and lastly   
 7) Polar Field Tension (the polar-B-force):  
wherein each field line is attracted by the tension of the subsurface Babcock-Leighton field lines, which generally wind around the Sun, from the North to the South Polar Regions. The field tension can be removed by using the slider-bar, polar-B-force, to zero, removing this most important guiding force, in this model. The toroidal field is not used in the current model, as differential rotation essentially provides a predominant longitudinal spreading of fields. As with other guiding forces (e.g. interaction of surrounding fields), unlike fluid drifts, the forces interact on each field by reorienting them minutely. Other processes also have "slider bars" described, from which various parameters, such as this force, can be reduced or augmented.

The field separation, coherency, and other parameters dealing with how tightly the field lines turn, etc. are removed from the program and set internally, so the display allows the reader to only change the solar parameters, etc.

  

## HOW TO USE IT

First, determine the number of polar fields you want to start with, in the simulation and set the START-POLES slider to those values. Press SETUP to create the birds, and press GO/STOP to have them rotating. Other settings will control different model aspects: e.g. the birth of active regions (POPULATIONBC controls the number of each colored birds born per new active region (AR)), etc. The default settings for the sliders will produce reasonably good dynamo behavior. Nevertheless, since the actual solar dynamo implements roughly a thousand active region for the given value of the polar field, we would have to have extremely few polar fields and that would prohibit viewing the fields for this dynamo simulation. This simulation shows many other interesting aspects of the dynamo that relate to surface features.

  

## THINGS TO NOTICE

First one can notice how the polar field keeps oscillating from blue to red; this is due to the 11 year solar cycle. Due the the difference in time units between our cycle and the solar cycle, the length is ~80 ticks. One model tick time unit corresponds to about 45-50 Earth days, or ~ two equatorial solar rotations. High latitude regions in the display rotate to the left, because of DIFFERENTIAL rotation (differences from a "rigid body rotation"). Thus Sun is a gaseous object, as are the outer planets, and their material often rotates around some central axis at differing rates. For the Sun, the equator rotates near 27 days as seen from Earth, but the highest latitudes take nearer 30-33 days The display ONLY shows the high latitudes moving to the left, which corresponds to a slower rotation rate. One can notice the general patterns of field motions (flows of similarly colored birds), which sometimes form "Unipolar Magnetic Regions" (backwards-C patterns, wherein fields drift towards the poles rotating to the left as they go). These were also called solar magnetic sectors in the space age, although they were supposed to look like the sliced sectors of an orange, their geometry was slightly different. The solar magnetic fields predominantly move towards weaker field regions (that is one of the bases of this model). This feature is similar to way heat flows from a hot object to a colder one, thus moving in the direction of equalizing temperatures. Other thermodynamic state variables (such as field intensity) do likewise. It is seen most prominently near the poles.

Central to the model is that the flows and fields move together. Thus one readily observes the differential rotation in which the high latitudes cause fields to drift towards the left. There are other flows one can observe: Coriolis forces, wherein fields that are FAR REMOVED from other field lines, flowing poleward in the northern hemisphere circulate clock wise, and vice-versa in the southern hemisphere. One can find plenty of counter examples to this, because most often, the magnetic forces overwhelm the Coriolis forces. Additionally meridional flow moves fields towards the poles. These just move the fluid in which the fields are embedded, like birds flying in a wind storm. the polar field exerts forces which acts like a flow towards the poles, similar to the meridional circulation flow. Instead, the meridional motion on a large scale is controlled only by polar field attraction to oppositely oriented polar fields, by an assumed subsurface field related to the NPOLE and SPOLE numbers. This provides a behavior similar to meridional circulation by the attraction of polar fields for lower latitude fields. This attracts or draws "following flux" to the poles in this model. Different behavior of field lines on small and large-scales in this model occurs, owing to the scale and location of these field lines.

As the model runs, one may notice because of the birth of fields between +- 30 degrees and the migration from equatorial latitudes towards the poles, that the low latitudes (<30 degrees) and the high latitudes (>60 degrees) have the largest density field lines. One may also notice unipolar regions, also called sectors, which are broad regions of generally "one sign" field, which persist on the Sun for awhile. The sectors on the Sun are continually changing (moving in longitude), and are the sorce of complex phenomena which affect "space weather," such as geomagnetic storms, and coronal mass ejections, affecting the electromagnetic "weather" that the Earth is bathed in, thus governs various solar-terrestrial relationships.

  

## THINGS TO TRY

Play with the sliders to see how the cycles behave. Just have fun with the various sliders, and hopefully you won't bomb the model. If you do, it is NO PROBLEM! If you manage to cause a problem with the model running, simply close your browser; then in a short while reopen the program start page, and you can try a new setting that is not so daring; the values should reset to the original ones. First, change any parameters you want to reset (via the slider bars). Press SETUP to create the initial polar fields, and press GO to have the fields (birds) gliding across the Sun's surface (or if you prefer the magnetic birds, cardinals and bluebirds for their colors, flying around on this distant world).

  

## FURTHER INFORMATION: INPUT SLIDERS SAMPLE VALUES BELOW. DISTANCE UNITS ARE IN GRID DISTANCES.

The default settings for the sliders will produce reasonably good behavior most of the time, one can play with them to get variations; nominal values are shown above.

Interface Parameter Nominal or Sample Value  
Tick-end 1000; 0 for continuous calculation.  
Random-seed 314159 or other #; 1 for random runs  
Populationbc 20  
Start-poles 20  
Merid-flow 0.1  
Cspeed 4  
Kill-dist 2.9  
Bspeed 4  
Velocity 4  
Quad-blaster 200  
Npole-degrees 50  
Polar-B-Force 50  
Joy�s-Law 7  
AR-rate 40  
B-DR-rate 1  
C-DR-rate 1  
DR-mult 0.2  
Butterfly? On  
All-Global-Random? Off  
Expo 8  
Expo2 0.5  
Expo3 1  
Cor 50  
Peak-lat 40  
Min-lat 5  
K2 3.1  
Dipole-or-quad? On  
Patch-color-white? Off  
Ssize 0.7  
DR-mult 0.2  
List-length 5  
Bird-reps 1  
Quant 5

  

## LESS IMPORTANT PARAMETERS:

ALL-GLOBAL-RANDOM [OFF] If OFF, the normal field birth prevails (Hale's law of field orientations). If ON then newly formed bipolar regions have random orientations and locations on the disk. This is just a test.... .

PATCH-COLOR-WHITE? [off] If on, a white background; useful to make plots of field.

  

## FIXED PARAMETERS, THAT THERE IS NOT NORMALLY ANY NEED TO MODIFY.

VISION [10] - Vision controls how far each bird/field sees in interacting with its extended neighborhood. In local interactions, aside from death, there are Alignment, Cohesion, Separation, and Avoidance.

AVOIDANCE-DISTANCE [20]- how far birds will examine each other, in order to consider separating further, from others of opposite colors to AVOID them. Of course, they do not succeed completely, as the density of birds builds up until the inevitable bird collisions occurs in this model, and opposite color birds both disappear.

MINIMUM-SEPARATION [1] - Provides the minimum separation in grid units, of SAME color field lines. Fields turn away to attempt to maintain more spacing than this minimum; it also affects coherence and avoidance because this is done with a higher priority. The priority of these factors may be something for further study.

MAX-ALIGN-TURN [5]- How rapidly the birds can turn towards each other to align, and flow together. The number is the number of degrees per time-step.

MAX-COHERE-TURN [3] - How rapidly the entities can align with cohesion, in degrees per time step.

MAX-SEPARATE-TURN [9.25] - Used in both field separation, as same sign birds spread out from each other, and in avoidance. This is how fast the birds can turn to avoid each other.

  

## FURTHER INFORMATION: MONITORS -

There are a number of monitors that display numbers as they are being updated, every so often, so one may check stats, and vary parameters for a variety of reasons:

COUNT-DEAD - Shows the number of + and - fields which have met their maker by meeting a member of the opposite color within a Moore neighborhood.

BCFIELD-COUNT - The number of both bluebirds and cardinals which have been placed in the photosphere. The blue corresponds to outward field and the cardinals to red, or inward field. We provide these bird names, without implying that the fields are fowl.

AR-COUNT - "active region" count. This is the number of bursts of field lines which form in the photosphere. In conventional "deep dynamos," this represents the number of eruptions of field lines from deep within the solar interior. Other processes may also allow field concentration to form in the photosphere. It is an unsolved problem, but regardless of the process or processes the Sun chooses to magnify its field into concentrated localities, active regions DO APPEAR. This model simply places ARs randomly at low latitudes in accord with Hale's law of polarities. This can be improved with a more complex model, but we are only trying to write the basic program of how field lines MOVE in the photosphere, and NOT their formation or origin.

BLUEBIRDS & CARDINALS - The number of alive birds/fields in the photosphere.

NPOLE & SPOLE - The "number" of field lines at either pole, and naturally their polarity. Sometimes, as with the Sun, both polarities are the same sign. This is a weighted count, in accord with Wang, Sheeley, and Nash's 1/theta to the eighth power falloff of polar fields. The field is not counted below Npole-degrees (about 50 degrees), in any case.

N-S-POLE - The difference of the above.

PLOTA - polar field (blue Npole, red Spole) AND TOTAL FIELD (absolute value of these two fields vs TIME in ticks timesteps); shifted downwards to displace from the above polar field values.- each unit is a timestep. The graph adjusts to fit whenever a value exceeds the current bounds.

PLOTB - Latitude of new active regions per unit time. This is like the "butterfly diagram" that solar physicists graph, showing how spots move equatorward versus time. in our model, when butterfly? is ON, then spots move equator as time progresses. Nevertheless, conventional graphs show ALL THE NEW active regions (AR), but this model only shows one dot per tick unit of time, and when there are no new spots, a value at zero latitude is graphed. Hence there are a lot of dots at zero latitude; the broad belt at +-10 degrees, shows spots at the lowest latitude band..

  

## FURTHER INFORMATION: SOLAR FIELDS

The fields displayed are not the tightly confined sunspots. The sunspots are most often discussed since they are the most visible magnetic feature. Rather the field agents displayed are their weaker remnants. These are the result of sunspots, when the sunspots break up. They leave magnetic elements called faculae and network fields. These are rarely seen, but ever-present. They are like the jetsam or flotsam from a ship, namely those remains of a ship lost at sea: respectively either jettisoned by the crew, or that which floats away on its own. So, too, the magnetic elements in the photosphere, may be viewed as the leftovers from the breakup of sunspots.

The faculae and network fields are of greater importance, they seem to have a longer lasting global impact, and help drive the Sun's future magnetic cycle, as pointed out by Babcock and Leighton in the 1960s. Leighton modeled the motions with diffusion to develop a dynamo model. More recently authors have used flow-driven field motions, which because of the Sun's high conductivity requires the field and flow to move in larger patterns together. The largest patterns have often been called "unipolar magnetic regions" (UMRs), or sectors, and their motions are often puzzling, and do not always move with the presumed meridional flow. The UMR motions can be seen in this model, as the colors spread from high field strength to low, and from equator to pole, with the UMRs representing areas of a single predominant polarity. This work is a cellular automata approach, which is different than the normal differential equation approach (using a "magnetic dynamo") undertaken by all other studies of solar magnetic field motions. In this work, we recognize that magnetic elements respond only to the local forces which act upon them.

Leighton, in the classical model, had a diffusive behavior of following flux to provide poleward motion similar to our attractive force. Preceding flux (that which precedes the active region as it rotates onto the solar disk) moves towards the "opposite pole" in the Leighton model, in accord with diffusive motion and "Joy's law" - which has the preceding part of an active region located at lower latitudes (a slider bar controls Joy's law). A more modern model is the work of Charbonneau, and Dikpati. These are of interest primarily to solar physicists, but not so apparent to non-afficianados of solar physics. The following random numbers determine the model's behavior: where, how many, and what size, etc. each new active region's fields are generated. Although the motion of the fields appear to be chaotic, the actual motion of the fields in this model is a result of determinism, governed by the position of all the other fields on the Sun's surface.

On the small scale, one may view the motions of individual fields relative to their neighbors, as individual entities, like birds in a flock, or a fish in a school, governed by local properties (their vision, avoidance distance, alignment, separation and cohesion, etc.). On the large scale, this model has larger scale "winds"; Sheeley, Nash, Wang, and Lean at NRL have shown general motions of field obey motions that appear very much like Bumba and Howard's "backwards C shape" unipolar magnetic regions (UMRs). Such regions are also called "sectors" by Wilcox. Nevertheless, the number of such sectors is often depicted to be 2 or 4, per rotation (the size of the Sun), nevertheless, when the number of new regions erupts in many places nearly at the same time, the Sun may have more of these patterns per rotation as our simulation shows. This goes against any simple rules that are generally thought true. It is hoped that we will see some such behavior on the Sun soon, but the current cycle seems particularly mild, so it may not happen during the next decade, but we are hopeful. The author may be contacted at kennuth1 AT comcast DOT net giving as much detail as possible, particularly any changed parameters and the random-see used.

Further information of the motion of the Sun's fields can be found here: http://en.wikipedia.org/wiki/Solar\_cycle   
with the blue and yellow time vs. latitude plot of magnetic fields seen near the end of the plot. These just represent the average field vs. latitude, not the individual locations (latitude and longitude) that these maps display. Nevertheless, one can see how the fields migrate from low to high latitudes by the yellow and blue bands. Active region fields contain a LOT of magnetic flux and to replenish the Sun's polar fields requires but one in a thousand. This makes the solar cycle a very inefficient process with 99.9% of the field being "wasted" as the active region magnetic flux simply cancels before it makes its way to the opposite poles to start another solar cycle.   
The strength of active region fields compared with the background field may be seen in recent field plots at the following sources:   
http://www.swpc.noaa.gov/ws/gong\_phfld\_last3.html  
http://gong.nso.edu/info/press/GONG\_magfield\_pr/  
http://jsoc.stanford.edu/data/hmi/images/latest/HMI\_latest\_Mag\_256x256.gif  
Included are individual images of the Sun's fields as well as synoptic maps of the Sun's full surface. Active region fields show magnetism covering the disk as well as the enormous field variations present on the Sun's surface, with weak polar fields barely visibles in the earlier displays, when the Sun's field was very active in the last half of the 20th century. Recent fields, such as the HMI images from NASA's Solar Dynamics Observatory, show weak magnetism, but again the active region fields are still extremely strong compared with background fields. Information on the toroidal fields inside the Sun  
is found here: http://ulrich.astro.ucla.edu/papers/toroidal.pdf

A site that is student friendly, for studying the Sun, is located at:  
http://solar-center.stanford.edu/credits.html  
More information about NASA's recent solar satellite SDO may be found here:  
http://sdo.gsfc.nasa.gov/

  

## SOLAR FIELD AND DYNAMO REFERENCES

http://www.scholarpedia.org/article/Solar\_dynamo  
http://ulrich.astro.ucla.edu/papers/toroidal.pdf  
Babcock, H.W., 1961, Ap. J., 133, 572.  
Bumba, V., and Howard, R. F., 1965, Ap. J., 141, 1492.  
Charbonneau, P., Dikpati, M., 2000, �Stochastic Fluctuations in a Babcock-Leighton Model of the Solar Cycle�, Astrophys. J., 543, 1027�1043.  
Hale, G. E., 1908, Ap. J., 28,100,315. DOI: 10.1086/141602  
Leighton, Robert B.,Ap J, vol. 156, p.1 04/1969 DOI: 10.1086/149943  
Leighton, R.B., 1964, �Transport of magnetic fields on the sun�, ApJ, 140, 1547�1562.  
Schatten, K.H., Leighton, R.B., Howard, R., Wilcox, J.M., 1972, Solar Phys., 26, 283.   
Schatten, K., Solar Phys., 2009, 255, 3.  
Sheeley Jr, N.R., Nash, A.G., Wang, Y.-M., 1987, �The origin of rigidly rotating magnetic field patterns on the sun�, Ap. J., 319, 481�502.   
Y.-M.Wang, N. R. Sheeley, Jr., and J. Lean Ap. J.,580:1188�1196, 2002.

  

## NETLOGO FEATURES, CREDITS, REFERENCES, AND CITATION

This model may be referenced by Schatten, K. H. (availability, by current site).  
This began as a take-off of the Boids simulation invented by Craig Reynolds. Information on Boids is available at http://www.red3d.com/cwr/boids/.   
This model was developed using Netlogo: - Wilensky, U. (1998). NetLogo Flocking model. http://ccl.northwestern.edu/netlogo/models/Flocking. Center for Connected Learning and Computer-Based Modeling, Northwestern University, Evanston, IL. - Wilensky, U. (1999). NetLogo. http://ccl.northwestern.edu/netlogo/. Center for Connected Learning and Computer-Based Modeling, Northwestern University, Evanston, IL. In other publications, please use: - Copyright 1998 Uri Wilensky. All rights reserved. See http://ccl.northwestern.edu/netlogo/models/Flocking for terms of use.

  

## PROCEDURES

```
;Solar Field Mapping Program 1.07 (or 1p07)
; the user may contact Kenneth Schatten for how to use program

globals [mean-blue-y mean-card-y AR-location-x AR-location-y count-dead npole-lat spole-lat 
   npole spole tilt quad sign-quad temp temp2 AR-count bcfield-count n-s-pole n-s-pole-old 
   abs-pole tempxx tempy old-tick mod-tick vision avoidance-distance minimum-separation max-align-turn max-cohere-turn
   max-separate-turn off-track    short-list s-list center-peak? new-value central-position   
     average-Bp peak-Bp peak-time K1 solar-cycle-length average-cycle-length delta-lat lat-AR lat-AR-last peak-time-list lat-min-time
     old-npole old-spole Ncycles  ]
 

breed [bluebirds bluebird   ] 
breed [cardinals cardinal]    ; cardinals are red 

bluebirds-own [ blue-flockmates nearest-blue-neighbor bbspeed card-flockmates nearest-card-neighbor bgeometry other-nearby]
;  I think it was all fine... i just had extra thing defined  blue-neighbors in card own, but not used..
;; the cardinals own seems like it was incorrect!!!!!!!;;; i have blue-neighbors in cards own, but not vice versa... so removing
;blue-neighbors in cards-own..
cardinals-own [ blue-flockmates nearest-blue-neighbor ccspeed card-flockmates nearest-card-neighbor cgeometry other-nearby]

turtles-own [ tempx phi theta distance-from-card nearest killed? partner]


to setup
  clear-all
 
  setup-patches
  if random-seed-1=YES != 1
  [ random-seed random-seed-1=YES ]
    set off-track 0
    set Ncycles 0
    set vision 10
    set avoidance-distance 20
    set minimum-separation 1
    set max-align-turn 5
    set max-cohere-turn 3.25
    set max-separate-turn 9.25
   set mod-tick 1
   set abs-pole .1
   set AR-count 0
   set n-s-pole 0
   set n-s-pole-old 0
   set short-list n-values list-length [0]
  set central-position int (list-length / 2 )
   set peak-Bp 40
   set peak-time 0
   set lat-min-time 0
    set delta-lat 10
    set average-cycle-length 20
    set average-Bp 40
    set K1  (peak-lat - min-lat) / average-cycle-length
    set peak-time-list []
  set bcfield-count 0 ;  count blues and reds 
  set count-dead 0
   set npole-lat npole-degrees * max-pycor / 90  ; ~60 deg
  set spole-lat 0 - npole-lat
 ; set gcf 00.0
  ifelse All-Global-Random? [
    create-random-dipole
    ]
 
[ ifelse dipole-or-quad? 
  [ create-cardinals start-poles ;populationc
    [     set killed? false
      set partner nobody
       set bcfield-count bcfield-count + 1
      set color red - 2 + random 7  ;; random shades look nice
     set size .5 * ssize * ( 1.01 / ( .01 + abs ( sin ( .9)))) ^ .5 ;; easier to see
      setxy random (2 * max-pxcor)  max-pycor * -0.99 ] ;random-xcor random-ycor ]
    create-bluebirds start-poles ;populationb
     [     set killed? false
       set partner nobody
       set bcfield-count bcfield-count + 1
        set color blue - 2 + random 7  ;; random shades look nice
        set size .5 * ssize * ( 1.01 / ( .01 + abs ( sin ( .9)))) ^ .5 ;; easier to see
    
      setxy   random (2 * max-pxcor) max-pycor * 0.99]  ;   random-xcor random-ycor ]
  ]  
 ; poles: 
  [     create-cardinals start-poles / 2 ;populationc
    [      set killed? false
      set partner nobody
      set color red - 2 + random 7  ;; random shades look nice
     set size .5 * ssize * ( 1.01 / ( .01 + abs ( sin ( .9)))) ^ .5  ;; easier to see
      setxy random (2 * max-pxcor)  max-pycor * -0.99 ]
    create-cardinals start-poles / 2 ;populationc
    [      set killed? false
      set partner nobody
      set color red - 2 + random 7  ;; random shades look nice
     set size .5 * ssize * ( 1.01 / ( .01 + abs ( sin ( .9)))) ^ .5 ;; easier to see
      setxy random (2 * max-pxcor)  max-pycor * 0.99 ] ;random-xcor random-ycor ]
 
    create-bluebirds start-poles ;populationb here fields are being added to equator..
     [      set killed? false
       set partner nobody
       set bcfield-count bcfield-count + 1
       set color blue - 2 + random 7  ;; random shades look nice
      set size .5 * ssize * ( 1.01 / ( .01 + 0)) ^ .5 ;; easier to see
      setxy   random (2 * max-pxcor) 0  ]
  ]]    
    do-plots
end


to ask-Bp ; does nothing now..
  end


to  create-random-dipole

     set tempxx random (2 * max-pxcor)
     set tempy  max-pycor * ( asin (( random 200 - 100) / 100  ) / 90 )
     create-cardinals 1 [ 
           set killed? false
           set partner nobody
           set ccspeed (random (2 * Quant * cspeed)) / Quant
      set bcfield-count bcfield-count + 1
      set color red - 2 + random 7  ;; random shades look nice
      set size .5 * ssize * (1.01 / ( .01 + abs ( sin (tempy / max-pycor)))) ^ .5  
      setxy tempxx tempy ] ;random-xcor random-ycor ]
    create-bluebirds 1 [
           set killed? false
           set partner nobody
            
       set bbspeed (random (2 * Quant * bspeed)) / Quant
       set bcfield-count bcfield-count + 1
      set color blue - 2 + random 7  ;; random shades look nice
      set temp random 360
       set temp2 (tempy + cos temp)
       if (temp2 > max-pycor) [set temp2 max-pycor]
        if (temp2 < (0 - max-pycor)) [set temp2 (0 - max-pycor)]
      setxy (tempxx + sin temp) ( temp2)
      set size .5 * ssize * ( 1.01 / ( .01 + abs ( sin (temp2 / max-pycor)))) ^ .5
      ]
end

to setup-patches
  if patch-color-white? [ask patches [ set pcolor white ]]
end

to kill
  if (abs-pole > 2 and killed?) [set count-dead count-dead + 1 die]
end

to  fluid-flow
  diff-rot 
  meridional-flow
end


to bird-flock
end
;(PUT ALL BIRD Flocking behavior here, and shorten elsewhere?)


to go
  if (TICK-END != 0) [if (ticks >= TICK-END) [stop]]
  fluid-flow
  ask cardinals [ card-flock ]
  ask bluebirds [blue-flock]
 ask bluebirds [blue-coriolis-accel]
ask cardinals [red-coriolis-accel]
  fluid-flow
   ask cardinals [blue-avoid ]
  ask bluebirds [card-avoid]
  fluid-flow
   ask cardinals [cgeom ]
  ask bluebirds [bgeom]
;  fluid-flow
  repeat bird-reps 
  [ask bluebirds [blue-Great-Circle-Force] ask bluebirds [fd velocity * bbspeed * bgeometry *  (abs-pole + 15) ^ expo3 / 10000  ] ; was (abs-pole * 50)]
     ask cardinals [red-Great-Circle-Force] ask cardinals [fd velocity * ccspeed * cgeometry *  (abs-pole + 15) ^ expo3 / 10000  ] display ]  ; was (abs-pole * 50)] display         ]
 fluid-flow
  ask cardinals [  set size .5 * ssize * ( 1.01 / ( .01 + abs ( sin (ycor / max-pycor)))) ^ .5 ]
  ask bluebirds [  set size .5 * ssize * ( 1.01 / ( .01 + abs ( sin (ycor / max-pycor)))) ^ .5 ]
; fluid-flow
 set  mean-card-y 0  ;(mean[ ycor] of cardinals)
  set  mean-blue-y 0;(mean[ ycor] of bluebirds)
  fluid-flow
          if (ticks mod mod-tick = 1) [pole-count] 
 ask turtles [polar-drift]
 repeat 1 [fluid-flow]
ask cardinals
  [if  (nearest-blue-neighbor != 0 )
      [ if ( nearest-blue-neighbor != nobody )      ; was 2-
      [ if (abs-pole > 2 ) [ if ( (cgeometry ^ 0.1) * distance nearest-blue-neighbor < kill-dist) and (partner = nobody)  
     [ set partner  nearest-blue-neighbor
     
      ask partner [
         set partner myself
       
          set count-dead count-dead + 1   die   ]
    set count-dead count-dead + 1   die
    ]
   ]
   ]
   ]
  ]
repeat 1 [fluid-flow]
  
  if  ( off-track =  0 ) [add-new-ARs]
  repeat bird-reps [ ask cardinals [red-Great-Circle-Force] ask cardinals [fd velocity * ccspeed * cgeometry * (abs-pole + 15) ^ expo3 / 10000 ] 
     ask bluebirds [blue-Great-Circle-Force] ask bluebirds [fd velocity * bbspeed * bgeometry *  (abs-pole + 15) ^ expo3 / 10000 ] display         ]  
   ask bluebirds [ blue-flock ]
  ask cardinals [card-flock]
   repeat bird-reps [ask cardinals [red-Great-Circle-Force] ask cardinals [fd velocity * ccspeed * cgeometry * (abs-pole + 15) ^ expo3 / 10000 ] 
    ask bluebirds [blue-Great-Circle-Force]  ask bluebirds [fd velocity * bbspeed * bgeometry *  (abs-pole + 15) ^ expo3 / 10000 ] display         ]
 ;;  repeat 10 [ wait 0.5 ]
repeat 1 [ fluid-flow] 
   ask bluebirds [card-avoid ]
  ask cardinals [blue-avoid]
   repeat bird-reps [ask cardinals [red-Great-Circle-Force] ask cardinals [fd velocity * ccspeed * cgeometry * (abs-pole + 15) ^ expo3 / 10000 ] 
    ask bluebirds [blue-Great-Circle-Force] ask bluebirds [fd velocity * bbspeed * bgeometry *  (abs-pole + 15) ^ expo3 / 10000 ] display         ] 
  fluid-flow
   ask bluebirds [bgeom ]
  ask cardinals [cgeom]
   repeat bird-reps [ ask cardinals [red-Great-Circle-Force] ask cardinals [fd velocity * ccspeed * cgeometry * (abs-pole + 15) ^ expo3 / 10000 ] 
    ask bluebirds [blue-Great-Circle-Force]  ask bluebirds [fd velocity * bbspeed * bgeometry *  (abs-pole + 15) ^ expo3 / 10000 ] display         ]
 ;2 fluid-flow
  repeat bird-reps [ask cardinals [red-Great-Circle-Force]  ask cardinals [fd velocity * ccspeed * cgeometry * (abs-pole + 15) ^ expo3 / 10000 ] 
    ask bluebirds [blue-Great-Circle-Force]  ask bluebirds [fd velocity * bbspeed * bgeometry *  (abs-pole + 15) ^ expo3 / 10000 ] display         ]
 fluid-flow
 pole-count
  set abs-pole (abs npole + abs spole  + .1)
 ifelse (abs-pole >  100 + abs spole + abs npole ) 
 [set off-track 1]  ;;; problem shooting off to infinity..
 [set off-track 0]

 ask turtles [polar-drift]
  fluid-flow
  add-new-ARs
  
  tick    
  
   set new-value abs-pole
  
  set short-list lput new-value but-first short-list
  set s-list  map [round ?] short-list
  
  set-central-peak?
  ifelse (abs npole = 0 or abs spole = 0) []
 [ if (abs npole > 0 or abs spole > 0) [
  ifelse (center-peak? and (npole / abs npole = 0 - spole / abs spole) and (abs (npole - spole) > 8 and 
      (ticks - peak-time) > 10 ) and (ticks > 30 or sign npole != old-npole and sign spole != old-spole)
  )
  
    [ set old-npole sign npole
      set old-spole sign spole
      set Ncycles Ncycles + 1
      set peak-Bp max short-list
    set peak-time (ticks - (list-length / 2))
   set peak-time-list lput (1 + int peak-time) peak-time-list
   set lat-min-time -60 + 10 ;;; was60  ;CHANGED
    ]
    [set lat-min-time -60]
  ]]
  
  fluid-flow
  do-plots
  quad-blaster1  
  
end


to blue-Great-Circle-Force
  
  ; change the heading amount to same as velocity:
  ;velocity * bbspeed * bgeometry *  (abs-pole + 15) ^ expo3 / 10000
    ifelse (heading < 180 ) [set heading heading   + 
    velocity * bbspeed * bgeometry *  (abs-pole + 15) ^ expo3 / 10 * gcf ^ 0.8  * (tan (ycor / max-pycor)) / (cos (ycor / max-pycor)) ]
     
 [set heading heading   - 
   velocity * bbspeed * bgeometry *  (abs-pole + 15) ^ expo3 / 10 * gcf ^ 0.8  * (tan (ycor / max-pycor)) / (cos (ycor / max-pycor)) ]
 
end
to red-Great-Circle-Force
   ifelse (heading < 180 ) [set heading heading   +  
     velocity * ccspeed * cgeometry *  (abs-pole + 15) ^ expo3 / 10 * gcf ^ 0.8  * (tan (ycor / max-pycor)) / (cos (ycor / max-pycor)) ]
     
 [set heading heading   - 
   velocity * ccspeed * cgeometry *  (abs-pole + 15) ^ expo3 / 10 * gcf ^ 0.8  * (tan (ycor / max-pycor)) / (cos (ycor / max-pycor)) ]
 
end
to set-central-peak?
   ifelse  (max short-list = item central-position short-list) 
  [set center-peak? true  ]
  [set center-peak? false ]

end


to bgeom  
  set bgeometry (1 + abs (sin (90 * ycor / max-pycor ) * cos (90 * ycor / max-pycor ) / .5)  ) * ((max-pycor - abs ycor)  / max-pycor) ^ ( 2 * EXPO2)
end
to cgeom  
  set cgeometry (1 + abs (sin (90 * ycor / max-pycor ) * cos (90 * ycor / max-pycor ) / .5)  ) * ((max-pycor - abs ycor) / max-pycor) ^ ( 2 * EXPO2)
end

to add-new-ARs 
   ifelse All-Global-Random? [
    create-random-dipole
    ]
  [  set solar-cycle-length  (average-cycle-length * average-Bp / (1 + peak-Bp))
; fine
     set temp2 AR-rate * K2 * abs-pole * (1 - (cos (90 * (ticks - peak-time) / solar-cycle-length)) ^ 2) / (2 * 2000)
     ;( 1. - 0.5) / ( solar-cycle-length * 2 * 2000)
     ;(1 - (cos (90 * (ticks - peak-time)) ^ 2 / solar-cycle-length)) / (2 * 2000)
     ifelse (temp2  > 1 )
    [ repeat  temp2 [ add-an-AR]]  
    [ifelse (random 200 < temp2 * 200 ) [  add-an-AR] [dont-add-AR]]]
     
    end

to dont-add-AR
  set lat-AR 0
end

to add-an-AR 
         set AR-count AR-count + 1
      set AR-location-x random (2 * max-pxcor)
       ifelse butterfly? 
       [set temp2 peak-lat - (ticks - peak-time) * K1  
         if (temp2 < 0) [set temp2 0]
           set temp (2 * (random 2) - 1 ) * (temp2 + random delta-lat)  ]
             [set temp (random 60 - 30)] ;; this is 30 to -30
           set lat-AR-last lat-AR
         set lat-AR temp 
        set AR-location-y ( temp) * max-pycor / 90
     
        set tilt  temp * Joy's-law / 20  
     create-cardinals populationbc [ 
            set killed? false
            set partner nobody
      set ccspeed (random (2 * Quant * cspeed)) / Quant
      set bcfield-count bcfield-count + 1
      set color red - 2 + random 7 
      set temp sign AR-location-y * random 3 
      setxy AR-location-x - temp * sign(n-s-pole)    AR-location-y
      set size .5 * ssize * (1.01 / ( .01 + abs ( sin (AR-location-y / max-pycor)))) ^ .5 ] 
      create-bluebirds populationbc [
           set killed? false
           set partner nobody
       set bbspeed (random (2 * Quant * bspeed)) / Quant
       set bcfield-count bcfield-count + 1
      set color blue - 2 + random 7  ;; random shades look nice
       set size .5 * ssize * (1.01 / ( .01 + abs ( sin ((AR-location-y + 2 * temp * tilt / 57.3) / max-pycor)))) ^ .5 
      setxy AR-location-x + temp * sign(n-s-pole)     (AR-location-y - 2 * temp * tilt / 57.3)
       ]  
end


 ;; w = a + b sin^2 theta + c sin^4 theta
 ; a = 14.7 b= - 2.4 c = -1.8 degrees per day
to diff-rot
 ask turtles
 [     ; the 14.7 is removed, because we insist upon no DR at equator..
       set temp2 (sin (90 * ycor / max-pycor )) ^ 2
       set tempx  xcor   -  DR-mult * ( B-DR-rate *  2.4 * temp2 + C-DR-rate * 1.8 * temp2 ^ 2) 
  set  xcor tempx
   ; render-turtle
  ]
end

to meridional-flow
  ;; yields an error below at asin...
; merid-flow is towards poles... proportional to sin lat cos lat
; lat is asin ycor
 ask turtles 
  [ 
       if (abs ycor < max-pycor) [ 
       set temp  asin (ycor / max-pycor) ; -90 to + 90
       set temp2 temp + merid-flow * sin temp * cos temp
      set ycor max-pycor * sin temp2
       ]]
end

to quad-blaster1
  ifelse (sign npole = sign spole) [set quad 1] [set quad 0 set sign-quad 0]
  if (quad = 1) [ set sign-quad sign npole
    ask turtles [ equator-drift  ]  ]
end

to equator-drift
  ;; fractionally turn heading!!
    if (quad-blaster != 0) ;   WAS if polarB-wind? 
 [ ifelse (breed = bluebirds)[ set heading heading   +  sign ycor * sign-quad * sin heading  * polar-B-force * quad-blaster / 3000]  
  [ set heading heading   -  sign ycor * sign-quad * sin heading * polar-B-force * quad-blaster / 3000]
 ]  
end


to polar-drift
  ;; fractionally turn heading!!
if ( polar-B-force != 0) 

 [ ifelse (breed = bluebirds)[ set heading heading   +  (npole - spole) * sin heading  * polar-B-force / 1000] 
  [ set heading heading   -   (npole - spole) * sin heading * polar-B-force / 1000]
 ]  
end

to pole-count
  set npole 0
  set spole 0
  ; for geometry, with an exponential number of 8, the mean factor above 50 degrees is 1.55 a normalizing factor
   ; that counts the field for 1 pole as 1 field line..
  ask bluebirds [if ycor > npole-lat [set npole npole + 1.55 * (ycor / max-pycor) ^ expo]]  
  ask bluebirds [if ycor < spole-lat [set spole spole +  1.55 * (abs ycor / max-pycor) ^ expo]]
   ask cardinals [if ycor > npole-lat [set npole npole -  1.55 * (ycor / max-pycor) ^ expo]]  
  ask cardinals [if ycor < spole-lat [set spole spole -  1.55 * (abs ycor / max-pycor) ^ expo]]
  set n-s-pole-old n-s-pole
  set n-s-pole npole - spole
end

to find-distance
  set distance-from-card distancexy 0 0 ; or xcor ycor  ; or 0 0
 ; (distance (xcor-of 0) (ycor-of 0))
end
  
  to-report sign [number]
  ifelse number >= 0
    [ report 1 ]
    [ report (- 1) ]
end


to do-plots
  
    set-current-plot "plot A"
  set-current-plot-pen "npole"
  plot npole
  set-current-plot-pen "spole"
  plot spole
   set-current-plot-pen "lat-min-time"
  plot lat-min-time
  set-current-plot-pen "abs-pole - 60"
  plot (abs-pole - 60)
   
    set-current-plot "plot B"
  set-current-plot-pen "lat-AR"
 ;   set-current-plot-pen "lat-min-time"
 ; plot lat-min-time
 ; ifelse (lat-AR = lat-AR-last)
  ;[set lat-AR 99]
  ;[plot lat-AR]
  plot lat-AR 

end


to blue-coriolis-accel;; turtle procedure
    ;; fractionally turn heading!!
 set heading heading   - 2 * Cor * (ycor / max-pycor) * bspeed  / 1000  ; ; speed based on indiv motion.
   end

to red-coriolis-accel;; turtle procedure
    ;; fractionally turn heading!!
 set heading heading   - 2 * Cor * (ycor / max-pycor) * cspeed  / 1000  ; speed based on indiv motion.
   end


to blue-flock  ;; turtle procedure
  find-blue-flockmates
  if any? blue-flockmates
    [ find-nearest-blue-neighbor
      ifelse distance nearest-blue-neighbor < minimum-separation
        [ separate-blue ]
        [ align-blue
          cohere-blue ] ]
end

to card-flock  ;; turtle procedure
  find-card-flockmates
  if any? card-flockmates
    [ find-nearest-card-neighbor
      ifelse distance nearest-card-neighbor < minimum-separation
        [ separate-card ]
        [ align-card
          cohere-card ] ]
end

to blue-avoid  ;; turtle procedure
  find-blue-flockmates
  if any? blue-flockmates
    [ find-nearest-blue-neighbor
      ifelse (distance nearest-blue-neighbor)  < AVOIDANCE-DISTANCE * cgeometry 
        [ separate-blue ]
        [ ] ]
end
    
to card-avoid  ;; turtle procedure
  find-card-flockmates
  if any? card-flockmates
    [ find-nearest-card-neighbor
      ifelse (distance nearest-card-neighbor)  < AVOIDANCE-DISTANCE * bgeometry
        [ separate-card ]
        [ ] ]
end


to find-blue-flockmates  ;; turtle procedure
  set blue-flockmates other bluebirds in-radius vision
end

to find-card-flockmates  ;; turtle procedure
  set card-flockmates other cardinals in-radius vision
end

to find-nearest-blue-neighbor ;; turtle procedure
  set nearest-blue-neighbor min-one-of blue-flockmates [distance myself]
;    set nearest nearest-blue-neighbor [distance-from-card] 

end

to find-nearest-card-neighbor ;; turtle procedure
  set nearest-card-neighbor min-one-of card-flockmates [distance myself]
end


;;; SEPARATE

to separate-blue  ;; turtle procedure
  turn-away ([heading] of nearest-blue-neighbor) max-separate-turn
end

;;; ALIGN

to align-blue  ;; turtle procedure
  turn-towards average-blue-flockmate-heading max-align-turn
end

to-report average-blue-flockmate-heading  ;; turtle procedure
  ;; We can't just average the heading variables here.
  ;; For example, the average of 1 and 359 should be 0,
  ;; not 180.  So we have to use trigonometry.
  let x-component sum [sin heading] of blue-flockmates
  let y-component sum [cos heading] of blue-flockmates
  ifelse x-component = 0 and y-component = 0
    [ report heading ]
    [ report atan x-component y-component ]
end

;;; COHERE

to cohere-blue  ;; turtle procedure
  turn-towards average-heading-towards-blue-flockmates max-cohere-turn
end

to-report average-heading-towards-blue-flockmates  
  let x-component mean [sin (towards myself + 180)] of blue-flockmates
  let y-component mean [cos (towards myself + 180)] of blue-flockmates
  ifelse x-component = 0 and y-component = 0
    [ report heading ]
    [ report atan x-component y-component ]
end

;;; SEPARATE

to separate-card  ;; turtle procedure
  turn-away ([heading] of nearest-card-neighbor) max-separate-turn
end

;;; ALIGN

to align-card  ;; turtle procedure
  turn-towards average-card-flockmate-heading max-align-turn
end

to-report average-card-flockmate-heading  ;; turtle procedure
  let x-component sum [sin heading] of card-flockmates
  let y-component sum [cos heading] of card-flockmates
  ifelse x-component = 0 and y-component = 0
    [ report heading ]
    [ report atan x-component y-component ]
end

;;; COHERE

to cohere-card  ;; turtle procedure
  turn-towards average-heading-towards-card-flockmates max-cohere-turn
end

to-report average-heading-towards-card-flockmates  ;; turtle procedure
  ;; "towards myself" gives us the heading from the other turtle
  ;; to me, but we want the heading from me to the other turtle,
  ;; so we add 180
  let x-component mean [sin (towards myself + 180)] of card-flockmates
  let y-component mean [cos (towards myself + 180)] of card-flockmates
  ifelse x-component = 0 and y-component = 0
    [ report heading ]
    [ report atan x-component y-component ]
end

;;; HELPER PROCEDURES

to turn-towards [new-heading max-turn]  ;; turtle procedure
  turn-at-most (subtract-headings new-heading heading) max-turn
end

to turn-away [new-heading max-turn]  ;; turtle procedure
  turn-at-most (subtract-headings heading new-heading) max-turn
end

;; turn right by "turn" degrees (or left if "turn" is negative),
;; but never turn more than "max-turn" degrees
to turn-at-most [turn max-turn]  ;; turtle procedure
  ifelse abs turn > max-turn
    [ ifelse turn > 0
        [ rt max-turn ]
        [ lt max-turn ] ]
    [ rt turn ]
end
```
