# Supplementary material for: Large-scale solar magnetic field mapping: I
Source: Springerplus. 2013 Jan 23;2:21. doi: 10.1186/2193-1801-2-21 (PMC3601253; doi:10.1186/2193-1801-2-21)
Supplement: Supplementary file 1 — Additional file 1: Movie 1: Shown is the Display section of the Solar Field Mapping model’s Interface vs. time for about 2 solar cycles. The movie can be viewed with Apple’s Quicktime movie viewer. It was made with a model identical to the model described in this paper, but written so as to allow movies to be made into a mov file. The movie can be stopped, or played at a slower speed with the Quicktime movie viewer. Movie 2. Similar to movie 1, however, the time period of the movie is extended; this allows one to see many solar cycles in the Display mode. (ZIP 2 MB) [file 40064_2012_134_MOESM1_ESM.zip › Solar Field Mapping Model Information and code.doc]

SOLAR FIELD MAPPING MODEL DEVELOPMENT INFORMATION AND USER CODE:

MODEL DEVELOPMENT INFORMATION

We discuss the model development and later herein, further information and the Netlogo code, although one needs to use either the Netlogo applet (Netlogolite.jar and the attached code: Solar Field Mapping 1p07.html), and/or add the Netlogo code (Solar Field Mapping 1p07.nlogo) to a Netlogo installation in order to have the program discussed in this paper run. To understand the physical motivation for this model, consider the following thought experiment. Firstly, one may restrict the volume of any model or theory to any volume one wishes, as the laws of physics are obeyed both globally and locally. Thus they are obeyed within any arbitrary volume that one chooses. In this model, we restrict consideration to a thin volume near the Sun’s photosphere. This model does thus ignores the more than 99+% of the Sun’s mass. Additionally, it focuses on where the observations of the magnetic fields and flows are best observed. No restriction comes without penalty. The model’s penalty is that it can say nothing about what happens outside the domain of its consideration or pervue.

Next one considers the Sun be emptied of magnetic field and that a bipolar magnetic region be placed on the Sun’s surface. Let the magnetic flux be transported freely around on this surface and, for simplicity, consider that above the surface, the field is carried out radially into the solar wind; what would be the minimal energy configuration, without allowing field cancellation? Since we are not allowing cancellation, the answer is that the fields move on the surface into a “monopole” pattern containing two equal and opposite hemispherical surface fields: one containing uniform inward directed field, and the other uniform outward directed magnetic field. The reason is that this contains the same number of field lines (double the magnetic flux,$\Phi=∯ \left| B\cdot\text{dA} \right|$ through the dipole: each outward and inward field is counted once) when integrated over the photosphere, but the integral of $B^{2}/8\pi$ over the photosphere is lowest through having its absolute value being a uniformly radially outward value, as a magnetic monopole would have, namely no concentration in any area. Non-uniform values create an imbalance, thereby requiring extra energy. This is the magnetic equivalent, in terms of thermodynamic intensive variables, of having a uniform temperature value in thermal equilibrium, or a uniform pressure value in pressure balance equilibrium, and constant B, in magnetic equilibrium. This lowest energy state has been used before to explain magnetic equilibrium in the current sheet model (Schatten, 1971). Thus, one can think of the present model, as allowing the fields to move towards their lowest energy states, by having newly situated fields to freely move on the Sun’s surface towards those areas of weakest field magnitude. This is the essence of the field mapping model.

Now we discuss our use of a 2D domain for the field motions, and then how the model treats solar magnetism, followed by a broad outline of cellular automata, and why we are using it, rather than the standard approach of differential equations. Put succinctly, an overview of the model is the following: The modeling is carried out by agents which must follow the Netlogo subroutines. The agents only have access to the quantities that are “given” to them. These are such things as their location, velocity, etc., as well as their nearest neighbors. All the Netlogo field agents are “bird” entities that contain quantized magnetic fluxes on the solar surface. We have ascribed to the bird agents, two “breeds,” one when the magnetic field is directed radially inwards, we refer to as cardinals (because inward solar field and cardinals are described most often as red), and when the field is pointed outward, we use the bluebird breed. Keeping track of things this way, allows us to easily compare “like-sign” fields and unlike-sign fields. In the next subsections we describe the agent processes more fully; these are their births, motions and deaths. In a nutshell, the model describes field agent processes: fields are born, move about on the solar surface by a number of flows and forces we describe, and eventually they die, when they come too close to an opposite breed member.

Put succinctly, our model can be explained thusly: agents are born in active regions through our random number generator, move about on the solar surface by a number of flows and forces we describe, and die. There are but a few effects agents have on other agents: 1) while at the poles, field agents are connected via the Babcock-Leighton (B-L) subsurface field to lower latitudes which allows them to undertake two duties there: A) the B-L subsurface field spawns the next generation of new magnetic flux, and B) the B-L subsurface field attracts lower-latitude fields via its long-range magnetic tension; 2) nearby agents affect each other’s motion by short-range interactions; and 3) through deaths: when opposite flux agents get too close to each other, they die off in pairs. We now describe the thinking behind these processes more fully.

What is the basis that allows us to turn a 3D problem of fluid motions carrying magnetic fields in the Sun’s photosphere into a 2D problem? We address this here. The model first neglects the small flow thru the photosphere into the chromosphere and corona, owing to the density stratification (a factor of 10^-12^); thus we consider the flow from below to simply overturn at the photospheric boundary. This makes the photosphere a unique location in the flow field, aside from its role in radiative transport. It is a surface with near zero normal (perpendicular to its surface) velocity. When the flow field is thusly constrained, the region is referred to as a boundary layer. This kind of thing happens, between the Earth’s lower atmosphere when it interacts with the Earth’s surface, called the atmospheric boundary layer and has a typical thickness less than a km. On the Earth, the lower atmospheric boundary layer varies greatly throughout the day, because of the changing solar insolation. There are many situations involving boundary layers; we shall mention a few such cases, so that the reader can recognize the commonality: the surface of molten volcanic magma inside calderas; the flow of a boiling pot of water at the surface liquid-air interface, as well as at the bottom and sides of the pot; and the flow fields over rigid surfaces, such as wings or other objects. Although the volume of the fluid is three-dimensional, we consider the motion only of the fluid in the boundary layer, thereby turning the 3D problem into a problem involving the 2D motion of the field parallel to the boundary layer, since the velocity perpendicular to the photospheric thin layer is near zero.

Basically, we are assuming that fields are brought to the photosphere, and then can skate on the Sun’s surface towards regions of lowered potential energy. This is essentially the assumption we make that allows us to predominantly ignore the vertical aspects of this difficult problem. It is not that we do not believe the problem is a 3D problem; it is simply that the photosphere represents a unique boundary layer that inhibits motion of significant amounts of material higher than its surface. This is similar to the cork model assumption that Van Ballegooijen et al (1998) developed wherein fields in the model move around the surface driven solely by flows, which advect the field. We also DO use the third dimension through our use of the B-L magnetic field, and the differential rotation, both of which depend upon the third dimensional aspects of this difficult problem.

Now we discuss photospheric magnetic structures. Magnetic fields on the Sun’s surface are essentially ubiquitous. Namely, despite the appearance of many areas of near zero magnetic field, there are likely only places of vanishing dimension with precisely zero magnetic field. This is related to one aspect of an MHD dynamo: that it takes field to make field. Field is amplified out of pre-existing field. For example, if the field of the Sun were to ever go to zero everywhere, in MHD, it could not be magnified. Field, in non-MHD, can be created from chemical energy, via a battery, but that is outside MHD.

Thus to account for the field everywhere on the Sun, using equally spaced grid elements, one would have to demand a fairly fine spaced grid, and the problem quickly becomes insurmountable. As a result of this, we choose an alternative approach not to be overwhelmed by the regions of very low field. We have chosen the important non-zero surface field be carried by a relatively small number of transport entities. This is a pragmatic approach, since we recognize that whether one uses finite agents or a set of finite grids, nature will always overwhelm humanity with its virtually unlimited number of degrees of freedom, and that we humans, even when aided by computers, are always limited to very few numbers. The exception is when the system can be handled analytically, however, for the real Sun this requires making simplifying idealized assumptions about its behavior. We have undertaken many here, by ignoring small-scales, etc., etc. Nevertheless, in areas of space-physics, a number of people (see Gombosi et al., 2004) have made remarkable advances with adaptive gridded meshes, an avenue showing promise in overcoming nature’s diverse scale size phenomena. Instead of the gridded mesh approach, we have chosen agent-based entities to carry out our analyses. One may regard each agent as a helper-friend, since often they are anthropomorphized as little animals. Since these are essential to our model, we give a detailed account of their development.

Agent-based models arose from the “game of life,” the cellular automata model of the great mathematician, John Horton Conway, first described by Martin Gardner (1970), and then later, came numerous models by Wolfram (1994). Wolfram and associates studied the behavior of many such models, particularly 1D models, dependent upon local parameters, and he really initiated a science from what had been many disparate ideas. To illustrate the diversity, agent-based models have been referred to as agent-based simulations, or multi-agent systems, or other word variations. They combine elements of game theory, complex systems, emergent phenomena, evolutionary programming, as well as Monte-Carlo methods, and other aspects that allow significantly more diversity than Conway, and even Wolfram seem to have imagined. Agent-based models often have a number of the following features: numerous agents specified at various scale sizes (referred to as agent-granularity), decision-making heuristics, interaction rules, and an overall field or domain, upon which the agents move. The granularity may be viewed as changing a continuous differential equation into a number of difference equations, a subtle but important change. Multi-agent systems, however, do not necessarily reside on a gridded domain; hence do not have the advantages, nor the disadvantages, of a continuous geometry.

To actually understand the genesis of multi-agent systems, and also their diversity, we have to go back many years, to the origins of computer languages, the oldest being LISP. This language, along with the more understandable SCHEME version, is discussed in the famous blue Wizard book (SICP) by Abelson, Sussman, and Sussman (1984). Often in computer language discussions, one hears the term “$\lambda$calculus” employed. This was a usage started by Alonzo Church in mathematical logic and the computer sciences. The $\lambda$value is a dummy argument, and one could easily use x, but$\lambda$ is more generic in that it might be a variable, x; if t is a lambda term, and x is a variable, then λx.t is a lambda term (called a lambda abstraction); if t and s are lambda terms, then ts is a lambda term (called an application).The second term, calculus, bears no resemblance to its normal use in the physical sciences implying differentials; it may be taken to mean “computation.” Hence$\lambda$calculus is a generic reference to any computer algorithm in this sense.

The wizard book opened the door, not just to the wider world of computer algorithms, but that of computational logic too, and to the broader “Universe” of how to construct languages and compilers, unfettered by the brittle constraints of most published languages, which despite their great value, they also hamstring programmers into rigid behaviors, such as the need to declare array sizes at the beginning of a program, not allowing programs or subprograms to retain sufficient flexibility so that they can interact with each other, in harmony, but rather must be viewed so that they do not know of each other’s existence, etc. That is, one can only call basic language elements but not other programming subroutines. In lambda calculus, subprograms may be arguments of other subprograms, and functions can call themselves, become embedded, and there seemed to be few if any restrictions, beyond what the writer could make the computer do, from a logical point of view. This was obtained by the recognition that elements passed need not be predefined, and that subprograms or programs only needed to be “tail recursive,” meaning they can have recurrence relationships, and one can simply delay the call to an algorithm until it is needed, or if not, there is no harm. So less restrictive languages have a wonderful “upside,” in that they appear to be boundless.

There are also “downsides,” there is a dark underbelly to them, in that they allow programmers to form open loops which do not end, or otherwise run their program off the rails, etc., but this can happen in many languages. For example, one can create an infinite loop by inserting the two statements: “1 GO TO 2” and “2 GO TO 1” into a FORTRAN routine, however it is injudicious to actually do it. Additionally, the generalized languages do not have all the extra functions that scientific programmers have come to expect, so the languages are not so well tailored for them. Languages then try to strike a balance; some are “typed,” others “untyped,” and each has its own characteristics made to help solve a particular problem.

In any case, with the current wide open field of computer languages, agent-based models have expanded. Cellular automata (agent-based models) have the generous attribute that each agent has certain properties and traditionally each agent only “sees” specific aspects of its environment. Sometimes the term “cellular automata” is used to refer solely to rather simple CA types, wherein they see only local 2D neighbors, which was how they originated, but we and others, usually mean any generic agent-based models. The number of agent-based systems is now apparently too many to name. The interested reader can find books and/or websites containing more information. Early, well-known agent-based systems are Netlogo and Starlogo, distant descendents of LISP; we shall be using the former, as it has a wider variety of flexible attributes, making it more sophisticated, in this author’s eyes. The latter, Starlogo, however, is where the “Boids” algorithm originated. Boids is an arcane reference to the Brooklyn pronunciation of the word birds; the algorithm is one which we utilize to help our field agents transform their relatively random movements towards a directed flow of motion in 2-D.

Both Starlogo and Netlogo, however, have their own quirks. Those with an interest in computer languages may enjoy many hours with each of them, as this author has. Let me say, after a lifetime of frustration with FORTRAN, I now know how computer algorithms should work – enabling their programmers to spend more time creatively rather than sorting out “bugs.” We shall now discuss aspects involving the use of cellular automata, a narrow, but ever widening set of languages that allow “entities” to carry out a set of instructions. Before going into our model, let us examine the value of cellular automata over more traditional analytic solutions to physical problems.

Some of the differences that cellular automata solutions and analytic solutions have are as follows. The two are almost like twin opposites: Tweedle-dee and Tweedle-dum. Let us explain this:

1) We first discuss differential equations (DE). They assume a continuous field, or fields, for their domains, and then specific equations that hold true in this restricted continuous regime (since differential equations require the continuity of the derivative to enable their solutions). Differential equations, when they have solutions are often of the “parabolic type”; these yield much insight, with often exact solutions, although computational solutions are widely used too. Nevertheless, real world differential equations, if they contain “waves” go into the “hyperbolic type” and then analytic solutions cannot be found. So, sometimes one is forced by nature into an unrealistic region, so that a solution is available. For example, one can use diffusion to transport heat, and arrive at the “heat equation”, usually of the Poisson (parabolic) type. Solutions of the heat equation often have a gradual smoothing of the initial temperature distribution by the flow of heat from hot to cold areas, consistent with thermodynamics.
2) Let us now consider cellular automata (CA). They are inherently discrete entities, and they “do the programmer’s bidding”, so they invariably have a “real solution,” unless somehow the code blows up by a poor choice of the programmer in not checking something or doing something “illegal” in the language, say dividing by 0. The point is that the solutions are inherently just a series of “steps”, and the entities just “do as they are told.” In this manner, a solution if it exists is virtually guaranteed. Nevertheless, the downside is that there might not be any understanding beyond saying: I put in A, B, and C and I get out X, Y and Z. To many, this might be unhelpful. It may also not help people who do not know how to “read code” to be told “it is in the code.” Often codes can have mistakes, and the mistakes are buried therein. Many codes are not tested, etc. So, the methodology is somewhat hidden and may not be reproducible, which is why this paper is attempting to have the code published along with the paper. So, everything is transparent, and others may improve or destroy the work based upon what has been accomplished, in black and white.

This is why we say differential equations and cellular automata are essentially opposites. One deals with continuous equations; the other with discrete discontinuous entities. We have considered the behavior of solar magnetism, and consider it more discontinuous than continuous and hence have attempted to improve our understanding using cellular automata. The discontinuous nature is manifestly apparent by the appearance of sunspots in a gaseous medium, having a temperature of many thousands of K.

Let us add a few more words to the above discussion about the differences between DE and CA. Although differential equations can be non-linear, often extravagant non-linearities result in chaotic solutions. This indicates that no general analytic solution exists, except for some vanishingly small solutions (perhaps in the linear regime, infinitely close to a special point). Such chaotic behavior, however, does not restrict CA models, since all their behavior is in the numerical domain, and thus their solutions are inherently numerical, as opposed to being analytical. Numerical does not mean without problems; solutions may grow exponentially thereby overwhelming the most steadfast computer. For example, consider using the simple “shooting method” solution of the differential equation:

$\frac{\text{dx}}{\text{dt}}=\text{ax}$,	(1.1)

given an initial value for x of$x_{0}$, at t=t_0_. Numerically there is a barely hidden problem. The solution becomes unbounded for a>0. If one uses the “shooting method,” of numerical integration, wherein one simply starts off with an initial value at a “boundary,” t=t_0_ , and integrates forward in time, the results will overwhelm any computer if one integrates for a long enough period, and likely one would not have to wait very long indeed! Nevertheless, the analytic solution is the simple equation: $x=x_{0}e^{a(t-t_{0})}$. So, one can find strengths and weaknesses in CA or DE, depending upon the regime employed.

So, in summary, DE have strengths, in that they have analytic solutions, when conditions are suitable, and these analytic solutions indicate they belong to a well-studied discipline within the framework of established mathematics. Analytic solutions, however, are sometimes used in situations where the conditions are not suitable. With cellular automata methods, there is not as much gain by simplifying the model, because the machine can handle complex diversity, with virtually the same ease as simplified mathematics. For example, a CA method can add a thousand different numbers as easily as it is able to add a thousand zeros. So, cellular automata models may be able to gain in diversity what it lacks in elegance. It is a young field, and we do not yet fully understand or appreciate the value of CA methods.

Since the average reader likely has more familiarity with differential equations compared with cellular automata, let us say a little more. Consider the difference in approaches between the equations of heat diffusion and weather systems. The former has plentiful solutions (of the parabolic equations), and CA models add little understanding, but they may help provide solutions in complex cases, like weather systems. In the latter case, only computers grinding on the numbers have been able to churn out numerical short-term predictions, and analytical approaches have not worked. The non-continuous nature of CA may be understood when we recognize that each agent can take undergo any action which it is capable of (move, jump, die, give birth, or any other user-defined algorithm or commands, etc.), if a specific condition holds (e.g. $a\forall F(a,b,c,\text{.}\text{.}\text{.})$), where$\forall$ is a predicate logical symbol, say < for numerical quantities, and F is any function of a number of model parameters: a, b, c,…, each of which may be any local or global quantity or any other agent-based property. If the condition does not hold, any other number of totally different behaviors may be performed, together with any number of various logical functional combinations, limited only by the predicate rules of the language. For example, a single CA model statement may be the following: Consider each agent: if 5 or more of the 13 nearest neighbors is moving away from the agent under discussion, kill every other one, and change some properties of the others, say color them so that they are all blue, have green hair, and will die in 3 time units. As a consequence, there is a rich, seemingly unbounded diversity that such models may enjoy. We do not go this far out on a limb; our agents are rather tame.

With a number of solar phenomena generated suddenly, e.g. flares, sunspots, etc. it may be that gradual equations (amenable to differential equations) are more appropriate for some phenomena and cellular automata for others (generally where conditions, either spatially or temporally, change dramatically). In any case, the use of cellular automata is the path that we have chosen to model solar magnetic fields, in the hope that their usage may lead to new understandings of the non-linear behavior of solar magnetism in the future. We point to the formation of active regions from fields inside the Sun as but one example of a rather abrupt (and likely non-linear) phenomenon that is germane to the subject under investigation. We now describe magnetic fields as agents or automata. For simplicity we simplify the Sun’s magnetism into just two “kinds,” inward and outward field orientations for a number of reasons: 1) this aids the granularity of these agents, and 2) this usage (as opposed to giving each agent a specified amount of flux) allows the fields to completely cancel when two opposite fields interact with each other. We shall use an environment specifically designed for agent-based models, Netlogo. The two different types of agents are called “breeds,” they are basically of identical form, but they can, with this term, recognize any other member as being the same, or a different breed. Our breeds will be generic birds of two varieties, bluebirds and cardinals, with colors blue and red, representing outward pointing, and inward pointing, fields respectively.

We shall be discussing their properties, in particular their births, deaths and motions, but let us first be pragmatic and discuss the Netlogo domain that is their home.

______________________________________

Interface parameters will be discussed throughout, but let us briefly explain what the important control parameters mean, with further discussion of the significant ones. Table 1 provides a list of the adjustable parameters and nominal values used for this run, as well as the fixed parameters. The fixed parameters, with values in parentheses, are: vision (10), avoidance-distance (20), minimum-separation (1), max-align-turn (5), max-cohere-turn (3), and max-separate-turn (9.25). Their meanings are as follows: vision (10) controls how far each bird/field sees in interacting with its extended neighborhood; avoidance-distance (20) how far birds will examine each other, in order to consider separating further, from others of opposite colors to AVOID them. Of course, they do not succeed completely, as the density of birds builds up until the inevitable bird collisions occurs in this model, and opposite color birds both disappear; minimum-separation (1) provides the minimum separation in grid units, of SAME color field lines. Fields turn away to attempt to maintain more spacing than this minimum; it also affects coherence and avoidance because this is done with a higher priority. The priority of these factors may be something for further study; max-align-turn (5) how rapidly the birds can turn towards each other to align, and flow together. The number is the number of degrees per time-step; max-cohere-turn (3) how rapidly the entities can align with cohesion, in degrees per time step; and max-separate-turn (9.25) used in both field separations, in degrees per time-step, as same sign birds spread out from each other, and in avoidance.

Other parameter meanings (shown in Table 1) are: Tick-end stops the run at this time, 0 implies a never-ending run, unless the Go/Stop button is pressed again; random-seed provides an initial seed value – a value of 1 chooses a truly random seed, else the particular seed in the slider bar is the initial (non-random) seed; populationbc is the number of field lines/birds of each color per active region birth; start-poles is the initial number of starting polar field lines at each pole, and must be non-zero; merid-flow is a nominal polar flow rate; cspeed and bspeed are nominal values for typical motion speeds of the cardinal and bluebird species, usually identical; kill-dist is the distance within which opposite color birds will die off if they approach this closely; velocity is a parameter that adjusts the speed of the field motions relative to the fluid, acting on all specie fields; Quad-blaster: a positive value provides negative feedback via reorientation to negate any quadrupole moment of the 2,0 kind and a negative value provides a positive feedback; Npole-degrees is the value above which the polar field is located for the purposes of the program; polar-B-force provides a coupling constant which allows controls the strength that the polar fields reorient opposite sign lower latitude fields (an attractive force for opposite sign fields); Joy’s law is the typical active region tilt (7 degrees), for normal size active regions; AR-rate ; B-DR-rate and C-DR-rate are the B and C coefficients for the differential rotation normalized to unity and the A coefficient is 0, since we are mapping the field in a corotating display of the Sun; Cor (50) controls the strength of the Coriolis acceleration, $-2\omega\times v$, taken as a constant rotation rate ~27 days, however time in our model is uncertain, consequently a parameter is added here, with a nominal 50 setting; DR-mult is a setting that coordinates the rotation rate with the speed at which the model is running owing to the parameter settings – we discuss more below, a nominal 3 was chosen; peak-lat is the highest minimal latitude (that latitude is the beginning of the highest latitude band that active regions will go to – they end 10 degrees poleward of that latitude), so a value of 40 means that the highest band will go from 40-50 degrees; min-lat is the lowest band – going from the lowest value to that value plus 10 degrees (5 is nominal); Butterfly? Controls whether active regions will be born in accord with Spoerer’s Butterfly law, or just be independent of time; All-global-random? controls whether active regions will NOT be oriented randomly on the solar disk – if yes, they are oriented randomly – this is test of the program, but inconsistent with solar behavior, so nominal is NO; several exponential factors reduce the motion at the poles to inhibit motion poleward of 90 degrees: expo, by reducing the geometry; expo2 controls the exponential e-folding rate of bgeometry; and expo3 is the exponential e-folding rate of the polar field’s influence; K2 controls the rate of birthing new active-regions; dipole-or-quad? is again a test for the starting polar field of the Sun –ON is typical, starting the Sun near solar minimum at polar field maximum; patch-color-white? Controls the background display as either black or white – for some purposes it is nice to have a white background; and ssize controls the display size of the field directed motions – the value 0.7 represents an adequate size with which to see the arrows individually, yet not have them totally cover each other up too often, as occurs when the field density gets high; the arrow size does not affect the computations; it is simply a display parameter.

Returning to the model; it incorporates differential rotational and meridional circulation.

The meridional circulation is chosen at ~ .1, corresponding to ~0.1 km/sec. For the differential rotation, at the equator, the Sun rotates with a period near 27 days, and at 33+ days in the Polar Regions. These are traditionally given by the equation:

$\omega(\theta)=A+B\text{cos}^{2}\theta+C\text{cos}^{4}\theta$deg/day,	(1.1.1)

where $\theta$ is the latitude, and $\omega$ is the rotation rate in degrees per day (Newton and Nunn, 1951, Wang and Sheeley, 1994). The quantities A, B and C are the differential rotation parameters. The display is chosen to be co-rotating at the equatorial synodic 27 day rotation rate, close to Carrington’s figure, hence A = 0. As a consequence, the Sun’s equator remains fixed in the display, and fields migrate relative to this reference frame. The other two rotational parameters, B and C, as well as the meridional flow rate are controlled by sliders, with B and C being 1 relative to the Newton and Nunn rate, of 2.4 and 1.8 degrees per day. At 45 degrees latitude, these equate to 1.6 degrees per day. Since the speed of the model in “day units” is unknown to the model, we have provided the DR-rate (chosen at a value 3) slider to adjust the differential rotation to equate (roughly) with the solar cycle. Nevertheless, parameters may be modified significantly depending upon field amplitude and other circumstances as appropriate. The meridional flow is estimated in km/sec, with 0.1 being nominal. Wang and Sheeley (1994) provide B and C, as -2.3 degrees per day, and -1.62 degrees per day, respectively, close to the Newton and Nunn values we employ. If not directed azimuthally, through local interactions with neighbors, this model does not direct fields to drift longitudinally beyond the differential rotation flow. The differential rotation flow is most noticeable at higher latitudes due to the slower rotation rates, where fields of both sign drift to the left.

The coordinate system of the display has the x-coordinate as longitude, and the y-coordinate as latitude, from -90 to +90 degrees. Each "arrow" represents a "unit" of photospheric magnetic flux,$\text{10}^{\text{23}}$ Mx per blue or red arrow, and is oriented in the direction the flux is moving, relative to the fluid. The blue arrows are outward directed flux (aka bluebirds) and the red, inward directed flux (cardinals), most common in solar displays of field. A distribution of shades of color, e.g. pink to deep red, help to distinguish the individual fields and their motions. The arrows show the field motions relative to the fluid.

The two graphs on the right of the interface are of field quantities. Time summaries of the polar fields are shown in Plots A and B. Plot A shows the North polar field (Blue line) and South Polar field (Red line), and total Polar field (Black line) offset by 60 (lower), so as not to interfere with the individual polar fields, and the tick marks at the bottom display the computer calculated Polar field maxima, defined not to be too small an amplitude, nor too soon from the previous polar field maximum. Polar field maxima are closely associated with sunspot minima, and these times are chosen, in this model, as the starting point of a new solar cycle. These times are listed in the monitor Peak-Time-List at the bottom of the interface. These times are chosen as the official peak polar field times, and as the official start of a new solar cycle. This is relevant in that at the start of a cycle, new activity is placed at high latitude bands, from peak-lat, nominally 40 degrees to +10 degrees higher. The latitude bands move equatorward over time, to the min-lat value, nominally 5 degrees as the cycle evolves. In addition the polar fields, monitored throughout the cycle, control the rate of new activity centers born, making these a key element in this model. The parameter npole-degrees, nominally 50 degrees controls the latitude above which polar fields are counted to ascertain the flux present in the polar field, although the high latitude dependence mentioned earlier ensures a smooth integration, without any sharp dependence based upon this parameter. One may, if one chooses, turn off the whole latitude drift associated with butterfly pattern, by turning OFF the Butterfly? Switch in the interface. Normally, one would leave it on.

Plot A shows the North (blue) and South (red) polar averaged field values (approximately poleward of 60 degrees), with time, as well as the total polar flux (black). In addition, is a horizontal line going through zero; this line peaks after the total flux reaches a maximum value. This is used to calculate the time of “solar minimum” (when the polar fields tend to peak), however, delayed by the time needed to examine for peaks, list-length/2. So, for example, if list-length were 5, one would need these 5 values to examine for a peak, and if a peak occurred in the middle, there would be a delay of two ticks from the peak to the present moment of calculation. From this, the latitude of new field BMRs is calculated, in accord with the Butterfly pattern. We may mention time units while we examine this plot. In it, the polar fields oscillate with a rate which varies with amplitude. It takes 4000 ticks to go from the initial 0^th^ peak to the 51^st^ peak at the end of the Figure 1 (bottom). This makes it 78.4 ticks per solar cycle, and considering solar cycles to be about 11 years, this makes the time unit (known as ticks) in our model or ~7.1 ticks/year, or ~51 days/tick unit, in accord with our earlier finding, for a briefer duration of 55 days/tick unit. Thus again, we can repeat that a tick unit (one of our model’s time steps) is ~2 solar rotations.

As mentioned earlier, there is not a complete 1:1 constancy in this, as the Sun’s activity folds in with its “heartbeat,” as one might expect the Sun’s activity does not obey terrestrial ideas of the constancy of time. More will be said about this in a later paper, but it is implicit in Waldmeier’s work that the amplitude of the solar cycle, and the solar cycle duration are linked together, and this can be seen too by examination of Figure 1 (bottom).

Plot B shows a graph of the latitude of new Active Regions (ARs), like a Butterfly diagram, except that, owing to a peculiarity in the plotting program, when no new AR has started, a dot is placed at 0 degrees. If need be, this can be removed by post processing of the plot. Additionally, because the plot is constrained to graph only a single value for each time unit, it does not display the rich pattern of the conventional butterfly diagram (a scatterplot with numerous values for the same abscissa), although the model actually inputs a range of latitudes of new active regions, each forming at different times, but the plot only displays unit timesteps. Nevertheless, the plot does show the drift from high to low latitudes. The equatorial pattern is not so evident in the main field display owing to the Mercator projection resulting in the lower latitudes being compressed, rather than the traditional synoptic map.

Aside from these features, there are numerous sliders in blue, from which input parameters can be changed without reprogramming, as well as a number of white monitors that show individual quantities as time evolves. We now discuss various processes/algorithms which the field entities (birds) are subject to. We later provide more details, but for starters, we simply describe the following behaviors of field entities: their birth, motion and death. Of course we are anthropomorphizing such properties of these cellular automata, which are meant to describe magnetic fields in the photosphere.

In the next subsections we describe agents’ births, motions and deaths. Put succinctly, agents are born, move about on the solar surface by a number of flows and forces we describe, and eventually die. There are a few effects agents have on other agents: 1) while at the poles, field agents are connected via the Babcock-Leighton (B-L) subsurface field to lower latitudes which allows them to undertake two duties there: A) the B-L subsurface field spawns the next generation of new magnetic flux, and B) the B-L subsurface field attracts lower-latitude fields via its long-range magnetic tension; 2) nearby agents affect each other’s motion by short-range interactions; and 3) through deaths: when opposite flux agents get too close to each other, they die off in pairs. We now describe these processes more fully.

1.2.1 Field Entities: Agents and their Birth

In this section, we describe the Agents and their Births, how they come into the 2D world upon which they live. Aside from the initial polar field agents, set by the slider as a positive definite value, agents are born into new “active regions” (ARs), which in this model have zero net flux. The AR consists of a preceding (P) and following (F) group of magnetic fields. The model uses the subroutine add-new-ARs to add a number of new active regions (ARs) based upon the polar field values (deciding how many ARs it should add, and even weighting fractions with an appropriate probability) using random number generators. For each new AR, it calls the add-an-AR routine to birth a new active region. This active region (AR) subroutine obtains the latitude and longitude that a new AR will be situated, from within the active latitude band (governed by the butterfly diagram, if the Butterfly? switch is ON, and global properties that the overarching program has available, from which it ascertains needed information), and otherwise just a broad equatorial band.

In each half of the AR, are birthed an equal number (populationbc) of field entities (bluebirds and cardinals), based upon the sign of the N-S field, and Hale’s law governing preceding and following regions. The new birds are born with a quantized spread of velocities, via speeds chosen by a random number generator, up to the maximum value, bspeed and cspeed, for each the specie. This results in birds having an initial motion, from which the birds join the general flow in a neighborhood, by looking at nearest neighbors, polar fields, etc. We chose an initial velocity with random headings to get the fields to separate from the small bundle, into which they were initialized, considering how small a sunspot is compared to the Sun, thereby allowing the field entities to fill the larger-scale regions of the Sun, as opposed to remain isolated in their initial localized activity center.

The setup initiates a start-poles number of polar fields (birds) at each pole, controlled by the slider bar. Other adjustable parameters are similarly controlled. When dipole-or-quad? is ON, the initial polar field is a large-scale dipole, but when off, then a large-scale quadrupole field is initialized. When Go/Stop is pressed, the model begins birthing new fields from active regions depending upon solar cycle phase, amplitude, and random variables. When the Go/Stop button is pressed again processing stops. It may be pressed repeatedly. Additionally, the processing can be sped up or slowed by the slider bar above the Display on the top of the interface.

The active regions are born in 10 latitude degree wide bands, starting from peak-lat at the beginning of a cycle and ending at min-lat. The arrows in the figures, of course, show the direction of the individual field motions, relative to the fluid. The differential rotation and meridional circulation, being flows, do not affect the arrow-field orientation, but the relative forces, associated with external fields (e.g. polar fields) do.

The first process described is birth; how each entity in the model world originates. Despite using sunspot fields as sources of flux, since this model examines only large-scale fields, the model will NOT actually be showing the smaller, very intense sunspot fields. Our conceptual use of them is merely linguistic shorthand to ascribe how we input new sources of photospheric flux. Sunspot field remnants are placed in pairs of BMRs (bipolar regions), at random longitudes, with latitudes in accordance with the butterfly diagram, with tilts associated with Joy’s law (with a slider bar showing an average tilt of 7 degrees), and a distribution of BMR sizes, with a populationbc number of bluebirds and cardinals per new active region, and with initial speeds having random integer values from 0 up to 2*bspeed and 2*cspeed, respectively. The orientation of BMRs follows Hale’s rules of sunspot polarities, and the number of new active regions per unit time is chosen proportionally to the polar magnetic flux difference, with fractional amounts weighed probabilistically. The model then attempts to consider how all the field lines move about on the solar surface.

Solar observations show, that after sunspot fields break apart, often facular bright points emanate from the sunspot group. Since both faculae and sunspots contain magnetic flux, and this model is basically a flux transport model (although with agent-based entities), the visual appearance of magnetic features is another aspect that is not utilized in this model. We utilize a “pseudo-random field generator” to place new fields on the solar disk, based on the phase of the solar cycle, and the strength of the Sun’s polar fields. This is essentially the Babcock-Leighton (B-L) subsurface field which is assumed to wind around beneath the Sun’s surface. In our model, the fields are placed in highly idealized active regions consisting of dipole pairs of sunspot-like entities (bipolar magnetic regions, BMRs), with a degree of separation, and tilt associated with Joy’s law, and with random equal and opposite fluxes. The various parameters are based upon a statistical distribution.

Returning to field birth, fields/birds first appear as a red or blue arrow, with its orientation shown by the direction of its arrow. Each bird has the following properties: who #, color, heading, x and y coordinates, breed (bluebirds or cardinals), ssize (arrow size used only for display properties), information about nearest neighbors, whether dead or not (to ensure that when two die they do not kill another, before they officially disappear), and who # they are partnered with (to ensure birds die in opposite color pairs), information on their same color flockmates in an agentset, their closest flockmate, their speed, their opposite color flockmates in an agentset, and nearest opposite colored bird, a geometrical factor related to bird size, and the extra parameter “other-nearby” is not actually used). The ‘who #’ is just an identification number unique to each bird. The use of special lists allow many individuals (their who #s) to be placed into a special Netlogo agentset list. This is simply a list of such who #s, which can vary from no members to any finite number. The above parameters have very high accuracy, sufficient to ensure the model is accurately run. For example, the locations are real numbers and have 16 significant digits.

1.2.2 Field Entities: Motions

As discussed earlier, Alfvén’s frozen field approximation, applies to regions of high conductivity; it ensures that the movements of the magnetic field and flow of the conducting fluid are linked together concordantly. This concordance can occur in different ways, depending upon the relative strength of the field and fluid: either the field is sufficiently strong to enforce that the fluid travels along it, or the fluid motions are sufficiently strong so that the field is distended by the flow. In this latter case, the flow can strengthen the magnetic field by stretching or extending it. In such cases, the flow pulls against the field, thereby lengthening it, and the field therein gains energy.

For our model, we take the steady state motions to consist in the meridional flow and the differential rotation. Our model only deals with spatial and temporal differences from these steady state solar motions. The field motions we calculate are not inconsistent with Alfvén’s frozen field approximation; the field motions we calculate are to be associated with small temporal and spatial flows, relative to the predominant large-scale background flows. Differences from the background flows may occur when magnetic field enters the picture, and it is only through the mutual interaction of field and flow that non-zero field and non-zero flow differences are thought to arise. We shall be considering that the field and flow have a tendency to return to “normal,” that is back to their undisturbed states (zero flow relative to the mean flows and zero field). In our model, the field and flow tend towards being “homogenized,” namely having uniform values. Regions of strong magnetic field will have fields spreading outwards from them towards regions of weaker field. The motions we invoke will be such that: 1) they move towards uniformity, and 2) the movements will be such that the field and fluid move together.

In practical terms what we do to establish this second condition is the following. Each field entity calculates the mean motion of its neighbors (within a vision distance). Assuming its motion differs from the neighborhood motion, it adjusts its motion towards that direction in which its neighbors are moving. Fortunately, there existed a Starlogo algorithm, Boids, which had this very behavior we wished to use in our magnetic field model. The behavior is often called “flocking” behavior, as it appears to apply to bird motions, as well as the motions of fish in schools. The individuals respond to either predators or their neighbors searching for food, by following group behaviors where individuals all sample their neighbors. We added further provisos dealing with two species: that unlike fields would move apart, and forces involving the large-scale Babcock-Leighton field, as well as death between two opposite specie members, etc.

The field entities in our model develop spatial and temporal motions that differ from the mean fluid motion. This is a critical aspect of our model. If the fluid and field were to directly follow the mean motions, there would be no clear way to separate new field of opposing polarities from each other. It is through flows that stretch opposing polarity fields, that allow opposing fields to move apart on the Sun, and therein the turbulent fluid does work upon the Sun’s magnetic field. The fluid motions are such that they stretch the magnetic field like a rubber band, as the magnetic field is imbued with the Maxwell stress tension. It was envisioned that such processes manifest themselves predominantly in localized regions of enhanced super-adiabaticity (see Schatten, 2009), aided by the subsurface Babcock-Leighton field, and the enhanced super-adiabaticity near the Sun’s surface.

For the present work, neither the origin nor the detailed mechanism for the Sun’s magnetic activity need be known. Our model simply calculates, according to a set of prescribed algorithms, how the surface fields may move apart, and thus flock into larger-scale entities (unipolar magnetic regions, UMR’s, as envisioned by Bumba and Howard, 1965). Additionally, we have developed a “death” algorithm that prescribes how older fields on the boundaries of these large-scale field entities may annihilate each other, leading to the removal of surface field. One may alternatively view these collisions as magnetic field recombining to sink below the photosphere. Nevertheless, the actual mechanisms involved in both the origins and the disappearances of the Sun’s photospheric fields are somewhat beyond the scope of this paper. Thus, our model is rather blind to the detailed physical sources and sinks of the photospheric field. This model just strives to calculate the motions of the photospheric field, and we supply the simplest births and deaths of these entities as possible. Births are where new field is seen, and deaths occur, when two agents get within a fixed distance,“kill-dist” , apart.

Let us emphasize for clarity, the motions we are calculating are not the general meridional circulation, nor the differential rotation, but the significantly smaller differences from these mean motions in the photosphere. As we shall see in the next section, the motions of our field entities represent motion relative to the mean surface motions of the fluid Sun. This behavior, of having opposite colored field entities, in our model, to move differently, that allows the field to grow and change from background solar field into which they were born. These views allow the motions of field and fluid from their mean values to be calculated.

In particular, the mean field and attendant mean fluid motions appear insufficient to explain the evolution of solar magnetism. If the fluid and field were to directly follow the mean motions, such behavior would indicate the surface fields would not move relative to the background solar field into which they were born. Such fields would be required to die in place, and no changes in the large scale solar fields would exist. Yet we do observe large-scale structural field changes. Thus it is these differences from the static, mean behavior of the field and flow that this model addresses.

How do fields move relative to the background fields of the Sun? Naturally, as mentioned, we utilize the observed meridional circulation and differential rotation as a given, for what the Sun’s field do, in the case of a small test field embedded in the fluid. Our model, thus attempts to explain the movement of the field and fluid from their mean motions.

To further exemplify how fields and flow may move relative to the mean motion, let us examine the following situation. Consider the fields of an active region born in the northern hemisphere during the early phase of a solar cycle. As we explain in the paper, at this stage of a solar cycle, the Babcock-Leighton (B-L) force guides the he following fields of an active region, towards the nearest pole; thus the following field would be directed towards the nearest pole. In this case, the motion is in a north-westerly direction, and the preceding fields travel oppositely, towards the south-east. The motions are similar to those of a “fault-line” in a strike-slip Earthquake, however, without subduction. The oppositely oriented fields would become more sheared, in time, due to the changing field latitudes, and different rotation rates.

Let us provide a brief synopsis of what drives the field’s wanderings in our model. Field entities, in the model, move based upon their velocity. Rather than having forces, which accelerate or decelerate, as in Newtonian physics, the speeds have been chosen as, essentially, fixed, at the time the field entity was born, based upon a random number distribution, and controlled by the cspeed and bspeed slider bars. The forces, instead of altering the vector velocity, only affect the orientation of the velocity, and not the magnitude. This was deemed advantageous for a variety of reasons; however, it may be something desirable to change in the future. A wide variety of forces exist, which redirect the field entities:

1) Large Scale Motions:

1.1) Differential Rotation: wherein the field motions, in addition to the other aspects of their behavior, have a drift in accordance with the Newton and Nunn rotation rates on the surface of the Sun (the equator is chosen to show no rotation, which is done simply by setting the Differential Rotation A parameter, which controls the rigid body rotation of the Sun, to 0);

1.2) Meridional Circulation: wherein field lines move on the solar surface, carried by a surface flow from equator to poles. The exact value is controlled by a slider merid-flow.

1.3) Coriolis Acceleration: Taken as $-2\omega\times v$ with a constant rotation rate ~27 days; however time in our model is variable, consequently a parameter Cor(50) is used. As on the Earth, in the northern solar hemisphere, flow velocities accelerate such that, for example, in a high pressure region, an anticyclone forms rotating in a clockwise direction, and vice-versa in the south. Thus, for the Earth, low pressure regions, like hurricanes rotate anti-clockwise in the northern hemisphere. For the Sun, the acceleration becomes significant; at v~5 m s^-1^, one finds for the duration of a solar rotation, the velocity vector rotates around a clockwise rotational radius of ~2 x 10^5^ km; this is, of course, dependent upon latitude;

2) Flocking motions (these were originally controlled by parameters and slider bars, however, with growing numbers of solar parameters, these flocking parameters are now fixed in the program, but can be modified by changing the program):

1.1) Coherency and Alignment: Patterned after bird flocking, but with like-fields tending to move together, but additionally spread outwards, by a feature that allows the fields to “look-ahead,” and thereby tend to move from regions of higher field strength to areas of lower field strength, thus spread out uniformly, as the field of single sign flux would do (e.g. numerous monopoles). VISION is the distance that each bird can see 360 degrees around it, to identify neighbors either of same or opposite color;

1.2) Separation: wherein opposite fields move apart from each other, so as to evade death, however, if they were all to avoid death, numbers of birds would grow and then eventually be under increasingly closer confinement and deaths would inevitably rise until births and deaths became balanced on average. Three TURN-ANGLE parameters control the maximum angle the birds can turn, within a time step unit, as a result of each rule;

3) Birth: wherein new active regions are born in "low latitudes," proportional to the polar field strength, and in accord with "Hale's laws” of sunspot polarities and active region tilts- “Joy’s law";

4) Death: if opposite color field lines get within a distance of "kill-dist" from each other, they both die;

5) Polar Field Tension (the polar-B-force), wherein each field line is attracted by the tension of the subsurface Babcock-Leighton field lines, which generally wind around the Sun, from the North to the South Polar Regions. The field tension can be removed by using the slider-bar, polar-B-force, to zero, removing this most important guiding force, in this model. The toroidal field is not used in the current model, as differential rotation essentially provides a predominant longitudinal spreading of fields. As with other guiding forces (e.g. interaction of surrounding fields), unlike fluid drifts, the forces interact on each field by reorienting them minutely, within algorithm substeps.

Other processes and settings, also have "slider bars" describe the following: list-length (5) controls the number of time steps (ticks) that the algorithm considers to ascertain if a polar field peak has occurred; bird-reps (1) relates to how many steps bird motions may occur before changing direction; Quant (5) helps affect the amount of quantization in agent motions; and gcf (0.225) is set to this value to achieve a great-circle path, rather than a rhumb line for entities moving with zero forces.

Let us mention that one may adjust the model’s speed by the slider bar at the very top of the Netlogo Interface. We now describe in greater detail, the aspect of field loss or bird death. Some of these behaviors may be best understood by examining the model running, and observing disappearing field entities, for example.

1.2.3 Field Entities: Deaths

In the current model, each entity is born, moves across the solar disk, and eventually disappears, which we call death, when it encounters an opposite field entity/bird. On average, there is a balance between life and death. Birth is simply a life that is “owed to death” in the future. Real magnetic fields may spread outward into larger regions, hence weaken as the magnetic field is defined as the magnetic flux per unit area, thus real fields are virtually impossible to be totally destroyed when they exist on a large surface, in the framework of the classical theory of fields. By definition a field has a value at every point, and in the real world, these values are most often never exactly zero. In the mathematical world of the present model, however, we define death as happening whenever two fields of opposite sign come closer than a finite kill-dist apart. Because our agents carry equal field magnitude, this aspect reduces the overall mathematical problem from differential equations essentially to finite difference equations, making the task considerably easier. This results in no imbalance or net flux remaining when agents die.

What would happen if the two fluxes were unequal? One would be required to partially cancel fluxes. As a consequence, there would not be as great a complete cancellation as is done here. Instead agents would continuously be required to have smaller and smaller amounts of unbalanced flux and these little units would run into each other with ever-increasing frequency, and so a Gaussian-type distribution tail would ensue. This would be the computer’s attempt to move into the world of a continuous differential equation, and the computer cannot solve this problem. Hence, this would result in system overload from having to deal with ever increasing numbers of “tiny agents.” To avoid this problem, we choose the SAME route that nature has indeed chosen on the microscopic level, namely quantization, which governs the atomic world.

We observe the inherent beauty of nature, and recognize that it behaves more cleverly than we would have chosen. Using the wisdom of nature as a guide, we quantize the magnetic flux into units, and allow our field lines to carry only these quantized amounts. For convenience, and because we do not have all the resources of the Sun, our quantized unit of magnetic flux is value is $\text{10}^{\text{23}}$ Mx. Nature may not need to quantize things, in order to undertake its calculations, nevertheless, it seems to help our model, but removing the burden of dealing with an infinite number of infinitesimal field fragments

As mentioned, the death process has an important role in this program to limit the growth of living entities. A corresponding, but real, process may similarly help the actual Sun, by preventing the magnetic flux on the Sun’s surface from growing too large. In this model, if the global field grows larger, then more and more agents of opposite signs annihilate and the overall flux is reduced. Thus an upper limit on the model’s global flux occurs. Perhaps for the Sun too, this may be one stabilizing factor that limits the overall level of the Sun’s surface fields. Naturally, physical aspects such as plasma pressure and conductivities play important limiting roles too.

; SOLAR FIELD MODEL INTERFACE PARAMETERS AND USER CODE:

; Table 1.THE PARAMETERS ARE INVOKED BY SPECIAL SLIDERS (WHICH

; CAN EASILY VARY FROM THE START VALUES USED IN THE TABLE	; ; BELOW), BUT WE CANNOT PLACE THIS INFO IN READABLE FORM.	;THEY ;CAN BE READ IN, BY THE NETLOGO PROGRAM ATTACHED.

| Interface Parameter | Nominal or Sample Value |
| --- | --- |
| Tick-end | 200; 0 for continuing calculation |
| Random-seed | 314159 or other #; 1 for random runs |
| Populationbc | 20 |
| Start-poles | 20 |
| Merid-flow | 0.1 |
| Cspeed | 4 |
| Kill-dist | 2.9 |
| Bspeed | 4 |
| Velocity | 4 |
| Quad-blaster | 200 |
| Npole-degrees | 50 |
| Polar-B-Force | 50 |
| Joy’s-Law | 7 |
| AR-rate | 40 |
| B-DR-rate | 1 |
| C-DR-rate | 1 |
| DR-mult | 0.2 |
| Butterfly? | On |
| All-Global-Random? | Off |
| Expo | 8 |
| Expo2 | 0.5 |
| Expo3 | 1 |
| Quant | 5 |
| gcf | 0.225 |
| Peak-lat | 40 |
| Min-lat | 5 |
| K2 | 3.1 |
| Dipole-or-quad? | On |
| Patch-color-white? | Off |
| Ssize | 0.7 |
| DR-mult | 0.2 |
| List-length | 5 |
| Bird-reps | 1 |

; SFM USER CODE/ PROCEDURES OF SOLAR FIELD MAPPING MODEL 1p07

globals [mean-blue-y mean-card-y AR-location-x AR-location-y count-dead npole-lat spole-lat

npole spole tilt quad sign-quad temp temp2 AR-count bcfield-count n-s-pole n-s-pole-old

abs-pole tempxx tempy old-tick mod-tick vision avoidance-distance minimum-separation max-align-turn max-cohere-turn

max-separate-turn off-track short-list s-list center-peak? new-value central-position

average-Bp peak-Bp peak-time K1 solar-cycle-length average-cycle-length delta-lat lat-AR lat-AR-last peak-time-list lat-min-time

old-npole old-spole Ncycles ]

breed [bluebirds bluebird	]

breed [cardinals cardinal]	; cardinals are red

bluebirds-own [ blue-flockmates nearest-blue-neighbor bbspeed card-flockmates nearest-card-neighbor bgeometry other-nearby]

; I think it was all fine... i just had extra thing defined blue-neighbors in card own, but not used..

;; the cardinals own seems like it was incorrect!!!!!!!;;; i have blue-neighbors in cards own, but not vice versa... so removing

;blue-neighbors in cards-own..

cardinals-own [ blue-flockmates nearest-blue-neighbor ccspeed card-flockmates nearest-card-neighbor cgeometry other-nearby]

turtles-own [ tempx phi theta distance-from-card nearest killed? partner]

to setup

clear-all

setup-patches

if random-seed-1=YES != 1

[ random-seed random-seed-1=YES ]

set off-track 0

set Ncycles 0

set vision 10

set avoidance-distance 20

set minimum-separation 1

set max-align-turn 5

set max-cohere-turn 3.25

set max-separate-turn 9.25

set mod-tick 1

set abs-pole .1

set AR-count 0

set n-s-pole 0

set n-s-pole-old 0

set short-list n-values list-length [0]

set central-position int (list-length / 2 )

set peak-Bp 40

set peak-time 0

set lat-min-time 0

set delta-lat 10

set average-cycle-length 20

set average-Bp 40

set K1 (peak-lat - min-lat) / average-cycle-length

set peak-time-list []

set bcfield-count 0 ; count blues and reds

set count-dead 0

set npole-lat npole-degrees * max-pycor / 90 ; ~60 deg

set spole-lat 0 - npole-lat

; set gcf 00.0

ifelse All-Global-Random? [

create-random-dipole

]

[ ifelse dipole-or-quad?

[ create-cardinals start-poles ;populationc

[	set killed? false

set partner nobody

set bcfield-count bcfield-count + 1

set color red - 2 + random 7 ;; random shades look nice

set size .5 * ssize * ( 1.01 / ( .01 + abs ( sin ( .9)))) ^ .5 ;; easier to see

setxy random (2 * max-pxcor) max-pycor * -0.99 ] ;random-xcor random-ycor ]

create-bluebirds start-poles ;populationb

[	set killed? false

set partner nobody

set bcfield-count bcfield-count + 1

set color blue - 2 + random 7 ;; random shades look nice

set size .5 * ssize * ( 1.01 / ( .01 + abs ( sin ( .9)))) ^ .5 ;; easier to see

setxy	random (2 * max-pxcor) max-pycor * 0.99] ; random-xcor random-ycor ]

]

; poles:

[	create-cardinals start-poles / 2 ;populationc

[	set killed? false

set partner nobody

set color red - 2 + random 7 ;; random shades look nice

set size .5 * ssize * ( 1.01 / ( .01 + abs ( sin ( .9)))) ^ .5 ;; easier to see

setxy random (2 * max-pxcor) max-pycor * -0.99 ]

create-cardinals start-poles / 2 ;populationc

[	set killed? false

set partner nobody

set color red - 2 + random 7 ;; random shades look nice

set size .5 * ssize * ( 1.01 / ( .01 + abs ( sin ( .9)))) ^ .5 ;; easier to see

setxy random (2 * max-pxcor) max-pycor * 0.99 ] ;random-xcor random-ycor ]

create-bluebirds start-poles ;populationb here fields are being added to equator..

[	set killed? false

set partner nobody

set bcfield-count bcfield-count + 1

set color blue - 2 + random 7 ;; random shades look nice

set size .5 * ssize * ( 1.01 / ( .01 + 0)) ^ .5 ;; easier to see

setxy	random (2 * max-pxcor) 0 ]

]]

do-plots

end

to ask-Bp ; does nothing now..

end

to create-random-dipole

set tempxx random (2 * max-pxcor)

set tempy max-pycor * ( asin (( random 200 - 100) / 100 ) / 90 )

create-cardinals 1 [

set killed? false

set partner nobody

set ccspeed (random (2 * Quant * cspeed)) / Quant

set bcfield-count bcfield-count + 1

set color red - 2 + random 7 ;; random shades look nice

set size .5 * ssize * (1.01 / ( .01 + abs ( sin (tempy / max-pycor)))) ^ .5

setxy tempxx tempy ] ;random-xcor random-ycor ]

create-bluebirds 1 [

set killed? false

set partner nobody

set bbspeed (random (2 * Quant * bspeed)) / Quant

set bcfield-count bcfield-count + 1

set color blue - 2 + random 7 ;; random shades look nice

set temp random 360

set temp2 (tempy + cos temp)

if (temp2 > max-pycor) [set temp2 max-pycor]

if (temp2 < (0 - max-pycor)) [set temp2 (0 - max-pycor)]

setxy (tempxx + sin temp) ( temp2)

set size .5 * ssize * ( 1.01 / ( .01 + abs ( sin (temp2 / max-pycor)))) ^ .5

]

end

to setup-patches

if patch-color-white? [ask patches [ set pcolor white ]]

end

to kill

if (abs-pole > 2 and killed?) [set count-dead count-dead + 1 die]

end

to fluid-flow

diff-rot

meridional-flow

end

to bird-flock

end

;(PUT ALL BIRD Flocking behavior here, and shorten elsewhere?)

to go

if (TICK-END != 0) [if (ticks >= TICK-END) [stop]]

fluid-flow

ask cardinals [ card-flock ]

ask bluebirds [blue-flock]

ask bluebirds [blue-coriolis-accel]

ask cardinals [red-coriolis-accel]

fluid-flow

ask cardinals [blue-avoid ]

ask bluebirds [card-avoid]

fluid-flow

ask cardinals [cgeom ]

ask bluebirds [bgeom]

; fluid-flow

repeat bird-reps

[ask bluebirds [blue-Great-Circle-Force] ask bluebirds [fd velocity * bbspeed * bgeometry * (abs-pole + 15) ^ expo3 / 10000 ] ; was (abs-pole * 50)]

ask cardinals [red-Great-Circle-Force] ask cardinals [fd velocity * ccspeed * cgeometry * (abs-pole + 15) ^ expo3 / 10000 ] display ] ; was (abs-pole * 50)] display	]

fluid-flow

ask cardinals [ set size .5 * ssize * ( 1.01 / ( .01 + abs ( sin (ycor / max-pycor)))) ^ .5 ]

ask bluebirds [ set size .5 * ssize * ( 1.01 / ( .01 + abs ( sin (ycor / max-pycor)))) ^ .5 ]

; fluid-flow

set mean-card-y 0 ;(mean[ ycor] of cardinals)

set mean-blue-y 0;(mean[ ycor] of bluebirds)

fluid-flow

if (ticks mod mod-tick = 1) [pole-count]

ask turtles [polar-drift]

repeat 1 [fluid-flow]

ask cardinals

[if (nearest-blue-neighbor != 0 )

[ if ( nearest-blue-neighbor != nobody )	; was 2-

[ if (abs-pole > 2 ) [ if ( (cgeometry ^ 0.1) * distance nearest-blue-neighbor < kill-dist) and (partner = nobody)

[ set partner nearest-blue-neighbor

ask partner [

set partner myself

set count-dead count-dead + 1	die	]

set count-dead count-dead + 1	die

]

]

]

]

]

repeat 1 [fluid-flow]

if ( off-track = 0 ) [add-new-ARs]

repeat bird-reps [ ask cardinals [red-Great-Circle-Force] ask cardinals [fd velocity * ccspeed * cgeometry * (abs-pole + 15) ^ expo3 / 10000 ]

ask bluebirds [blue-Great-Circle-Force] ask bluebirds [fd velocity * bbspeed * bgeometry * (abs-pole + 15) ^ expo3 / 10000 ] display	]

ask bluebirds [ blue-flock ]

ask cardinals [card-flock]

repeat bird-reps [ask cardinals [red-Great-Circle-Force] ask cardinals [fd velocity * ccspeed * cgeometry * (abs-pole + 15) ^ expo3 / 10000 ]

ask bluebirds [blue-Great-Circle-Force] ask bluebirds [fd velocity * bbspeed * bgeometry * (abs-pole + 15) ^ expo3 / 10000 ] display	]

;; repeat 10 [ wait 0.5 ]

repeat 1 [ fluid-flow]

ask bluebirds [card-avoid ]

ask cardinals [blue-avoid]

repeat bird-reps [ask cardinals [red-Great-Circle-Force] ask cardinals [fd velocity * ccspeed * cgeometry * (abs-pole + 15) ^ expo3 / 10000 ]

ask bluebirds [blue-Great-Circle-Force] ask bluebirds [fd velocity * bbspeed * bgeometry * (abs-pole + 15) ^ expo3 / 10000 ] display	]

fluid-flow

ask bluebirds [bgeom ]

ask cardinals [cgeom]

repeat bird-reps [ ask cardinals [red-Great-Circle-Force] ask cardinals [fd velocity * ccspeed * cgeometry * (abs-pole + 15) ^ expo3 / 10000 ]

ask bluebirds [blue-Great-Circle-Force] ask bluebirds [fd velocity * bbspeed * bgeometry * (abs-pole + 15) ^ expo3 / 10000 ] display	]

;2 fluid-flow

repeat bird-reps [ask cardinals [red-Great-Circle-Force] ask cardinals [fd velocity * ccspeed * cgeometry * (abs-pole + 15) ^ expo3 / 10000 ]

ask bluebirds [blue-Great-Circle-Force] ask bluebirds [fd velocity * bbspeed * bgeometry * (abs-pole + 15) ^ expo3 / 10000 ] display	]

fluid-flow

pole-count

set abs-pole (abs npole + abs spole + .1)

ifelse (abs-pole > 100 + abs spole + abs npole )

[set off-track 1] ;;; problem shooting off to infinity..

[set off-track 0]

ask turtles [polar-drift]

fluid-flow

add-new-ARs

tick

set new-value abs-pole

set short-list lput new-value but-first short-list

set s-list map [round ?] short-list

set-central-peak?

ifelse (abs npole = 0 or abs spole = 0) []

[ if (abs npole > 0 or abs spole > 0) [

ifelse (center-peak? and (npole / abs npole = 0 - spole / abs spole) and (abs (npole - spole) > 8 and

(ticks - peak-time) > 10 ) and (ticks > 30 or sign npole != old-npole and sign spole != old-spole)

)

[ set old-npole sign npole

set old-spole sign spole

set Ncycles Ncycles + 1

set peak-Bp max short-list

set peak-time (ticks - (list-length / 2))

set peak-time-list lput (1 + int peak-time) peak-time-list

set lat-min-time -60 + 10 ;;; was60 ;CHANGED

]

[set lat-min-time -60]

]]

fluid-flow

do-plots

quad-blaster1

end

to blue-Great-Circle-Force

; change the heading amount to same as velocity:

;velocity * bbspeed * bgeometry * (abs-pole + 15) ^ expo3 / 10000

ifelse (heading < 180 ) [set heading heading	+

velocity * bbspeed * bgeometry * (abs-pole + 15) ^ expo3 / 10 * gcf ^ 0.8 * (tan (ycor / max-pycor)) / (cos (ycor / max-pycor)) ]

[set heading heading	-

velocity * bbspeed * bgeometry * (abs-pole + 15) ^ expo3 / 10 * gcf ^ 0.8 * (tan (ycor / max-pycor)) / (cos (ycor / max-pycor)) ]

end

to red-Great-Circle-Force

ifelse (heading < 180 ) [set heading heading	+

velocity * ccspeed * cgeometry * (abs-pole + 15) ^ expo3 / 10 * gcf ^ 0.8 * (tan (ycor / max-pycor)) / (cos (ycor / max-pycor)) ]

[set heading heading	-

velocity * ccspeed * cgeometry * (abs-pole + 15) ^ expo3 / 10 * gcf ^ 0.8 * (tan (ycor / max-pycor)) / (cos (ycor / max-pycor)) ]

end

to set-central-peak?

ifelse (max short-list = item central-position short-list)

[set center-peak? true ]

[set center-peak? false ]

end

to bgeom

set bgeometry (1 + abs (sin (90 * ycor / max-pycor ) * cos (90 * ycor / max-pycor ) / .5) ) * ((max-pycor - abs ycor) / max-pycor) ^ ( 2 * EXPO2)

end

to cgeom

set cgeometry (1 + abs (sin (90 * ycor / max-pycor ) * cos (90 * ycor / max-pycor ) / .5) ) * ((max-pycor - abs ycor) / max-pycor) ^ ( 2 * EXPO2)

end

to add-new-ARs

ifelse All-Global-Random? [

create-random-dipole

]

[ set solar-cycle-length (average-cycle-length * average-Bp / (1 + peak-Bp))

; fine

set temp2 AR-rate * K2 * abs-pole * (1 - (cos (90 * (ticks - peak-time) / solar-cycle-length)) ^ 2) / (2 * 2000)

;( 1. - 0.5) / ( solar-cycle-length * 2 * 2000)

;(1 - (cos (90 * (ticks - peak-time)) ^ 2 / solar-cycle-length)) / (2 * 2000)

ifelse (temp2 > 1 )

[ repeat temp2 [ add-an-AR]]

[ifelse (random 200 < temp2 * 200 ) [ add-an-AR] [dont-add-AR]]]

end

to dont-add-AR

set lat-AR 0

end

to add-an-AR

set AR-count AR-count + 1

set AR-location-x random (2 * max-pxcor)

ifelse butterfly?

[set temp2 peak-lat - (ticks - peak-time) * K1

if (temp2 < 0) [set temp2 0]

set temp (2 * (random 2) - 1 ) * (temp2 + random delta-lat) ]

[set temp (random 60 - 30)] ;; this is 30 to -30

set lat-AR-last lat-AR

set lat-AR temp

set AR-location-y ( temp) * max-pycor / 90

set tilt temp * Joy's-law / 20

create-cardinals populationbc [

set killed? false

set partner nobody

set ccspeed (random (2 * Quant * cspeed)) / Quant

set bcfield-count bcfield-count + 1

set color red - 2 + random 7

set temp sign AR-location-y * random 3

setxy AR-location-x - temp * sign(n-s-pole)	AR-location-y

set size .5 * ssize * (1.01 / ( .01 + abs ( sin (AR-location-y / max-pycor)))) ^ .5 ]

create-bluebirds populationbc [

set killed? false

set partner nobody

set bbspeed (random (2 * Quant * bspeed)) / Quant

set bcfield-count bcfield-count + 1

set color blue - 2 + random 7 ;; random shades look nice

set size .5 * ssize * (1.01 / ( .01 + abs ( sin ((AR-location-y + 2 * temp * tilt / 57.3) / max-pycor)))) ^ .5

setxy AR-location-x + temp * sign(n-s-pole) (AR-location-y - 2 * temp * tilt / 57.3)

]

end

;; w = a + b sin^2 theta + c sin^4 theta

; a = 14.7 b= - 2.4 c = -1.8 degrees per day

to diff-rot

ask turtles

[	; the 14.7 is removed, because we insist upon no DR at equator..

set temp2 (sin (90 * ycor / max-pycor )) ^ 2

set tempx xcor - DR-mult * ( B-DR-rate * 2.4 * temp2 + C-DR-rate * 1.8 * temp2 ^ 2)

set xcor tempx

; render-turtle

]

end

to meridional-flow

;; yields an error below at asin...

; merid-flow is towards poles... proportional to sin lat cos lat

; lat is asin ycor

ask turtles

[

if (abs ycor < max-pycor) [

set temp asin (ycor / max-pycor) ; -90 to + 90

set temp2 temp + merid-flow * sin temp * cos temp

set ycor max-pycor * sin temp2

]]

end

to quad-blaster1

ifelse (sign npole = sign spole) [set quad 1] [set quad 0 set sign-quad 0]

if (quad = 1) [ set sign-quad sign npole

ask turtles [ equator-drift ] ]

end

to equator-drift

;; fractionally turn heading!!

if (quad-blaster != 0) ; WAS if polarB-wind?

[ ifelse (breed = bluebirds)[ set heading heading + sign ycor * sign-quad * sin heading * polar-B-force * quad-blaster / 3000]

[ set heading heading - sign ycor * sign-quad * sin heading * polar-B-force * quad-blaster / 3000]

]

end

to polar-drift

;; fractionally turn heading!!

if ( polar-B-force != 0)

[ ifelse (breed = bluebirds)[ set heading heading + (npole - spole) * sin heading * polar-B-force / 1000]

[ set heading heading	-	(npole - spole) * sin heading * polar-B-force / 1000]

]

end

to pole-count

set npole 0

set spole 0

; for geometry, with an exponential number of 8, the mean factor above 50 degrees is 1.55 a normalizing factor

; that counts the field for 1 pole as 1 field line..

ask bluebirds [if ycor > npole-lat [set npole npole + 1.55 * (ycor / max-pycor) ^ expo]]

ask bluebirds [if ycor < spole-lat [set spole spole + 1.55 * (abs ycor / max-pycor) ^ expo]]

ask cardinals [if ycor > npole-lat [set npole npole - 1.55 * (ycor / max-pycor) ^ expo]]

ask cardinals [if ycor < spole-lat [set spole spole - 1.55 * (abs ycor / max-pycor) ^ expo]]

set n-s-pole-old n-s-pole

set n-s-pole npole - spole

end

to find-distance

set distance-from-card distancexy 0 0 ; or xcor ycor ; or 0 0

; (distance (xcor-of 0) (ycor-of 0))

end

to-report sign [number]

ifelse number >= 0

[ report 1 ]

[ report (- 1) ]

end

to do-plots

set-current-plot "plot A"

set-current-plot-pen "npole"

plot npole

set-current-plot-pen "spole"

plot spole

set-current-plot-pen "lat-min-time"

plot lat-min-time

set-current-plot-pen "abs-pole - 60"

plot (abs-pole - 60)

set-current-plot "plot B"

set-current-plot-pen "lat-AR"

; set-current-plot-pen "lat-min-time"

; plot lat-min-time

; ifelse (lat-AR = lat-AR-last)

;[set lat-AR 99]

;[plot lat-AR]

plot lat-AR

end

to blue-coriolis-accel;; turtle procedure

;; fractionally turn heading!!

set heading heading - 2 * Cor * (ycor / max-pycor) * bspeed / 1000 ; ; speed based on indiv motion.

end

to red-coriolis-accel;; turtle procedure

;; fractionally turn heading!!

set heading heading - 2 * Cor * (ycor / max-pycor) * cspeed / 1000 ; speed based on indiv motion.

end

to blue-flock ;; turtle procedure

find-blue-flockmates

if any? blue-flockmates

[ find-nearest-blue-neighbor

ifelse distance nearest-blue-neighbor < minimum-separation

[ separate-blue ]

[ align-blue

cohere-blue ] ]

end

to card-flock ;; turtle procedure

find-card-flockmates

if any? card-flockmates

[ find-nearest-card-neighbor

ifelse distance nearest-card-neighbor < minimum-separation

[ separate-card ]

[ align-card

cohere-card ] ]

end

to blue-avoid ;; turtle procedure

find-blue-flockmates

if any? blue-flockmates

[ find-nearest-blue-neighbor

ifelse (distance nearest-blue-neighbor) < AVOIDANCE-DISTANCE * cgeometry

[ separate-blue ]

[ ] ]

end

to card-avoid ;; turtle procedure

find-card-flockmates

if any? card-flockmates

[ find-nearest-card-neighbor

ifelse (distance nearest-card-neighbor) < AVOIDANCE-DISTANCE * bgeometry

[ separate-card ]

[ ] ]

end

to find-blue-flockmates ;; turtle procedure

set blue-flockmates other bluebirds in-radius vision

end

to find-card-flockmates ;; turtle procedure

set card-flockmates other cardinals in-radius vision

end

to find-nearest-blue-neighbor ;; turtle procedure

set nearest-blue-neighbor min-one-of blue-flockmates [distance myself]

; set nearest nearest-blue-neighbor [distance-from-card]

end

to find-nearest-card-neighbor ;; turtle procedure

set nearest-card-neighbor min-one-of card-flockmates [distance myself]

end

;;; SEPARATE

to separate-blue ;; turtle procedure

turn-away ([heading] of nearest-blue-neighbor) max-separate-turn

end

;;; ALIGN

to align-blue ;; turtle procedure

turn-towards average-blue-flockmate-heading max-align-turn

end

to-report average-blue-flockmate-heading ;; turtle procedure

;; We can't just average the heading variables here.

;; For example, the average of 1 and 359 should be 0,

;; not 180. So we have to use trigonometry.

let x-component sum [sin heading] of blue-flockmates

let y-component sum [cos heading] of blue-flockmates

ifelse x-component = 0 and y-component = 0

[ report heading ]

[ report atan x-component y-component ]

end

;;; COHERE

to cohere-blue ;; turtle procedure

turn-towards average-heading-towards-blue-flockmates max-cohere-turn

end

to-report average-heading-towards-blue-flockmates

let x-component mean [sin (towards myself + 180)] of blue-flockmates

let y-component mean [cos (towards myself + 180)] of blue-flockmates

ifelse x-component = 0 and y-component = 0

[ report heading ]

[ report atan x-component y-component ]

end

;;; SEPARATE

to separate-card ;; turtle procedure

turn-away ([heading] of nearest-card-neighbor) max-separate-turn

end

;;; ALIGN

to align-card ;; turtle procedure

turn-towards average-card-flockmate-heading max-align-turn

end

to-report average-card-flockmate-heading ;; turtle procedure

let x-component sum [sin heading] of card-flockmates

let y-component sum [cos heading] of card-flockmates

ifelse x-component = 0 and y-component = 0

[ report heading ]

[ report atan x-component y-component ]

end

;;; COHERE

to cohere-card ;; turtle procedure

turn-towards average-heading-towards-card-flockmates max-cohere-turn

end

to-report average-heading-towards-card-flockmates ;; turtle procedure

;; "towards myself" gives us the heading from the other turtle

;; to me, but we want the heading from me to the other turtle,

;; so we add 180

let x-component mean [sin (towards myself + 180)] of card-flockmates

let y-component mean [cos (towards myself + 180)] of card-flockmates

ifelse x-component = 0 and y-component = 0

[ report heading ]

[ report atan x-component y-component ]

end

;;; HELPER PROCEDURES

to turn-towards [new-heading max-turn] ;; turtle procedure

turn-at-most (subtract-headings new-heading heading) max-turn

end

to turn-away [new-heading max-turn] ;; turtle procedure

turn-at-most (subtract-headings heading new-heading) max-turn

end

;; turn right by "turn" degrees (or left if "turn" is negative),

;; but never turn more than "max-turn" degrees

to turn-at-most [turn max-turn] ;; turtle procedure

ifelse abs turn > max-turn

[ ifelse turn > 0

[ rt max-turn ]

[ lt max-turn ] ]

[ rt turn ]

end
