# Supplementary material for: Large-scale solar magnetic field mapping: I
Source: Springerplus. 2013 Jan 23;2:21. doi: 10.1186/2193-1801-2-21 (PMC3601253; doi:10.1186/2193-1801-2-21)
Supplement: Supplementary file 1 — Additional file 1: Movie 1: Shown is the Display section of the Solar Field Mapping model’s Interface vs. time for about 2 solar cycles. The movie can be viewed with Apple’s Quicktime movie viewer. It was made with a model identical to the model described in this paper, but written so as to allow movies to be made into a mov file. The movie can be stopped, or played at a slower speed with the Quicktime movie viewer. Movie 2. Similar to movie 1, however, the time period of the movie is extended; this allows one to see many solar cycles in the Display mode. (ZIP 2 MB) [file 40064_2012_134_MOESM1_ESM.zip › Readme.doc]

Electronic Supplementary Material for Solar Field Mapping Program (SFM).

This is the readme file.

This provides information about opening and running the SFM program.

The files consist of :

1 This readme file;

2 the Netlogo program, ending with the extension: .nlogo:

Namely: Solar Field Mapping 1p07.nlogo

3 The solar field mapping applet: Solar Field Mapping 1p07.html

4 which uses netlogo’s applet: Netlogolite.jar

for running the html code,

and 5 the Solar Field Mapping Model Information and Code

IF one wants to have more capability (for changing code etc.), then one might wish to use the full Solar Field Mapping 1p07.nlogo program.

This REQUIRES the FULL installation of netlogo (not a big thing). It is available

from the Netlogo site: <http://ccl.northwestern.edu/netlogo/>

at Northwestern. It was compiled with a download of Netlogo 4.1.2, or it should be runnable on a more modern version, hopefully.

Or one may use the html applet: Solar Field Mapping 1p07.html program

with the attached Netlogolite (the jar file) program (just place both files in the same folder and click on the Solar Field Mapping program, and it should run.).

One just clicks the html program, and waits a minute for it to load, and then press

Setup

And the Go/Stop

Buttons.. and it should go on merrily for 400 ticks. The time can be increased to a longer

Interval by the slider bar in the screen.

Changing the time to 0 ticks will allow the program to run indefinitely, hopefully, or

until the power runs out, Or something bad happens, which I have not witnessed.

Helpful information.

The netlogo program will display the code, should you choose to examine it, and

will also provide some extra information..

Should any information be confusing about any of this, the

Author, Kenneth Schatten, may be contacted, at [ken.schatten@ai-solutions.com](mailto:ken.schatten@ai-solutions.com)

Or [kschatten@alum.mit.edu](mailto:kschatten@alum.mit.edu) or other means. At this moment in time, happy to work

With any serious solar scientist about future work in this area.
